# The Backbone of Regionalisation: Refining Axial Regionalisation Through 3D Morphometric Sampling Strategies on Elapid Snakes

**DOI:** 10.1002/jmor.70147

**Published:** 2026-08-03

**Authors:** Ammresh, Marco Camaiti, Jane Melville, Alistair Evans, Emma Sherratt

**Affiliations:** ^1^ School of Biological Sciences Monash University Melbourne Victoria Australia; ^2^ Sciences Department Museums Victoria Melbourne Victoria Australia; ^3^ Department of Life Sciences Natural History Museum London UK; ^4^ School of Biological Sciences Adelaide University Adelaide South Australia Australia

## Abstract

Axial elongation has evolved repeatedly in vertebrates, and previous research has revealed insights into the developmental and evolutionary mechanisms that shape this body plan. Snakes, with their elongated, functionally limbless bodies, offer an exceptional system to investigate how vertebral regions are organised or modified throughout elongation. Recent work suggests that snakes retain ancestral regionalisation and that heart position coincides with the boundary between two regions, but how methodological choices such as vertebral sampling density and landmark dimensionality affect the detectability of these regions remain untested.

Here, we used three‐dimensional geometric morphometrics (3D GM) and segmented linear regressions on complete vertebral columns from 12 elapid snakes to investigate regionalisation and interspecific differences. We compared multiple subsampling strategies (every vertebra, or sampling every 2%, 2.5%, 4% or 5%) to assess how resolution can influence the detection of regional boundaries. We also tested various landmarking schemes, examining if previously utilised landmarking schemes differ greatly in region detection. Our analyses reveal a 4‐ or 5‐region model to be most suitable among elapids, including a short cervical region spanning 2%–4% of the column, a robust morphological shift at ~20% of the column aligning strongly with heart position and a distinct lumbar boundary in the final 5%. Interspecific shape differences were detected but lacked sufficient distinction to identify reliably. Fine‐scale sampling improves the detection of small regions, decaying with coarser subsampling. Our results determined that the coarsest reliable sampling method was found at the 2.5% mark.

These findings show that despite axial elongation, snakes retained a short cervical and lumbar region, with most of the elongation happening in the thoracic region. This, in turn, has resulted in the thoracic region undergoing axial repatterning, with three modules detected within the thoracic region. This study refines our understanding of vertebral modularity and highlights how regionalisation evolves in elongated vertebrates.

## Graphical abstract

Using 3D geometric morphometrics on every precaudal vertebra from 12 elapid snakes, this study used segmented linear regression to test regionalisation. The results show a five‐region model is best supported, including short cervical and lumbar regions and a strong heart‐aligned boundary at approximately 20% of the pre‐caudal column. Fine‐scale sampling recovered smaller regions more reliably, while coarser sampling increasingly collapsed regional structure, with 2.5% sampling representing the coarsest reliable compromise.

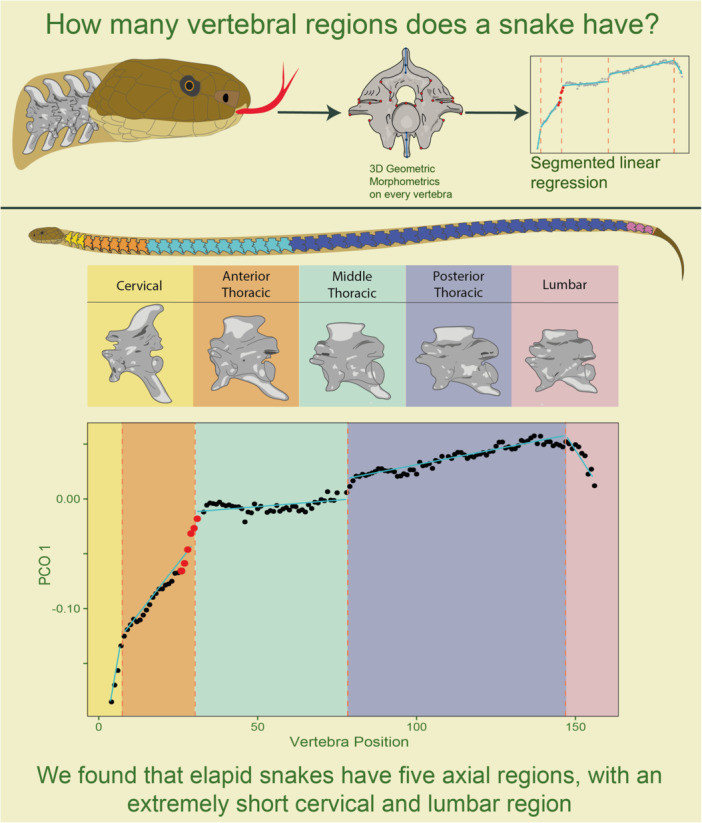

## 1 Introduction

The elongate vertebrate body form is one of the most striking examples of deviation from the basic tetrapod body plan, evolving independently multiple times across the vertebrate tree (e.g., eels, caecilians, skinks, geckoes, amphisbaenians, and snakes) in response to a wide range of ecological pressures and locomotory demands (Gans 1975; Lande 1978; Bejder and Hall 2002; Miralles et al. 2015; Camaiti et al. 2021). Elongate vertebrates share several recurring traits (Gans 1975; Woltering 2012): (i) the reduction and loss of limbs (at least one pair, often both), (ii) increased trunk length and flexibility via a multiplication of vertebrae along the vertebral axis, and (iii) apparent homogenisation of the vertebral column. These changes increase the benefit of undulatory locomotion in elongate vertebrates, underpinning a range of specialisations from fossoriality to aquatic propulsion. This study focuses on the third aspect, regionalisation, or the apparent lack thereof, in elongate vertebrates, and the methodology that is used to infer whether regions are present, and the boundaries between them.

The modern approaches to inferring regionalisation come from morphometrics of vertebral shape. Pioneered by Head and Polly (2015), they used two‐dimensional (2D) geometric morphometrics to characterise vertebral shape and graph profiles of intracolumnar morphological variation. The ordinated shape data are fitted to a segmented linear regression (SLR) to identify “breaks” along the profile that suggest a regional boundary. They showed that these breaks align with known Hox gene expression boundaries. Further advancements came with the understanding that the position of the heart also corresponds with one of these regional boundaries. Initially described by Malnate (1972), subsequent studies have confirmed that the heart consistently coincides with a morphological transition between vertebrae (Hampton 2019; Sherratt et al. 2019, 2022; Sherratt and Sanders 2020; Hampton et al. 2022; Sherratt et al. 2022; Nash‐Hahn et al. 2024; Hampton and Meik 2023). The SLR approach has been developed further, with Gillet et al.'s (2024) study on regionalisation in cetaceans as the most current body of work for SLR. This study primarily utilises the “MorphoRegions” package which was developed in that paper to analyse SLR.

The current limitation is that elongate vertebrates have elevated numbers of vertebrae, resulting in some researchers relying on coarse sampling strategies, a practical necessity given the logistical and time constraints of acquiring whole‐column datasets across multiple specimens. Landmark‐based studies of vertebral shape of elongate forms have typically sampled vertebrae at 5% intervals, for a total of 21 vertebrae per column (Head and Polly 2015; Hampton et al. 2022; Hampton and Meik 2023). While efficient, this method may be unable to detect small regions from subtle morphological changes (Hampton and Meik 2023). Other studies utilise even lower sampling to expand taxonomic coverage (Lowie et al. 2022), or sample almost every other vertebra while focusing on individual species (Sarris et al. 2012). Several studies exclusively used 2D landmarks in frontal view (Head and Polly 2015; Hampton et al. 2022; Hampton and Meik 2023), or lateral view (Sarris et al. 2012), possibly ignoring key three‐dimensional variation. A denser sampling method, while more time‐intensive, may increase the resolution of morphological gradients between regions. We hypothesise that increasing sampling resolution will improve the detection of subtle vertebral boundaries and stabilise breakpoint placement relative to traditional 5% interval sampling.

We use snakes as a study system because they have a long history of research on the evolution and development of their elongate body plan (Bellairs and Underwood 1951; Gans 1962; Woltering 2012). Originating from limbed lizard‐like ancestors, snakes first appear in the fossil record in the Middle Jurassic to Lower Cretaceous (ten Donkelaar 1998; LaDuke et al. 2010; Caldwell et al. 2015). They exemplify all three of Gans (1975) criteria: limb reduction/loss, exceptionally high number of vertebrae (up to 600 in some taxa), and were historically presumed to have homogenous axial columns (Caldwell 2003; Szyndlar and Georgalis 2023). The snake family Elapidae is a promising group for studying vertebral regionalisation. They are a relatively young lineage, originating in West Asia in the Late Eocene (~40 Ma) and rapidly diversifying across continents before reaching Australia around 25 Ma (Sanders et al. 2013; Lee et al. 2016; Sherratt et al. 2025), where they comprise more than 60% of snake fauna and occupy a variety of niches (Wilson and Swan 2021). Several studies have examined the axial skeleton of elapids. Smith (1975) study on four elapid species identified some diagnostic differences in vertebral shape, suggesting taxonomic use. Head and Polly (2015) identified four pre‐caudal regions in the two elapid species included in their dataset, showing that elapids have similar regional patterns to other snakes. Sherratt and Sanders (2020) found that heart position coincides strongly with a shift in centrum length and Sherratt et al. (2022) linked regionalisation to body shape diversification across elapids.

Early research described the pre‐caudal column as a largely uniform module, classifying it as a set of undifferentiated trunk vertebrae (Romer 1956; Hoffstetter and Gasc 1969; LaDuke 1991). Cohn and Tickle (1999) later suggested that axial elongation was achieved via altering Hox gene expression, with much of the trunk sharing a similar vertebral identity. Subsequent work demonstrated that snakes retain axial patterning despite subtle external morphological differentiation (Woltering et al. 2009; Woltering 2012). This more traditional axial patterning had its proponents (Sood 1941, 1948; Bullock and Tanner 1966; Szyndlar 1984; Carmona et al. 2010), but the small, incremental morphological changes from one vertebra to the next and the lack of demarcations posed substantial challenges for defining boundaries. Scanlon (2004) argued that snakes retain a cervical region, although the boundary is evolutionarily diffused, with cervical‐associated features extending posteriorly into the trunk. Tsuihiji et al. (2012) showed that the anterior musculature of snakes resembles the cervical musculature of other squamates, suggesting the persistence of a short but distinct cervical region. More recent research has defined the boundaries and regions within the thoracic column in snakes (Head and Polly 2015; Hampton 2019; Sherratt et al. 2019, 2022; Sherratt and Sanders 2020; Hampton et al. 2022; Sherratt et al. 2022; Nash‐Hahn et al. 2024; Hampton and Meik 2023). While these studies have demonstrated the presence of distinct regions in snake columns, statistical assessments of the number of such regions in the level of detail of this paper have yet to be conducted.

Here, we aim to address this gap by assessing the aforementioned methodological limitations. We also assess different vertebra sampling strategies to provide a solid methodological basis for characterising intracolumnar profile variation and regionalisation and to optimise data collection effort for further studies of axial regionalisation. To do this we sampled every precloacal vertebra in 12 individual snakes from three species using 3D geometric morphometrics to characterise the shape of the vertebrae. We applied SLRs to the intracolumnar shape profiles to estimate the number and position of distinct morphological regions. Using this dataset, we evaluated whether dense sampling of vertebrae (i.e., every vertebra) improves the detection of small or previously unresolved regions. We then investigate how sampling density (i.e., number of vertebrae) affects the detection of vertebral regions using the SLR method on multiple sampling resolutions (every vertebra, 2%, 2.5%, 4% and 5% intervals). We also test different landmarking protocols, comparing different parts of each vertebra and their impact on vertebral shape identity. Finally, we perform a preliminary analysis on shape differences within and among the three elapid genera used in this study, assessing how much species differ in their vertebral morphology. By integrating fine‐scale sampling with 3D shape analysis, this study provides new insights into how elongation has influenced vertebral regions in snakes.

## 2 Materials and Methods

### 2.1 Sample Preparation

We sampled 12 specimens across three terrestrial elapid species: six tiger snakes (*Notechis scutatus*), four eastern brown snakes (*Pseudonaja textilis*), and two lowlands copperheads (*Austrelaps superbus*), chosen based on availability (Supporting Information S1: Table S1). Deceased specimens were sourced from the Melbourne Museum and veterinary centres. Measurements of total length (mm) and snout‐vent length (SVL) (mm) were taken before dissection. The position of the heart in relation to the ventral scales was recorded during dissection and matched to the vertebral position. Ventral scale counts exhibit a well‐established, near 1:1 correspondence with vertebrae in elapid snakes, providing a robust proxy for axial segmentation (Alexander and Gans 1966; Shine 2000). This relationship has been utilised in heart position studies, supporting their continued use in comparative vertebral research (Sherratt et al. 2022; Hampton and Meik 2023). Skin and internal organs were removed by dissection before necrophagous dermestid beetles (*Dermestes maculatus*) were used to skeletonise each specimen. Prior to full skeletonization, a fishing line was strung through the neural canal of each snake to preserve vertebra order. Maceration was then used to completely remove any remaining non‐osteological material. Finally, each vertebra was disarticulated and separated by tape prior to scanning, in order to expedite segmentation and surface mesh generation.

### 2.2 Scanning and Generating 3D Models

Micro‐Computed Tomography (micro‐CT) scanning was done at the University of Melbourne, Victoria, Australia. Scans were performed with a Phoenix Nanotom M (Waygate Technologies) operated using XS Control and Phoenix Datos|x acquisition software. Coils of separated snake vertebrae were padded with bubble wrap and mounted in PVC pipes of 9 cm internal diameter. Scans were conducted collecting 1799 projections through a full 360‐degree rotation of the specimens, using the multiscan feature to stitch together 2–3 scans where samples spanned a greater height than the field of view of a single scan. An X‐ray tube voltage of 70 kV and a current of 300 mA was used. Resolution was optimised between 30 and 50 μm depending upon the width of sample coils (Supporting Information S1: Table S2). Volume reconstruction of the micro‐CT data was performed using the Phoenix Datos|x reconstruction software, applying an inline median filter, ROI‐CT filter and beam hardening correction filter during reconstruction. Reconstructed image stacks were imported into Avizo 3D v2023.2 (Thermo Fisher Scientific), where surface meshes were generated sequentially. Surface meshes were then exported in Polygon File Format (.ply).

### 2.3 Landmarking and Statistical Analysis

**Figure 1:**
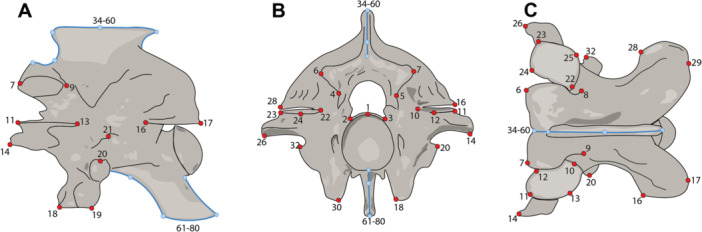
Illustration of an elapid vertebra showing the landmarking scheme. (A) Right lateral view of the vertebra; (B) anterior view; (C) dorsal view. Blue lines represent semi‐landmark curves. Supporting Information S1: Table S4 describes detailed locations of each landmark.

Surface meshes of individual vertebrae were imported into Stratovan Checkpoint v2025.05.23.09:27 (Stratovan Corporation 2025) for 3D landmarking. Landmarking began at the third vertebra, as the atlas and axis exhibit highly specialised morphologies compared to typical trunk vertebrae and therefore cannot be reliably landmarked using the same configuration. A configuration of 33 fixed anatomical landmarks and 47 sliding semilandmarks were placed on homologous points capturing variation (Figure 1). Descriptions of vertebral features and landmark positions are provided in the supplementary files (Supporting Information S1: Figure S1, Tables S3 and S4). Semilandmark curves were placed on the neural arch and hypapophysis (Bookstein 1997; Gunz et al. 2005). Landmark configurations were exported in Morphologika format (.txt), and all analyses were performed in the R Statistical Environment v2025.05.1 (R Core Team 2025). The main packages used were “MorphoRegions v0.1.0” (Gillet et al. 2024), “geomorph v4.0.10” (Baken et al. 2021; Adams et al. 2024) and “vegan v2.6‐4” (Oksanen et al. 2025). The notation of *package::function* will be used to specify the method we used for each analysis.

The landmark configurations of all vertebrae were subjected to a generalised procrustes analysis (GPA), which rotates, translates and scales specimens, removing non‐shape variation (Rohlf and Slice 1990). During superimposition, sliding semilandmarks were allowed to move tangentially along their respective curves to minimise bending energy. This was implemented in *geomorph::gpagen*.

### 2.4 Modelling of Axial Regions

Serial shape change along the vertebral column was quantified using SLR, in which principal coordinate (PCO) scores obtained from a principal coordinates analysis (PCoA) were modelled as response variables in a piecewise linear model, with vertebral position as the predictor. PCO scores were used instead of principal components analysis (PCA) scores as the MorphoRegions SLR function was not compatible with PCA scores. Procrustes‐aligned coordinates were converted into 2D matrices indexed by vertebral number. Then distance matrices were computed using Euclidean distances, and PCO scores were obtained using *MorphoRegions::svdPCO*. PCO axes explaining at least 95% of cumulative variance were retained using *MorphoRegions::PCOselect* and implemented in subsequent segmented regression analyses. Missing vertebrae (vertebrae too damaged to be landmarked) were inserted as NA rows and interpolated using the function *MorphoRegions::process_measurements*.

For each specimen, we fit candidate SLR models containing up to six regions with a minimum of two vertebrae per region. Six regions was the maximum number of regions that could be studied due to computational demands. An additional SLR model was run for each snake testing every other vertebra, up to eight regions. Breakpoints were estimated using *MorphoRegions::calcregions* and ranked using corrected Akaike Information Criterion (AICc) with *MorphoRegions::modelselect* and *MorphoRegions::modelsupport* (Akaike 2011). Breakpoint uncertainty was evaluated using *MorphoRegions::calcBPvar*, which computes weighted means (wMean) and weighted standard deviations (wSD) of breakpoint positions across supported models. Lower wSD values indicate sharper, more stable boundaries. The heart position of each snake was overlaid onto segmented regression plots as red points, enabling direct visual comparison between a boundary and the anatomical location of the heart. This relationship was also quantified with the midpoint of the heart against the second breakpoint in an ordinary least squares regression.

### 2.5 Vertebrae Subsampling Along the Axial Column

We evaluated how the proportion of pre‐cloacal vertebrae sampled along an axial column (sampling resolution) influenced inferred regionalisation by repeating the segmented regression pipeline above on evenly‐spaced subsets of vertebrae. For each snake, four subsampling schemes were generated to approximate common interval‐based designs: 51 vertebrae (2%), 41 vertebrae (2.5%), 26 vertebrae (4%), and 21 vertebrae (5%). Subsamples were selected using equally spaced indices along the full series, with the inclusion of the first and last vertebrae. Each subsample was independently Procrustes‐aligned, reshaped into a position‐indexed shape matrix, and ordinated by *MorphoRegions::svdPCO*.

SLR was then re‐fit to each subsample using PCO1 as the response variable, with a minimum region size of two vertebrae and a fixed number of regions (five in this subsampling workflow). Models were estimated with *MorphoRegions::calcregions*, ranked using AICc via *MorphoRegions::modelselect*, and summarised with *MorphoRegions::modelsupport*. To visualise how inferred regional boundaries shifted with sampling intensity, vertebral maps were generated for each snake and sampling level using *MorphoRegions::plotvertmap*, and stacked into per‐snake panels. These maps depict the predicted region assignments along the vertebral axis under a specified supported model, enabling qualitative assessment of boundary stability across subsampling schemes.

### 2.6 Comparison of Landmark Configurations

To evaluate whether regionalisation patterns were sensitive to landmark selection, analyses were repeated using four alternative landmark configurations. These included: (i) the full three‐dimensional landmark configuration of this study, capturing overall vertebral morphology (landmarks 1–80); (ii) an anterior‐focused landmark scheme adapted from Head and Polly (2015), emphasising anterior morphology (landmarks 1–7, 11, 12, 14, 23, 24, 26, 34, 60); (iii) neural spine landmarks (landmarks 34–54); and (iv) hypapophyseal landmarks (landmarks 60–80). Each landmark scheme was applied independently to vertebrae from all specimens and the resulting shape trajectories were compared to assess whether shape trajectories and inferred axial trends were consistent across different anatomical feature sets.

### 2.7 Examining Interspecific Variation in Vertebral Shape

To visualise the shape variation of vertebrae among all specimens of all species, we performed PCA of the Procrustes‐aligned coordinates (Gower 1975; Wold et al. 1987; Abdi and Williams 2010). A subset of the landmarking scheme (landmarks 1:7, 11, 12, 14, 23, 24, 26, 34:80) was retained for all PC ordinations, as it retained coverage of major vertebral structures contributing to overall shape variation while excluding landmarks that contributed little additional variation. Initial testing of PCAs showed that combining all regions into a PCA resulted in the primary axis being dominated by morphological change along the vertebral profile. To remedy this, vertebrae were separated into their assigned axial regions based on the SLR results and each region underwent separate, independent Procrustes alignment to examine interspecific shape variation within comparable positional segments. Within each region, raw coordinates were aligned using *geomorph::gpagen* and principal components were computed using *geomorph::gm.prcomp*. PC plots were generated for PC1 versus PC2 and PC1 versus PC3. Grouping was visualised via colouring points by genus and 95% confidence ellipses using *vegan::ordiellipse*. For shape interpretation, *geomorph::PlotReftoTarget* was used to plot lollipop diagrams of the minimum to maximum of each PC. Representative meshes of each region were selected and warped to the regional mean shape, before being warped to each PC extreme using *geomorph::WarpRefMesh*. These warps were used as three‐dimensional visualisation of principal‐axis shape change beside each PC.

## 3 Results

Differences in vertebral number among taxa were apparent. *Pseudonaja textilis* averaged approximately 206 pre‐caudal vertebrae, *Notechis scutatus* around 175, and *Austrelaps superbus* had on average 157 vertebrae (Supporting Information S1: Table S1). Their SVLs followed this pattern with *Pseudonaja textilis* having an SVL of between 99 and 105 cm, *Notechis scutatus* being between 78 and 92 cm and *Austrelaps superbus* between 66 and 80 cm.

### 3.1 Regionalisation of the Vertebral Column

**Figure 2:**
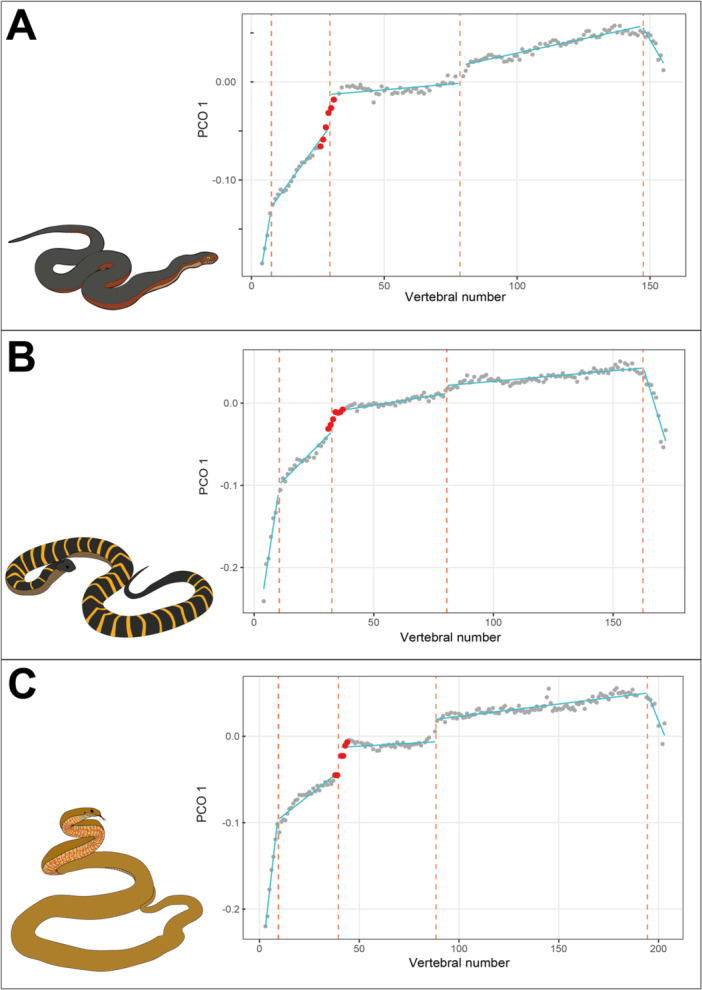
Segmented linear regression (SLR) fitted to PCO1 of Procrustes‐aligned 3D vertebral landmark data for three Australian elapid species: (A) *Austrelaps superbus_*MZRC 10088, (B) *Notechis scutatus*_NMV_D76366 and (C) *Pseudonaja textilis*_NMV_D76367. Dashed lines denote model‐estimated breakpoints and red points mark vertebrae that overlay the heart position. Competing SLR models with alternative breakpoint configurations were ranked, and the best‐supported AICc model with consistent breakpoints were chosen.

SLR of PCO1 of Procrustes‐aligned 3D vertebral landmarks of every pre‐cloacal vertebra identified the 6‐region model as the most statistically supported with the lowest AICc values (Supporting Information S1: Table S5). However, a 5‐region model was chosen as more suitable as the best‐supported model, as the 6‐region model had inconsistent breakpoint placements across the 12 snakes, while the 5‐region model had breakpoints with less variability (Supporting Information S1: Tables S5 and S6). The cervical region spans the anterior 3%–4% of the column and exhibits the steepest gradient in shape scores, indicating a high rate of morphological change from one vertebra to the next (Figure 2). The anterior thoracic region spans the next ~14%–16%, showing a low initial slope followed by a marked increase in slope approaching the boundary. Across specimens, this boundary strongly correlates with vertebrae overlapping the heart position (Supporting Information S1: Figure S2). The heart occupies the position of 17%–23% of the pre‐caudal length (Supporting Information S1: Table S1), and the second breakpoint occurs at around 16%–20% of the pre‐caudal length, indicating high positional correspondence. The middle thoracic region spans approximately 27% of the pre‐caudal length, with minimal variation in shape scores. The posterior thoracic region is the longest region in all snakes, spanning around 50% of the pre‐caudal length and showing a shallow gradient. Finally, the lumbar region spans the terminal ~3%–4.5% and exhibits a steep gradient, indicating a high rate of morphological change in a short region, similar to the cervical region.

### 3.2 Common Morphological Changes Along the Vertebral Column

**Figure 3:**
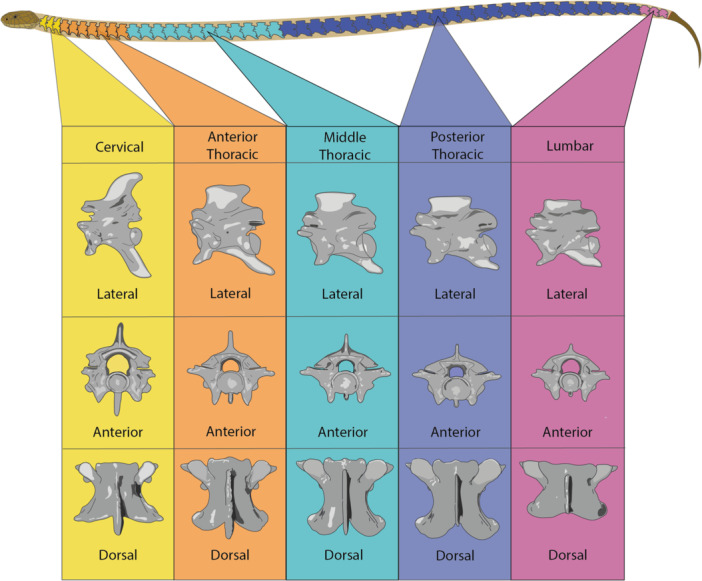
Mean vertebral shape for a tiger snake (*Notechis scutatus*_NMV_D76365), shown in lateral view (top row), anterior view (middle row) and dorsal view (bottom row). Region assignments derive from segmented linear regression on PCO1 scores calculated from Euclidean distances of Procrustes‐aligned coordinate data. A per‐region mean landmark configuration was calculated, and a reference mesh was deformed using *geomorph::WarpRefmesh* to generate mean shape meshes. Views were selected to capture centrum, neural arch, and hypapophyseal variation along the column.

Changes in vertebral morphology formed a consistent anterior‐to‐posterior gradient across all three species (Figure 2). Five regions were identified from the SLR model, which, in order of appearance, we designate as the cervical, anterior thoracic, the newly coined middle thoracic, posterior thoracic, and the lumbar regions (Figure 3).

### 3.3 Cervical Region

In all specimens analysed, a distinct cervical region was recovered, spanning between 4 and 10 vertebrae (Supporting Information S1: Tables S5 and S6). Although short in relative length, there are pronounced morphological changes from one vertebra to the next, shifting rapidly from the cervical to the thoracic form. Cervical vertebrae are characterised by relatively short centra, and tall neural spines that have a distinct “dolphin fin” like shape, differing from the rectangular thoracic neural spine (Figure 3). The hypapophyses are long and project nearly vertically in the ventral direction, with their length and angle relative to the condyle decreasing as we progress down the column. The zygapophyses are angled parallel to the centrum, distinct from the perpendicular zygapophyses in thoracic vertebrae.

### 3.4 Anterior Thoracic Region and Its Association With the Heart Position

Following the cervical region, the anterior thoracic region extends to approximately 20% of the pre‐caudal position across the snakes studied (Supporting Information S1: Table S7). This region is characterised by a shift in rate of morphological change; a marked decrease from the cervical region, until the boundary, where there is a sharp inflection in morphological change towards the middle thoracic (Figure 2). Anterior thoracic vertebrae show a flattening of the neural spine, maintaining its dorso‐ventral extension, but becoming more rectangular in shape (Figure 3). Hypapophyses are reduced in length and their angle relative to the centrum decreases, resulting in their tip extending past the condyle. The zygapophyses shift from a near‐parallel orientation to around 45° relative to the centrum, and the prezygapophyseal process lengthens laterally as well. All of the second breakpoints inferred consistently coincide with heart position measured, denoted by red dots (Figure 2, Supporting Information S1: Figures S2–S9). SLR models retrieve a strong and significant correlation between the anterior thoracic boundary and heart position (slope = 0.95, *p* = 0.001) (Supporting Information S1: Table S8).

### 3.5 Middle Thoracic Region

The middle thoracic region follows the anterior thoracic, occupying a quarter of the pre‐caudal length, extending from ~20% to ~46% in the snakes measured (Supporting Information S1: Table S7). Within the SLRs of *Austrelaps superbus* and *Pseudonaja textilis*, there is a clear depression in the vertebral profile immediately following the anterior thoracic boundary, a plateau followed by a gradual increase again (Figure 2). In contrast, *Notechis scutatus* exhibited a more gradual shape gradient, which resulted in a slightly higher variability in breakpoint placement and reduced support for a distinct middle thoracic region (Supporting Information S1: Table S6).

### 3.6 Posterior Thoracic Region

The posterior thoracic region represents the largest portion of the pre‐caudal vertebral column, spanning roughly half of the pre‐caudal length across all snakes examined (extending from ~46% to ~96% of the pre‐caudal length) (Supporting Information S1: Table S7). Despite this, the morphological gradient is the lowest in this region, and vertebrae here undergo little morphological change throughout (Figure 2). The centrum widens, becoming thicker and the hypapophyses continue to reduce in size, flattening and becoming wider (Figure 3). The prezygapophyses are perpendicular just as in the middle thoracic vertebrae, but they rise at an angle, with the distal ends elevated in comparison to the centrum joining portion. We also detect a decrease in the distal extension of the prezygapophyseal process towards the posterior end of the column.

### 3.7 Lumbar Region

The lumbar region is the last region of the pre‐caudal vertebral column, spanning approximately the final 4% of pre‐caudal length across all snakes studied. As with the cervical region, this interval is characterised by rapid shape change, as the vertebrae sharply shift from trunk to caudal morphology. Vertebrae in this region exhibit a progressive reduction in overall size, with both the centrum and neural spine becoming shorter and narrower (Figure 3). Hypapophyses shorten and flatten ventrally, diminishing in size until disappearing entirely at the cloacal vertebra. Prezygapophyses and their processes also decrease in size toward the posterior end of the column, while the neural canal appears proportionally larger relative to the shrinking centrum.

### 3.8 Vertebrae Subsampling Analysis

**Figure 4:**
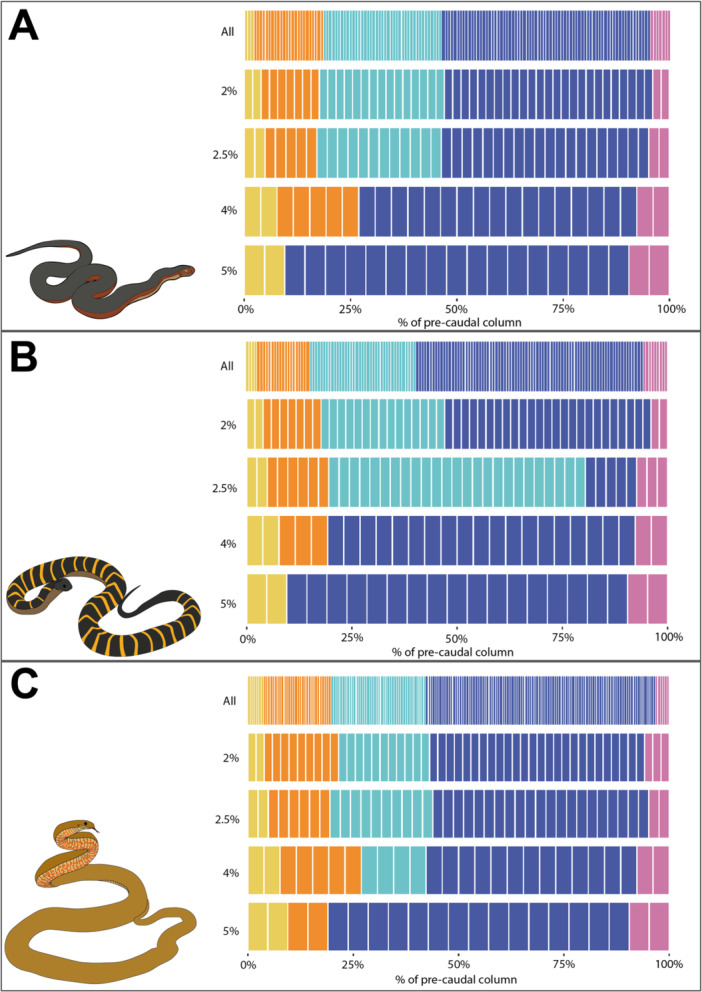
Vertebral maps for three Australian elapid species: (A) *Austrelaps superbus_*MZRC 10088, (B) *Notechis scutatus*_NMV_D76366, and (C) *Pseudonaja textilis*_NMV_D76367. Rows show the full column and subsamples at 2%, 2.5%, 4% and 5% vertebral intervals. Region boundaries for the subsamples were assigned from the top‐supported segmented regression model (AICc‐selected).

Subsampling analyses revealed that sampling density of vertebrae along the axial skeleton strongly influenced the detection and resolution of vertebral regions (Figure 4). Finer sampling intervals (all vertebrae, 2% and 2.5%) consistently recovered a five‐region model, whereas coarser sampling (4% and 5%) more often supported a three or four‐region model. At 2.5% sampling intervals, 10 of the 12 specimens recovered the same configuration as the full dataset. The two snakes that had breakpoint shifts were *Notechis scutatus*, where the displacement occurred at the middle thoracic boundary.

As sampling intervals became coarser, the inferred length of the cervical and lumbar regions increased, while the distinction between the middle and posterior thoracic regions weakened (Supporting Information S1: Tables S9 and S10). The cervical and lumbar regions comprised less than 5% in the full dataset, but expanded progressively as sampling became coarser. At the same time, thoracic breakpoints shifted across the column, although the anterior‐middle thoracic boundary remained relatively stable across all sampling strategies. This stability, occurring around 20% of pre‐caudal length and corresponding closely with heart position, showing that this major morphological change can be reliably detected at the 2.5% sampling density. At 5% sampling intervals, the middle thoracic region and, in some cases, the anterior thoracic region could not be recovered.

### 3.9 Comparison of Landmark Configurations

**Figure 5:**
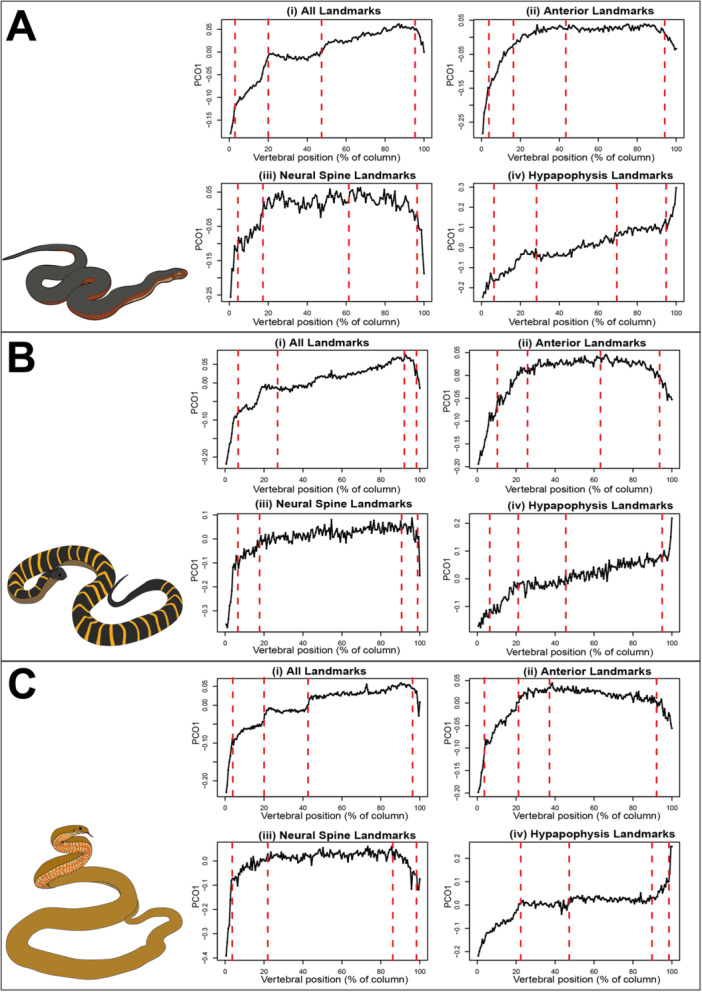
Four landmarking schemes for three Australian elapid species: (A) *Austrelaps superbus_*MZRC 10088, (B) *Notechis scutatus*_NMV_D76366, and (C) *Pseudonaja textilis*_NMV_D76367. For each snake (i) shows the full landmarking scheme, (ii) shows the anterior landmarks adapted from Head and Polly (2015), (iii) neural spine landmarks; and (iv) hypapophyseal landmarks.

Comparisons among the four landmark configurations indicate that regionalisation patterns are largely robust to landmark selection (Figure 5, Supporting Information S1: Figures S26–S34). Shape trajectories derived from the reduced landmark schemes (ii–iv) broadly mirror those obtained from the full 3D configuration (i), with major regional boundaries recovered in all analyses. However, the breakpoints in the alternative landmarking schemes are not consistent with the vertebral positions of the full landmarking scheme (Supporting Information S1: Table S11).

The anterior landmark configuration, adapted from Head and Polly (2015) and Hampton and Meik (2023), produced relatively smooth trajectories with steep gradients at the extreme ends of the column, closely resembling previously published research using 2D anterior landmarks (Hampton and Meik 2023). Neural spine landmarks reproduce the sharp morphological transitions present in the full landmark configuration but display high vertebra‐to‐vertebra variability, resulting in noisier trajectories and inconsistent breakpoint placements that obscure some regional boundaries. In contrast, hypapophysis landmarks closely match the steep gradients from the full landmarking scheme, particularly at cervical, anterior thoracic, and lumbar transitions, although the middle and posterior thoracic boundaries are inconsistent.

Subsampling analysis across all landmark configurations followed the same general trend observed in the full dataset, with coarser sampling recovering fewer regions than higher sampling (Figure 6, Supporting Information S1: Figures S35–S45) The anterior landmarking scheme seems to follow the full landmarking scheme the closest, while the Neural spine and Hypapophysis landmarking scheme substantially differ from the full scheme.

**Figure 6:**
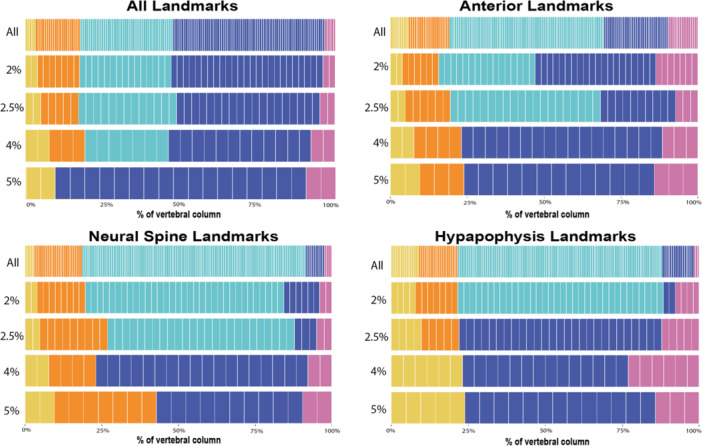
Subsampling of the four landmarking schemes for a snake *Austrelaps superbus* MZRC 10088. Each square represents a vertebra and each colour shows a distinct region and their size. The subsampling intervals are every 2%, 2.5%, 4% and 5% of the precloacal column.

### 3.10 Examining Interspecific Variation in Vertebral Shape

**Figure 7:**
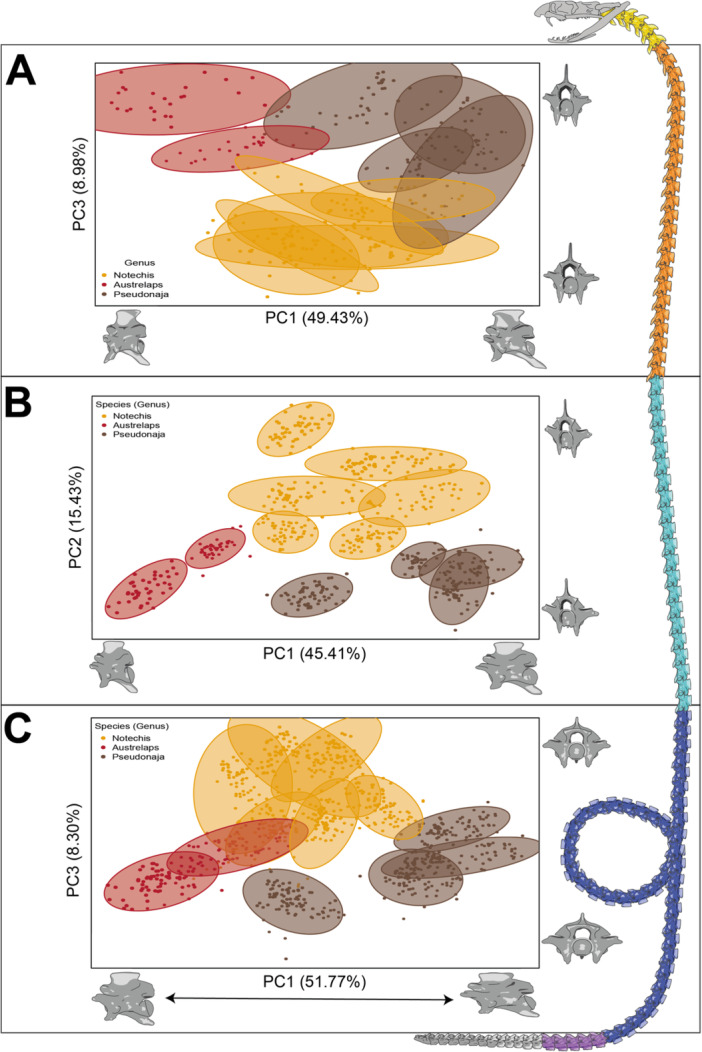
Regional PCA of the vertebral column based on generalised Procrustes‐aligned 3D landmarks. Panels show the PCs with the most separation between genera: (A) PC1 and PC3 for the anterior thoracic region, (B) PC1 and PC2 for the mid‐thoracic region, and (C) PC1 and PC3 for the posterior thoracic region. Reference meshes depict the same dataset warped to the shape extremes of each plotted PC using *geomorph::WarpRefmesh*. 95% Confidence ellipses of every vertebrae of an individual in that region.

PC analyses highlight differences among species within each thoracic region (Figure 7) with PC scores provided in Supporting Information S1: Table S12. In the anterior thoracic region, *Austrelaps superbus* vertebrae cluster towards the minimum area of PC1, marked by having anteroposteriorly shorter centra and ventrally projecting hypapophyses (Figure 7A). *Pseudonaja textilis* occupy the maximal ends, where they have longer centra and the hypapophyses extend far past the condyle. PC3 primarily reflects centrum proportions, where *Austrelaps superbus* and *Pseudonaja textilis* have mediolaterally narrower centra at the maximum, and *Notechis scutatus* has thicker anterior thoracic vertebrae at the minimum.

In the middle thoracic vertebrae, *Austrelaps superbus* again cluster towards the minimum of PC1, once again having shorter centra and taller neural spines (Figure 7B). *Pseudonaja textilis* are also at the maximum, with longer centra and shorter neural spines. PC2 separates *Notechis scutatus*, where they have a mediolaterally narrow centum relative to the other two species, contrasting its anterior thoracic placement.

The trend continues in the posterior thoracic with *Austrelaps superbus* clustered once again at the minimal end of PC1, characterised with smaller centra and tall neural spines, while *Pseudonaja textilis* at the maximal end has a longer centrum and more ventrally projecting hypapophyses. Within PC3, the minimal end is characterised by having wider centra, longer prezygapophyseal processes and larger zygosphenes. The maximal end, where *Notechis scutatus* primarily group, is characterised by having smaller centra, zygosphenes and shorter prezygapophyseal processes.

## 4 Discussion

This study aimed to provide a detailed, quantitative report on the axial skeleton of elapid snakes, as an exploration into elongation of vertebrates. We aimed to determine the number and placement of vertebral regions using 3D geometric morphometrics and SLR. We then evaluated how sampling density affects regional detection and how landmark protocols affect morphological gradients observed. In terms of the relationship between vertebrae and lengths, the pattern is consistent with pleomerism, in which increases in body length are associated with higher vertebral counts (Lindsey 1975). Although the strength of this relationship varies among snake clades, it has been documented in several lineages and supported by comparative analyses (Lindell 1994; Sherratt et al. 2019; Tiatragul et al. 2025). While this study only looked at three species, they reinforce the relevance of vertebral number as a major axis of interspecific variation and highlight the need for broader testing across multiple species of terrestrial elapids.

In terms of axial regionalisation, our results show that a five‐region model best explains regionalisation among the three snake species tested. These regions are herein defined as the cervical region, anterior thoracic, middle thoracic, posterior thoracic, and lumbar regions. The cervical region, which has been historically difficult to detect (Hampton and Meik 2023), was consistently identified with a full dataset sampling all pre‐cloacal vertebrae. Subsampling at coarser resolutions revealed that traditional 5% interval sampling (21 vertebrae) preferentially supported 3–4‐region models and inflated the relative size of short terminal regions. The lowest‐resolution subsampling scheme that retained stable boundary inference was 2.5% sampling (41 vertebrae). The full landmarking scheme was the most robust, but subsets still hold valuable morphological data and reflect column‐wide morphological integration.

Finally, interspecific differences in vertebral shape along the axial column were detected in PCA morphospaces. Although variation was detected, it was insufficient for reliable species level differentiation as intracolumnar axial variation exceeded interspecific variation, limiting discriminatory power for reliable species‐level classification and taxonomic research.

### 4.1 Regions, Boundaries and Gradients: Conceptual Clarity

This section clarifies the terminology used for SLRs throughout this study. Following Head and Polly (2015), a vertebral region is defined as a segment of the axial column characterised by a relatively consistent gradient or rate of morphological change. A boundary occurs where this gradient shifts, producing a breakpoint that separates adjacent regions.

The results indicate that vertebral regionalisation in snakes is best understood as a series of morphological gradients connected by transitional boundaries. Short regions, such as the cervical and lumbar regions, exhibit steep gradients and therefore rapid vertebra‐to‐vertebra shape change. In contrast, the middle and posterior thoracic regions display shallower gradients and more gradual morphological change. Within the thoracic regions, as vertebrae approach the next boundary, the rate of shape change often increases, akin to a step change towards the next morphological gradient.

Importantly, although SLR identifies regions as discrete segments, the underlying morphological variation remains continuous, albeit at different rates. Small‐scale shape variation is present throughout the vertebral column, ramping up near regional boundaries where changes in morphology become more pronounced. As a result of this SLR should be viewed as a useful approximation of large‐scale patterns of vertebral organisation rather than a complete representation of intracolumnar shape variation. Regionalisation in snakes is therefore neither entirely discrete nor entirely continuous, but reflects continuous morphological gradients organised into broader regions.

### 4.2 Regionalisation in Elapids

Elapid snakes have regionalised axial skeletons, yet the number of regions recovered here differs from previous research. Past research tested for and concluded that a 3–4‐region model was most suitable for a snake's pre‐caudal vertebral column. Johnson (1955) did not argue for discrete boundaries; instead, he described gradual morphological change in vertebral form along the column, recognising cervical, thoracic and sacral regions, with further subdivision of the thoracic region possible, but emphasised that most of the column forms a morphological continuum rather than sharply separated units. McCartney (2015) showed that some snake species show signs of clear morphological differentiation along the trunk. However, he did not formally partition the column into discrete regions, instead treating variation as a continuous gradient along the trunk. Head and Polly (2015) identified multiple regional domains along the pre‐caudal column, broadly corresponding to three to four morphological regions, and both elapid species included in their dataset exhibited four regions. Hampton and Meik (2023) likewise found that most sampled snake families exhibited four pre‐caudal regions (cervical, anterior thoracic, posterior thoracic and lumbar). Some species statistically recovered only three regions, largely due to difficulty identifying a cervical breakpoint, but anatomical interpretation supported four regions as the standard pattern. Hillan et al. (2024), analysing rib morphology rather than vertebrae directly, similarly concluded that snake taxa typically show either three or four regions within the pre‐caudal ribcage, while some limbed taxa showed best‐fit models with five or six regions.

In our data, we found that a 5‐region model is suitable in most scenarios when mapping the full elapid vertebral column (Figure 2, Supporting Information S1: Figures S2–S9). Several factors made a 5‐region model more suitable than other models. During the analysis, up to six regions were tested on every vertebrae and up to eight regions were tested on every other vertebrae (Supporting Information S1: Tables S5 and S13). Both criteria, the sum of residual squares and deltaAICc values, consistently chose the most complex models tested. Two scenarios were considered: (i) snakes have more than eight regions within their pre‐caudal vertebral column, and the true region count exceeds the number of regions tested, or (ii) the SLR model is more likely to select for the highest number of breakpoints once there is a high number of vertebrae (informal testing showed that the SLR model starts introducing additional breakpoints at around 50 vertebrae), even though some of the breakpoints may lack biological significance.

In the most complex models, breakpoints showed inconsistent placement across individuals, shifted neighbouring breakpoints, and lacked replicable anatomical correspondence. Because many of these breakpoints lacked clear morphological or biological correlates, scenario (ii) was better supported.

While we fully acknowledge that the most complex models are often best supported statistically (Supporting Information S1: Tables S5 and S13), a 5‐region model was deemed biologically relevant because its breakpoints showed consistent anatomical correspondence between individuals and interpretable shape change. These regions are described below.

#### 4.2.1 Cervical Region

The presence of a distinct cervical region within snakes has been contested, yet this separate gradient seen within the SLR supports the presence of a recognisable region. Further myological evidence corroborates this, with Tsuihiji et al. (2012) demonstrating that snakes retain craniovertebral musculature homologous to that of other squamates, indicating the presence of a cervical region rather than its complete loss. These cervical‐associated muscles terminate relatively early along the vertebral column, and phylogenetic optimisation of their posterior extents shows that the ancestral snake possessed only a modestly extended cervical region despite extreme overall body elongation. Hampton and Meik (2023) also hypothesised that this region existed, being too short to detect in their methodology of testing every 5%.

With osteological features, the unique element features would support the cervical as a unique module. The parallel zygapophyses have been hypothesised to be linked to an increase in torsion capacity, as Moon (1999, 2000) found that snakes use their heads and the anterior‐most portion of their body to fully encircle their prey during striking, thereby undergoing significant torsion. They also indicate that this serves as a functionally distinct anterior module that helps with manoeuvring. The slender vertebrae in this region may also facilitate tighter bends during prey capture and constriction.

An important acknowledgement is that many squamates possess cervical ribs, making rib absence alone an unreliable indicator of cervical identity. In some snakes, ribs occur as early as the first vertebra posterior to the atlas–axis complex, a condition also reported in other squamate lineages (Čerňanský et al. 2019). Across most squamate clades, the cervical region is therefore typically defined as the vertebrae anterior to the first rib that articulates with the sternum, most commonly comprising seven to eight vertebrae, although intraspecific deviations have been documented (Hoffstetter and Gasc 1969; Barbadillo and Barahona 1994). Detailed anatomical studies of varanids and lacertids further show that cervical vertebrae are characterised by reduced or modified ribs and distinct musculoskeletal associations related to head mobility rather than trunk function (Barbadillo and Barahona 1994; Cieri 2018). Using 3D geometric morphometrics of rib shape and SLR analysis, Hillan et al. (2024) identified a conserved anterior rib region in snakes characterised by rapid shape change toward thoracic morphology. This pattern closely mirrors the rapid vertebral shape change observed within this region in the present study. Taken together, these findings support the interpretation that snakes have retained an ancestrally short cervical region homologous to that of other squamates, with axial elongation occurring predominantly through expansion of the thoracic region.

#### 4.2.2 Anterior Thoracic Region and Its Association With the Heart Position

The second breakpoint lies directly before a plateau where the vertebrae undergo morphological change at a much slower pace. Quantitative analyses have demonstrated that heart position is developmentally anchored to this boundary (Hampton 2019; Sherratt et al. 2019; Hampton et al. 2022). Importantly, this boundary remains stable even in taxa with extreme modifications to the anterior body, such as sea snakes (Sherratt and Sanders 2020), and in arboreal colubrids (Nash‐Hahn et al. 2024), and across a multitude of diverse snake lineages throughout multiple transitions (Sherratt et al. 2022; Hampton and Meik 2023). The SLR results presented here further corroborate this pattern, with inferred breakpoints consistently coinciding with heart position across all specimens.

According to Malnate (1972), the hypapophysis functions as an attachment site for hypaxial trunk muscles, contributing to ventral stabilisation and bending of the column. In its absence, the muscles attach onto the centrum or haemal keel, which could result in some shifts in locomotive ability. Future studies might be able to test if this boundary is related to a shift in locomotion or behaviour, such as rearing up, striking at prey or dangling from a branch in open air. Additional evidence for the distinctiveness of the anterior thoracic region comes from hooding. Although the species examined here do not form the strict “hoods” of the genus *Naja*, all were capable of flaring the skin in the anterior body to appear larger. They can also rear up, exposing coloured ventral scales that could serve as a predator deterrent. In cobras, hooding is known to require specific modifications to ribs, vertebrae, and associated musculature, reinforcing that the anterior thorax functions as a morphologically and behaviourally specialised module (Young and Kardong 2010).

#### 4.2.3 Middle Thoracic Region

The middle thoracic region is perhaps the most contentious among the pre‐caudal divisions, being more apparent in some taxa than in others. Previous research generally describes snakes as having only three to four pre‐caudal regions (Head and Polly 2015; Hampton and Meik 2023), and even in our subsampling analyses, larger sampling intervals supported four regions as the best‐fitting model. The small breakpoint variability in *Austrelaps* and *Pseudonaja* but greater breakpoint variability in the *Notechis* snakes (Supporting Information S1: Table S6) suggests that while a five‐region organisation may be present in some taxa, others may exhibit partial fusion or weaker expression of this intermediate region. The prezygapophyses are almost perpendicular to the centrum, and the processes extend further out, indicative of a shift away from the highly torsion‐tolerant anterior morphology.

Recognition of a middle trunk region is well supported in palaeontological and comparative studies of snake vertebrae, where thoracic vertebrae are commonly subdivided into anterior, middle, and posterior regions based on naked‐eye observations of intracolumnar variation (LaDuke 1991; Polly et al. 2001; Pritchard et al. 2025; Węgrzyn et al. 2025). Historically, “mid‐trunk” vertebrae have been regarded as the least discretely diagnosable portion of the column and are often defined by their placement between stronger morphological boundaries rather than by their own unique morphological features (Szyndlar and Georgalis 2023). The recovery of this region in the present study using 3D geometric morphometrics of all pre‐caudal vertebrae and SLR analysis indicates that fine‐scale sampling and shape‐based analyses can pick up on subtle structures within this region.

#### 4.2.4 Posterior Thoracic Region

The posterior thoracic is characterised by being the longest and most morphologically stable region within a snake. The vertebrae within this region have the lowest rate of shape change, which is not unique to the snakes in this study, as mid and posterior trunk vertebrae have long been recognised as exhibiting gradual positional variation rather than sharply diagnostic characters, and are often identified relative to adjacent regions rather than by discrete anatomical markers (LaDuke 1991; Polly et al. 2001; Scanlon 1996; Szyndlar and Georgalis 2023). The shape change that we can see shows a continuation of the trend of a shorter, more rectangular neural spine. The length of this region could potentially extend the view that axial elongation in snakes primarily occurs through the expansion of the thoracic region (Cohn and Tickle 1999; Gomez and Pourquié 2009), with a majority of the expansion happening in the posterior thoracic. In this context, the posterior thoracic region is best interpreted as a stabilised trunk segment that accommodates elongation while maintaining biomechanical continuity along the axial column.

#### 4.2.5 Lumbar Region

The lumbar region is the last region of the pre‐caudal vertebral column, with the shape changes described here converging towards the shape of caudal vertebrae. Prezygapophyses and their processes decrease in size toward the posterior end of the column, while the neural canal appears proportionally larger relative to the shrinking centrum, possibly as it still needs to accommodate the spinal cord. Rib morphology changes markedly in this region: typical thoracic ribs are lost, and forked ribs fused to the centrum (lymphapophyses) appear (Szyndlar and Georgalis 2023). The cloacal vertebra lacks a hypapophysis, possibly to accommodate cloacal structures and functions, and is followed by caudal vertebrae bearing forked structures called haemapophyses. Most specimens, save for a couple, had only one cloacal vertebra, with two specimens having two cloacal vertebrae bearing no hypapophyses. Considerable variation exists among individuals where lymphapophyses can appear a few vertebrae anterior to the cloacal vertebra and in some cases a vertebra may have a thoracic rib on one side and a forked rib on the other. This variability, combined with the abrupt morphological shifts observed, support the interpretation of the lumbar region as a highly compressed but distinct transitional zone rather than a prolonged morphological module.

### 4.3 Vertebrae Subsampling Analysis

Our vertebrae subsampling analysis demonstrates that sampling density along the axial skeleton has a strong and predictable influence on inferred axial regionalisation. At higher sampling densities (every vertebra, 2%, and 2.5% interval sampling), five‐region models were frequently best supported (Figure 4, Supporting Information S1: Figures S10–S18). In the two snakes that did not recover five regions the displaced breakpoint corresponded to the weakly expressed middle thoracic boundary identified in the full dataset, consistent with a weaker middle thoracic region in *Notechis scutatus*.

At 5% sampling intervals (21 vertebrae) the best supported models shifted toward three and four‐region models, with the middle thoracic region and in some cases, the anterior thoracic region no longer being recovered. This outcome is consistent with the loss of subtle inflection points along continuous shape gradients at coarser sampling intervals. The occasional loss of the anterior thoracic boundary is notable, given its strong correlation to the heart position and its associated morphological shift (Malnate 1972; Hampton 2019; Sherratt et al. 2019; Hampton et al. 2022).

Most previous studies using segmented regression on snake axial columns have relied on this 5% sampling strategy (Head and Polly 2015; Hampton et al. 2022; Hampton and Meik 2023), which likely explains why finer‐scale regions were not consistently detected in earlier work. Notably, the cervical region was consistently recovered even at 5% sampling intervals in the present study. This contrasts with some previous analyses (Hampton and Meik 2023) and likely reflects differences in sampling where the explicit inclusion of the most‐anterior vertebrae likely improved detection of the cervical region.

### 4.4 Landmark Selection

The comparison of the four landmarking schemes showed that vertebral regionalisation patterns are broadly robust to landmark selection. All landmark configurations recovered the five‐region model as the most appropriate, although it is worth mentioning that five regions was the maximum regions tested and from the previous section we can surmise the function will always choose the most complex model as the most supported. Although the breakpoints varied among configurations, the large scale gradients looked similar, indicating that these elements all contribute to axial regionalisation, rather than any single anatomical feature alone (Figure 5, Supporting Information S1: Figures S26–S34 and Table S11). Figure 5 further illustrates that substantial shape changes occur between adjacent regions, involving coordinated changes in each of these elements. Together, these results suggest that vertebral regionalisation reflects integrated morphological change across the vertebra rather than isolated variation within individual elements.

Despite these overall similarities, important differences among landmark configurations were observed. The anterior landmark dataset most closely resembled the full three‐dimensional configuration, whereas the neural spine and hypapophyseal datasets showed greater variability in breakpoint placement among specimens. Breakpoints associated with steep morphological transitions, particularly within the cervical, anterior thoracic, and lumbar regions, remained comparatively stable across configurations, whereas middle and posterior thoracic boundaries were more variable. This suggests that some vertebral elements undergo weaker or more gradual shape transitions within these regions, reducing the consistency of inferred breakpoints.

Comparison with landmarking approaches used in previous studies further supports these interpretations. Anterior‐face landmark datasets (Head and Polly 2015; Hampton and Meik 2023) and centrum‐length analyses (e.g., Sherratt and Sanders 2020; Nash‐Hahn et al. 2024) consistently recover major anterior thoracic and lumbar transitions, but often under‐resolve cervical and middle thoracic structure. Similarly, the anterior‐view analyses in the present study produced smooth gradients at the anterior and posterior ends of the column but lacked some of the sharper inflections observed in the full landmark configuration, suggesting that anterior morphology captures broad axial gradients while underrepresenting finer‐scale transitions. In contrast, neural spine landmarks reproduced many sharp transitions present in the full dataset but exhibited high vertebra‐to‐vertebra variability, resulting in noisier regression trajectories that obscured some regional boundaries. Hypapophyseal landmarks most closely matched the full dataset at the cervical, anterior thoracic, and lumbar transitions, indicating that hypapophyseal morphology contributes strongly to regional discrimination, although increased variability was observed posteriorly where the hypapophysis becomes reduced or absent. These findings broadly align with Sarris et al. (2012), who found that intracolumnar variation in Daboia russelii was driven primarily by changes in neural spine and hypapophyseal morphology, whereas centrum shape remained comparatively conservative.

Collectively, these results indicate that characterising vertebral shape in two‐dimensions with a small number of landmarks still captures biologically meaningful aspects of axial variation and support many conclusions from previous studies using simplified landmarking strategies. However, three‐dimensional landmark configurations combined with semilandmarks for curves recover subtler morphological transitions and provide a more complete representation of axial organisation by integrating signal across multiple vertebral elements simultaneously.

Subsampling analyses further demonstrated that a 2.5% sampling interval provides the closest approximation to the full dataset while substantially reducing sampling effort. This pattern was most consistent in the full and anterior landmark configurations, whereas neural spine and hypapophyseal datasets remained comparatively variable. In some cases, reduced sampling density smoothed local vertebral variation and produced more stable breakpoint estimates. Overall, these results suggest that a 2.5% sampling strategy represents an effective compromise between sampling efficiency and regional resolution. Additionally, the strong correspondence between the anterior landmark dataset and the full three‐dimensional configuration suggests that anterior vertebral morphology alone captures a substantial proportion of axial shape variation along the vertebral column.

### 4.5 Interspecific Variation

We can make comparisons among species measured, but due to the low sample size these should be considered preliminary. In terms of vertebral number, we can see that the copperheads average the fewest at 154 precloacal vertebrae (Supporting Information S1: Table S1). Tiger snakes are next (174) and brown snakes have the most, averaging 203 precloacal vertebrae. This follows average snout vent lengths as well, copperheads (~70 cm), tiger snakes (~80 cm), eastern brown snakes (~95 cm), which raises the possibility of pleomerism (Lindsey 1975). While Lindell (1994) found pleomerism as a whole within snakes, subsequent studies have found them to vary within families (Shine 2000; Sherratt et al. 2019; Tiatragul et al. 2025). Sherratt et al. (2019) found no pleomerism in marine elapids and Shine (2000) paper found no correlation in eight species, but a comprehensive study has yet to be conducted.

For the average sizes of each of the regions, all snakes have extremely close region lengths (Supporting Information S1: Table S7). While the total vertebral counts within each region varied, the percentage lengths of all regions were within a couple percent of each other. This is probably due to them occupying similar ecologies of large terrestrial predators resulting in no major discernible difference in region sizes.

Interspecific differences in vertebral shape were subtle and insufficient to discriminate taxa visually. With such a small sample size, these results should be treated as exploratory and warrant further research. Despite this, some trends were apparent, including relatively anteroposteriorly shorter centra in *Austrelaps superbus* and mediolaterally narrower vertebrae in *Notechis scutatus*, but these differences were detectable only through three‐dimensional geometric morphometric analyses and are not readily distinguishable by eye. Interspecific variation was small relative to intracolumnar shape change, such that analyses combining all vertebral positions resulted in substantial overlap among taxa. Only after vertebrae were partitioned into axial regions did interspecific structure become more apparent in morphospace, and even then, regions undergoing rapid morphological change, such as the cervical and lumbar regions, showed little discernible separation among species (Supporting Information S1: Figures S20, S21, S25 and S26). These patterns suggest that positional variation along the column exerts a stronger influence on vertebral shape than species identity.

Comparable trends have been reported in other vertebral studies. Tisza et al. (2024) showed that viperid vertebrae recovered from raptor feeding remains could be assigned to species using geometric morphometrics and discriminant analyses despite minimal visual differences among taxa. Similarly, Jessee (2023) demonstrated that vertebral shape differences among viperid species are statistically detectable but remain small relative to positional variation along the column. In both cases, reliable classification required quantitative morphometric approaches rather than visual inspection, consistent with the patterns observed here. Earlier qualitative work on Australian elapids similarly noted limited visual diagnosability, with Smith (1975) describing *Austrelaps superbus* as having broader centra, *Pseudonaja textilis* as showing elongation of posterior trunk vertebrae, and *Notechis scutatus* as possessing comparatively slender centra, yet emphasising that naked‐eye distinction among taxa remained difficult. The substantial morphological overlap observed here is therefore unsurprising given the broadly similar ecologies of these species, all of which are large terrestrial predators occupying comparable trophic roles. Nevertheless, ecological differences may still contribute subtly to vertebral morphology, although this relationship was not directly tested here.

These findings further refine our understanding of how axial elongation interacts with vertebral regionalisation in snakes. Rather than resulting in a uniformly homogenised trunk, elongation in elapids retains distinct regional modules, with sharp gradients at key functional and developmental boundaries. This supports the view that snake axial elongation reflects lengthening of ancestral squamate regions rather than complete axial homogenisation.

### 4.6 Future Directions

This study demonstrates a full‐scale regionalisation analysis in snakes and explores methodological conventions used in similar studies. Several avenues can be explored and built upon this research. The interspecific variation shows fairly small differences in both shape and region sizes, which is to be expected from closely related snakes with similar ecologies. Expanding this study across a broader diversity of elapids would be useful in understanding if these regions do change and what factors might cause them to do so. Additionally, the three snakes show signs of positive pleomerism, contradicting previous research in elapids. A full scale study on terrestrial elapid pleomerism may be useful in determining if this family follows vipers and new world natricines in pleomerism (Shine 2000).

This study highlights an interesting pattern seen in vertebral shape gradients. Within longer regions, particularly the middle and posterior thoracic regions, vertebral morphology changes very little from one vertebra to the next, producing shallow morphological gradients. However, the rate of shape change often increases near regional boundaries before transitioning into the gradient of the next region. We suggest that axial elongation may occur through the expansion of existing regions, where additional vertebrae are added in and have the morphological identity in the region in which they develop. This might be an explanation for the shallow gradients while preserving the major transitions between regions, which could be retained from the ancestral squamate plan. The intracolumnar shape profiles of the lizard and snake in Head and Polly (2015) further highlight this. Both reptiles have a broadly similar vertebral shape profile, but the snake column exhibits clusters of morphologically similar vertebrae separated by more rapid transitions. This expansion of existing developmental domains, rather than uniform stretching of the vertebral column is compatible with the clock and wavefront model. In snakes, the segmentation clock operates faster relative to the rate of developmental growth than in other amniotes, producing greater numbers of somites (Gomez et al. 2008; Gomez and Pourquié 2009). If these additional somites are produced within existing Hox expression domains, they may retain the regional identity of the domain in which they form. This would expand particular axial regions while preserving major developmental boundaries, generating extended plateaus of similar vertebral morphology separated by sharper transitions.

This interpretation also fits with previous work suggesting that changes in Hox expression contributed to snake axial elongation and regional patterning (Cohn and Tickle 1999), while later studies indicate that developmental regionalisation can persist even when external vertebral differences are subtle (Woltering et al. 2009; Woltering 2012; Head and Polly 2015). Under this model, elongation would not erase regionalisation, but instead increase the number of vertebrae assigned to particular regional identities. Alternative mechanisms such as shifts in Hox boundary positions or uniform elongation of the vertebral column may also contribute to the patterns observed. Future studies are needed to determine how vertebral multiplication interacts with regional identity during the evolution of snake axial elongation.

## 5 Conclusion

This study demonstrates that vertebral regionalisation in elapid snakes is more complex than previously recognised, with five regions consistently recovered across the pre‐caudal axial skeleton. Short but distinct cervical and lumbar regions are ancestrally retained despite extreme axial elongation, while the anterior thoracic region shows a strong and repeatable correlation with heart position. In contrast, the middle and posterior thoracic regions form a morphologically conservative region spanning a large portion of the pre‐caudal axial skeleton. Regional boundaries are strongest at key developmental or functional boundaries, while being weaker across much of the trunk. These results indicate that axial elongation in snakes has not resulted in a loss of vertebral regionalisation, but rather ancestral regions have been retained and elongated.

Methodologically, our analyses show that vertebrae sampling density along the axial column and landmark selection strongly influence regionalisation inferences. Coarse 5% sampling intervals underestimate regional complexity, whereas denser sampling and three‐dimensional landmarking reliably recover smaller regions that would have otherwise been lost. These findings underscore the importance of integrating quantitative models with anatomical interpretation when assessing axial organisation.

Finally, interspecific differences in vertebral shape among elapids were weak relative to intracolumnar variation, suggesting that isolated vertebrae may be difficult to assign reliably to species without quantitative methods. Vertebral number, however, varied substantially among species. Broader taxonomic and ecological sampling will be necessary to determine how axial morphology varies across terrestrial elapids, and other elongate taxa more generally.

## Author Contributions

Ammresh designed and performed the research, analysed the data and wrote the paper. Marco Camaiti, Alistair Evans and Emma Sherratt helped with the study design, interpretation of data and reviewed drafts of the paper. Jane Melville provided funding and reviewed drafts of the paper.

## Ethics Statement

The authors have nothing to report.

## Conflicts of Interest

The authors declare no conflicts of interest.

## Supporting information


Supporting File

## Data Availability

The data that support the findings of this study are openly available in Figshare at https://doi.org/10.6084/m9.figshare.31828813, reference number 273097.
