# Supplementary material for: The Backbone of Regionalisation: Refining Axial Regionalisation Through 3D Morphometric Sampling Strategies on Elapid Snakes
Source: J Morphol. 2026 Aug 3;287(8):e70147. doi: 10.1002/jmor.70147 (PMC13433002; doi:10.1002/jmor.70147)
Supplement: Supplementary file 1 — Supporting File [file JMOR-287-e70147-s001.docx]

**Supplementary Section**


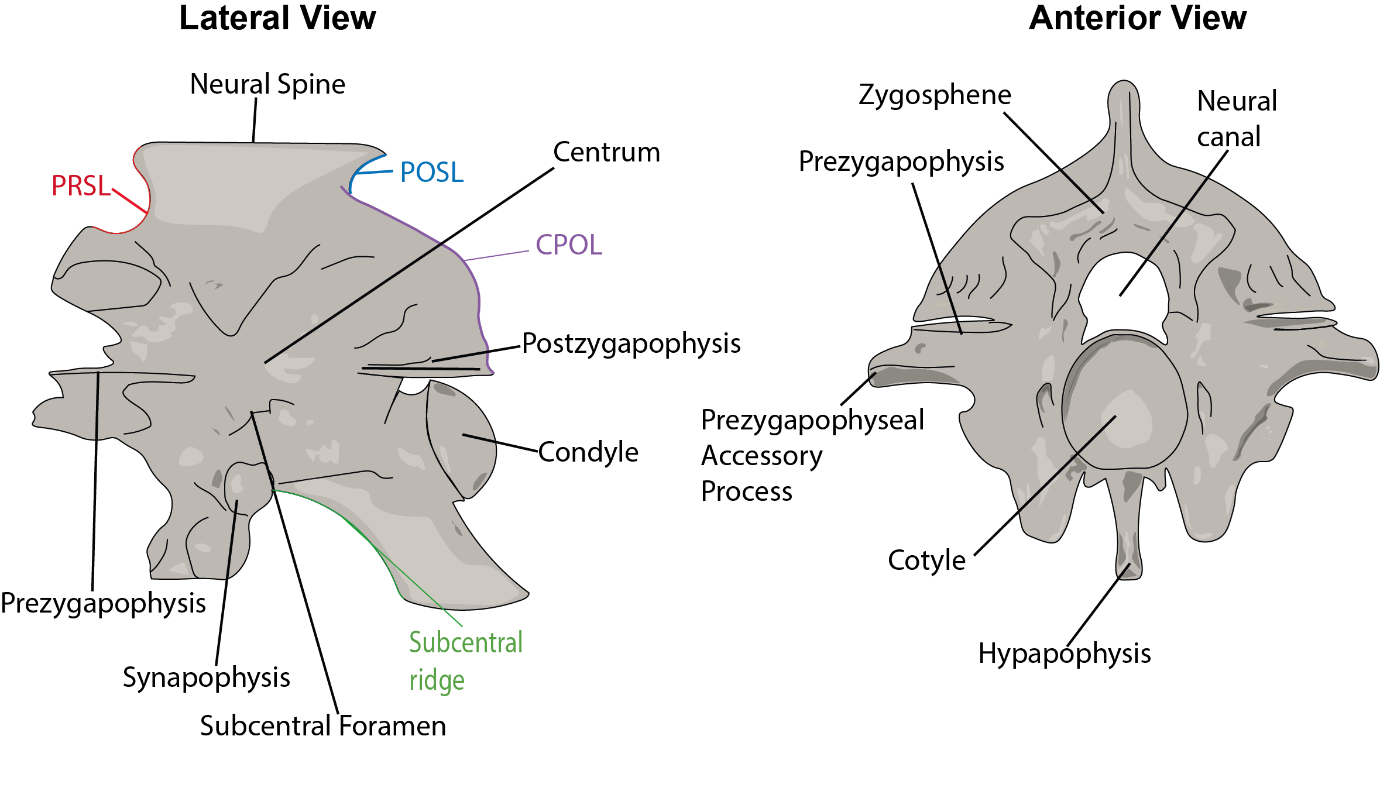


**Supplementary Figure 1**: Lateral view (left) and anterior view (right) of a typical snake vertebra. Features and processes are labelled and laminae and spines are highlighted in colour.


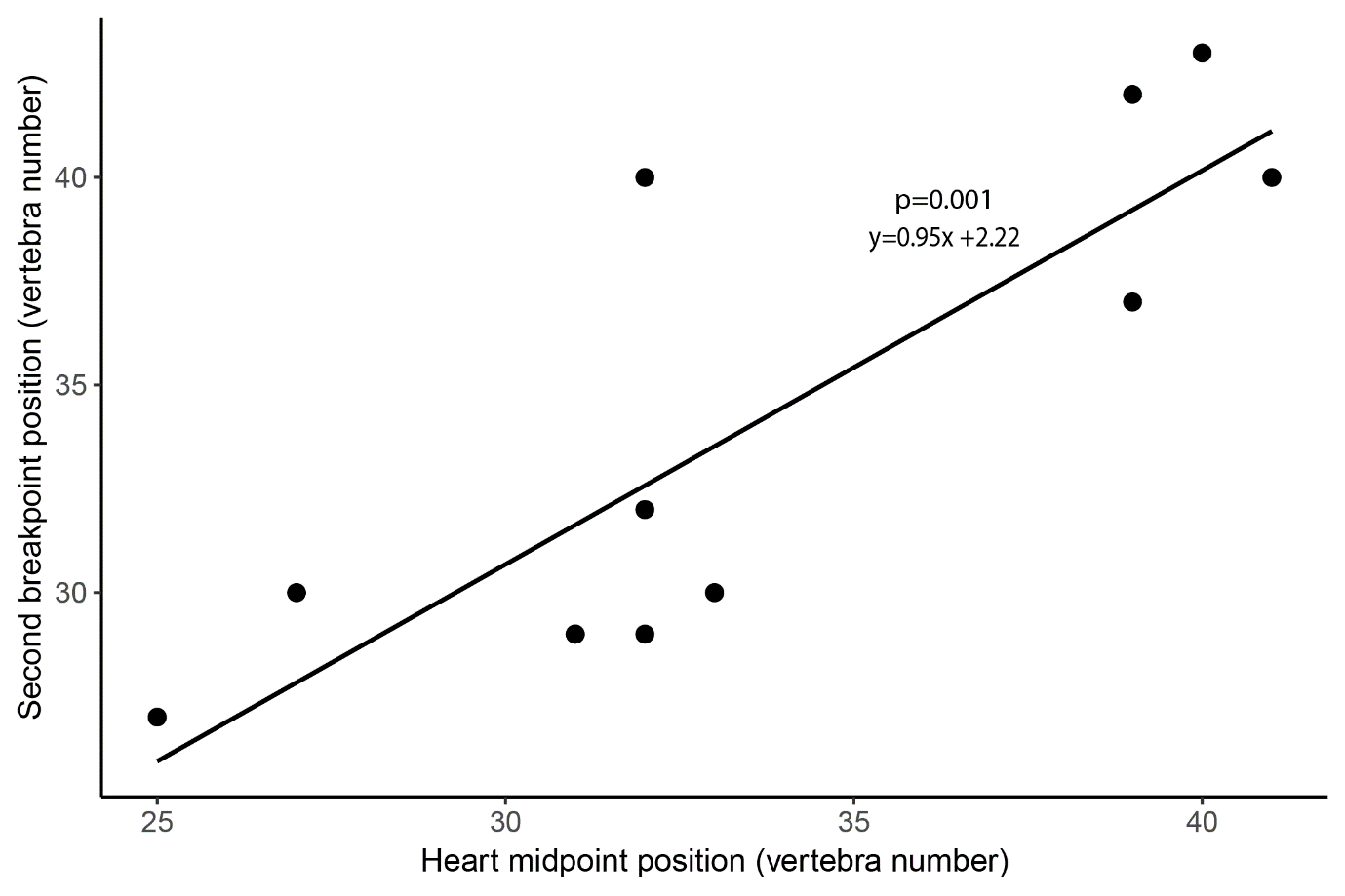


**Supplementary Figure 2**: Ordinary least squares regression of the heart midpoint position of each snake against the second breakpoint of the segmented linear regression. (β = 0.95 ± 0.20 SE, p = 0.001).

**
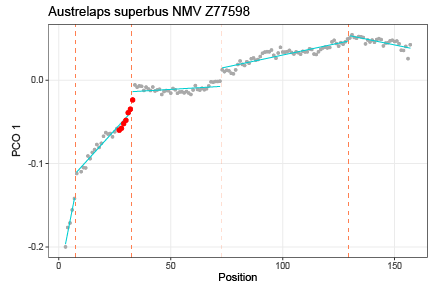

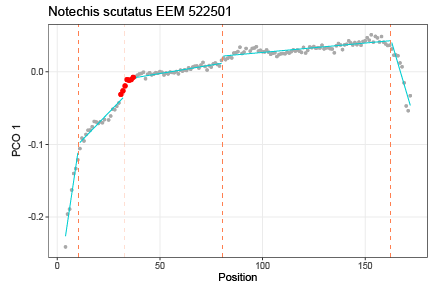
Supplementary Figure 2:** Figure showing the segmented linear regression (SLR) model of a lowlands copperhead (*Austrelaps superbus* NMV Z77598). The red points indicate the heart position along the vertebral column. The number and position of breakpoints were determined via Akaike Information Criterion (AICc).

**Supplementary Figure 3:** Figure showing the segmented linear regression (SLR) model of a tiger snake (*Notechis_scutatus_*MZRC_10089). The red points indicate the heart position along the vertebral column. The number and position of breakpoints were determined via Akaike Information Criterion (AIC).

**
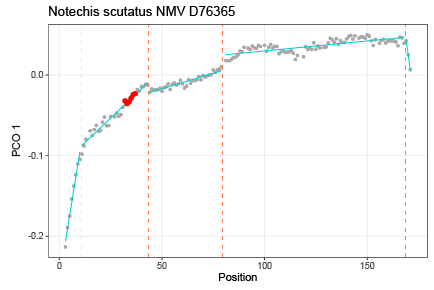
Supplementary Figure 4:** Figure showing the segmented linear regression (SLR) model of a tiger snake (*Notechis_scutatus_*MV_D76365). The red points indicate the heart position along the vertebral column. The number and position of breakpoints were determined via Akaike Information Criterion (AIC).


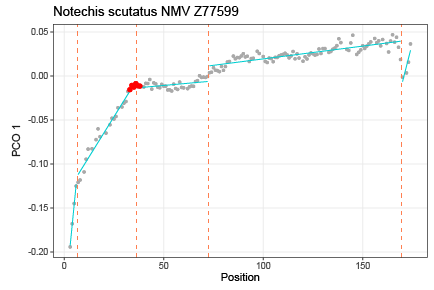


**Supplementary Figure 5:** Figure showing the segmented linear regression (SLR) model of a tiger snake (*Notechis_scutatus_*NMV_Z77599). The red points indicate the heart position along the vertebral column. The number and position of breakpoints were determined via Akaike Information Criterion (AIC).

**
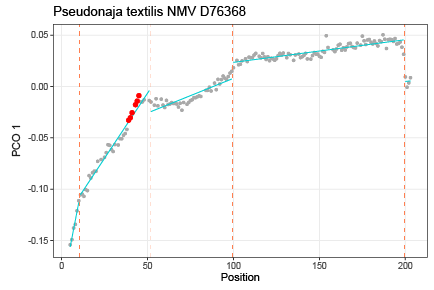

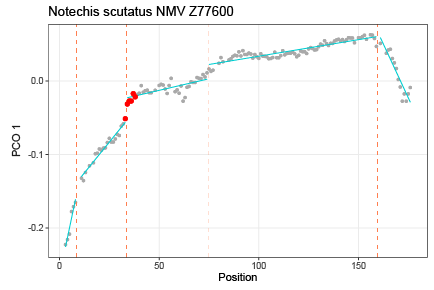
Supplementary Figure 6:** Figure showing the segmented linear regression (SLR) model of a tiger snake (*Notechis_scutatus_*MV_Z77600). The red points indicate the heart position along the vertebral column. The number and position of breakpoints were determined via Akaike Information Criterion (AIC).

**Supplementary Figure 7:** Figure showing the segmented linear regression (SLR) model of an eastern brown snake (*Pseudonaja_textilis_*MZRC_10093). The red points indicate the heart position along the vertebral column. The number and position of breakpoints were determined via Akaike Information Criterion (AIC).


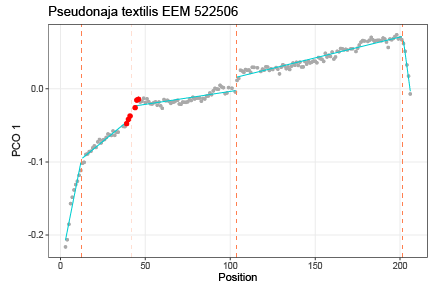
**Supplementary Figure 8:** Figure showing the segmented linear regression (SLR) model of an eastern brown snake (*Pseudonaja_textilis_*NMV_D76368). The red points indicate the heart position along the vertebral column. The number and position of breakpoints were determined via Akaike Information Criterion (AIC).


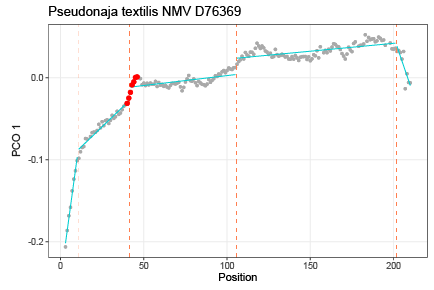


**Supplementary Figure 9:** Figure showing the segmented linear regression (SLR) model of an eastern brown snake (*Pseudonaja_textilis_*MV_D76369). The red points indicate the heart position along the vertebral column. The number and position of breakpoints were determined via Akaike Information Criterion (AIC).

**Part 2: Interspecific vertebral shape comparisons**


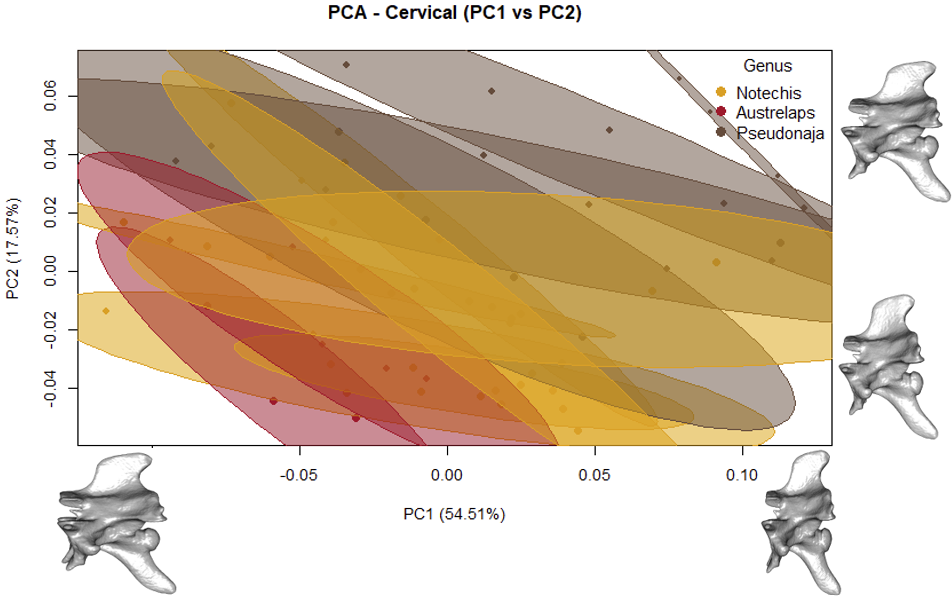


**
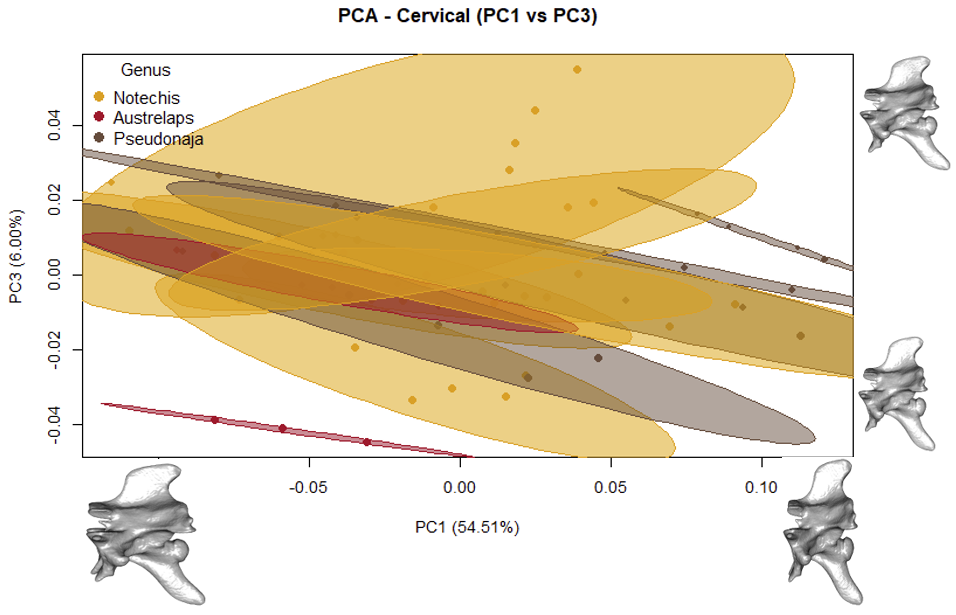
Supplementary Figure 10:** PCA plot with PC1 and PC2 of all vertebrae in the cervical region. The ellipses show the 95% confidence ellipse for each snake with red representing *Austrelaps superbus*, gold representing *Notechis scutatus* and brown presenting *Pseudonaja textilis*. PC1 represented 54.51% of the total variation in the dataset and PC2 represents 17.57% of the total variation.

**Supplementary Figure 11:** PCA plot with PC1 and PC3 of all vertebrae in the cervical region. The ellipses show the 95% confidence ellipse for each snake with red representing *Austrelaps superbus*, gold representing *Notechis scutatus* and brown presenting *Pseudonaja textilis*. PC1 represented 54.51% of the total variation in the dataset and PC3 represents 6% of the total variation.


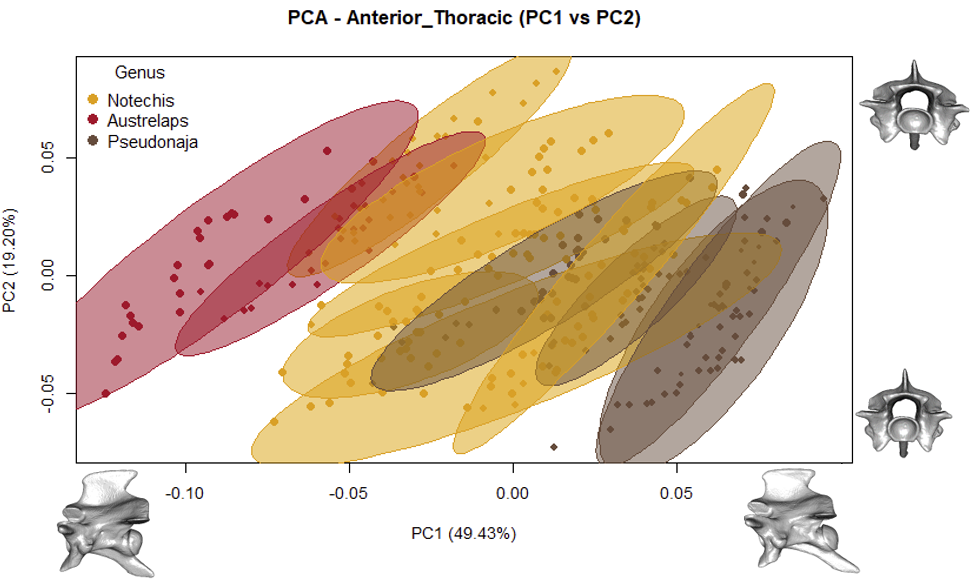


**Supplementary Figure 12:** PCA plot with PC1 and PC2 of all vertebrae in the anterior thoracic region. The ellipses show the 95% confidence ellipse for each snake with red representing *Austrelaps superbus*, gold representing *Notechis scutatus* and brown presenting *Pseudonaja textilis*. PC1 represented 49.43% of the total variation in the dataset and PC2 represents 19.20% of the total variation.


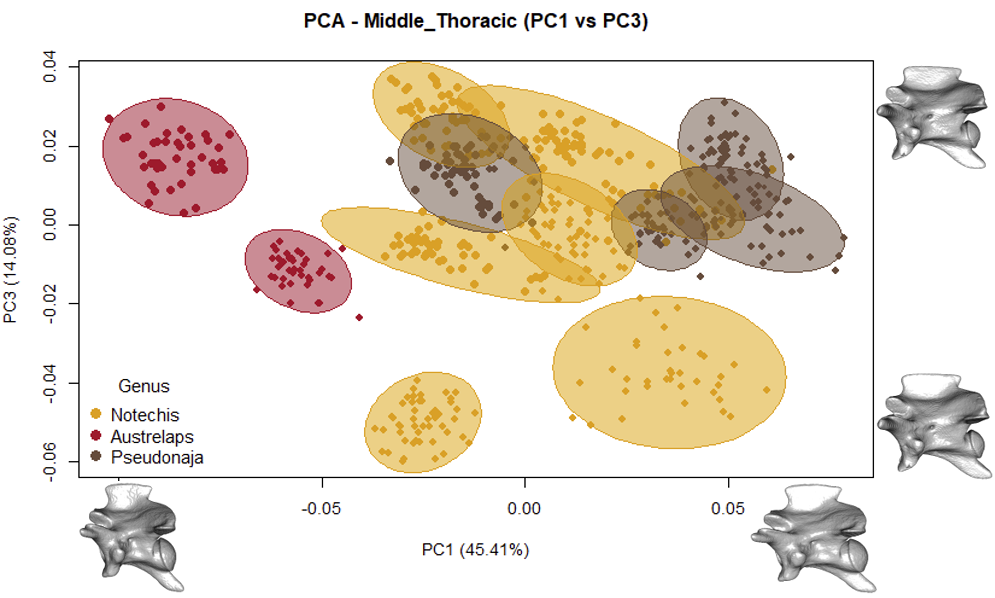


**Supplementary Figure 13:** PCA plot with PC1 and PC3 of all vertebrae in the middle thoracic region. The ellipses show the 95% confidence ellipse for each snake with red representing *Austrelaps superbus*, gold representing *Notechis scutatus* and brown presenting *Pseudonaja textilis*. PC1 represented 45.41% of the total variation in the dataset and PC3 represents 14.08% of the total variation.


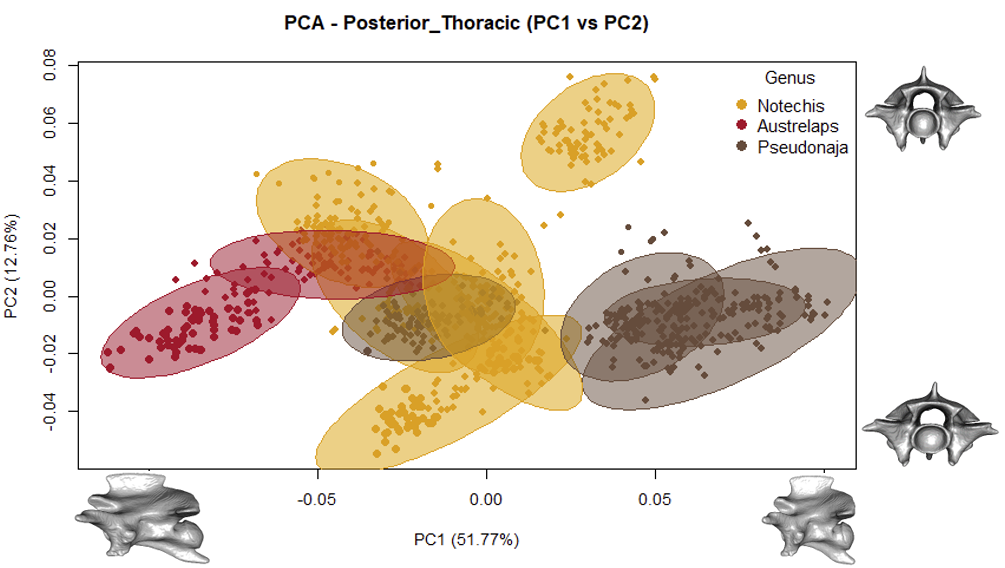


**Supplementary Figure 14:** PCA plot with PC1 and PC2 of all vertebrae in the posterior thoracic region. The ellipses show the 95% confidence ellipse for each snake with red representing *Austrelaps superbus*, gold representing *Notechis scutatus* and brown presenting *Pseudonaja textilis*. PC1 represented 51.77% of the total variation in the dataset and PC3 represents 12.76% of the total variation.


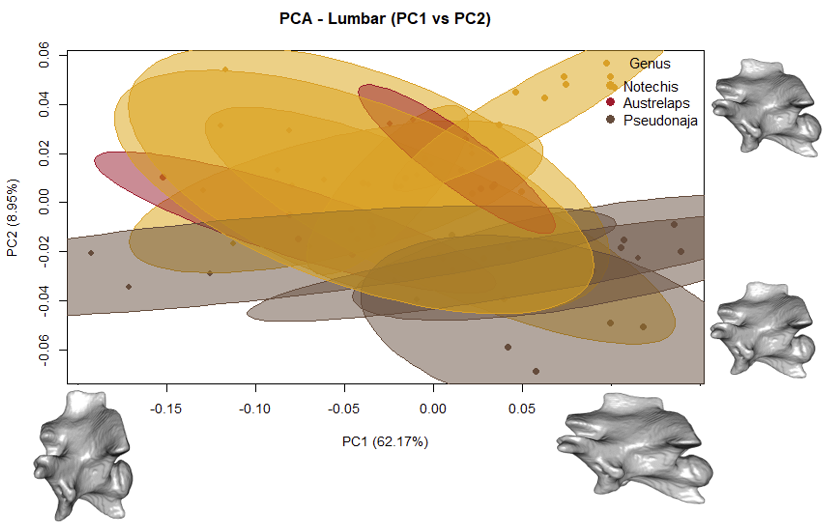


**Supplementary Figure 15:** PCA plot with PC1 and PC2 of all vertebrae in the lumbar region. The ellipses show the 95% confidence ellipse for each snake with red representing *Austrelaps superbus*, gold representing *Notechis scutatus* and brown presenting *Pseudonaja textilis*. PC1 represented 62.17% of the total variation in the dataset and PC2 represents 8.95% of the total variation.


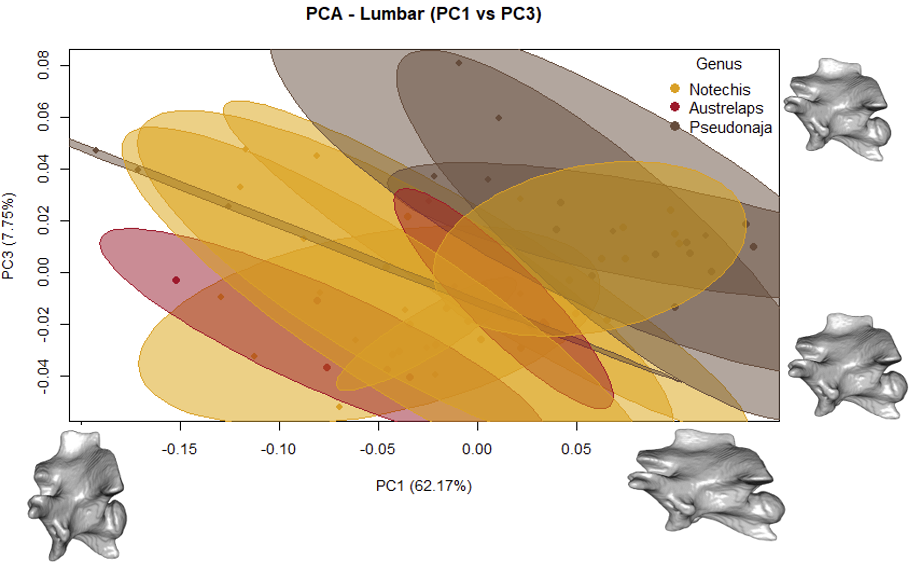


**Supplementary Figure 16:** PCA plot with PC1 and PC3 of all vertebrae in the lumbar region. The ellipses show the 95% confidence ellipse for each snake with red representing *Austrelaps superbus*, gold representing *Notechis scutatus* and brown presenting *Pseudonaja textilis*. PC1 represented 62.17% of the total variation in the dataset and PC3 represents 7.75% of the total variation.


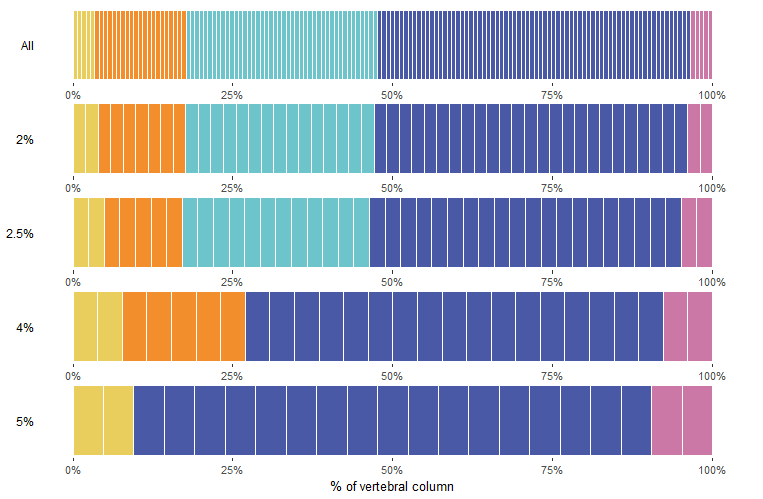


**Supplementary Figure 17:** Five vertebral maps from *Austrelaps_superbus*_MZRC_10088. From top to bottom: all vertebrae sampled, vertebra sampled from every 2%, 2.5%, 4% and 5% of the vertebral column. The best model according to Akaike Information Criterion (AICc).


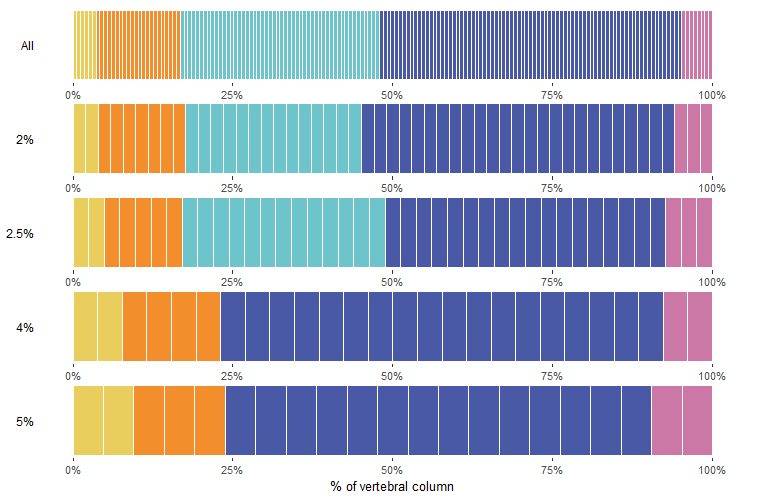


**Supplementary Figure 18**: shows five vertebral maps from *Notechis_scutatus*_MZRC_10089. In order from top to bottom there is: all vertebrae sampled, vertebra sampled from every 2%, 2.5%, 4% and 5% of the vertebral column. The best model according to Akaike Interaction Criterion (AIC).


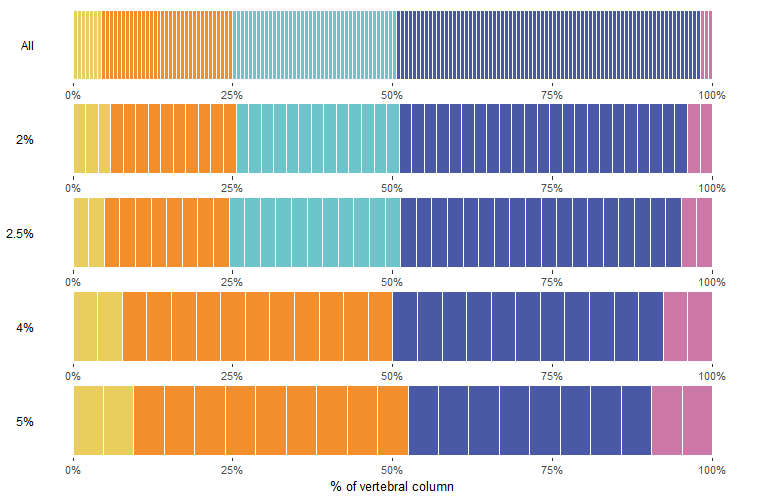


**Supplementary Figure 19**: shows five vertebral maps from *Notechis_scutatus*_NMV_D76365. In order from top to bottom there is: all vertebrae sampled, vertebra sampled from every 2%, 2.5%, 4% and 5% of the vertebral column. The best model according to Akaike Interaction Criterion (AIC).


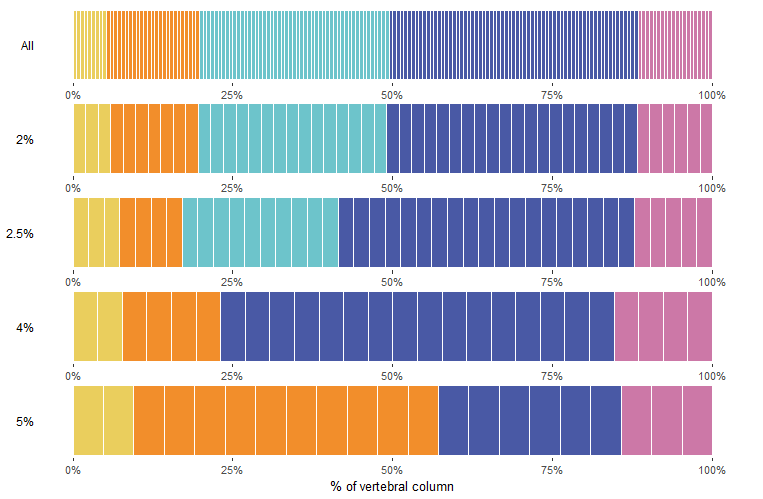


**Supplementary Figure 20:** shows five vertebral maps from *Notechis_scutatus*_NMV_D76366. In order from top to bottom there is: all vertebrae sampled, vertebra sampled from every 2%, 2.5%, 4% and 5% of the vertebral column. The best model according to Akaike Interaction Criterion (AIC).


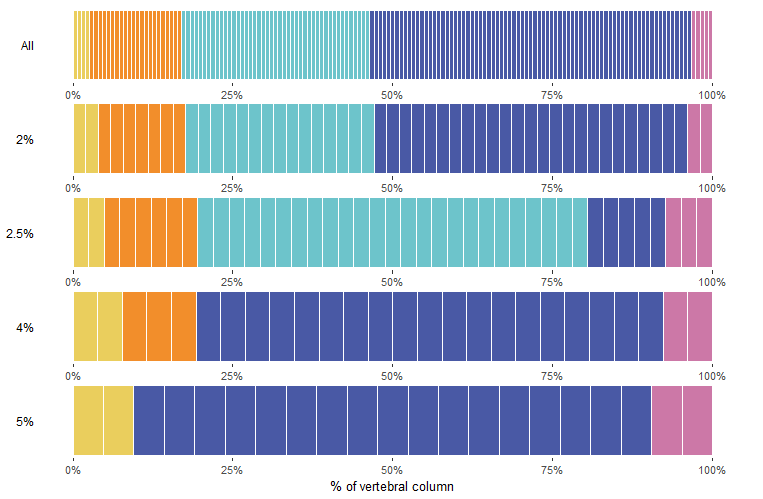
**Supplementary Figure 21:** shows five vertebral maps from *Notechis_scutatus*_NMV_Z77599. In order from top to bottom there is: all vertebrae sampled, vertebra sampled from every 2%, 2.5%, 4% and 5% of the vertebral column. The best model according to Akaike Interaction Criterion (AIC).
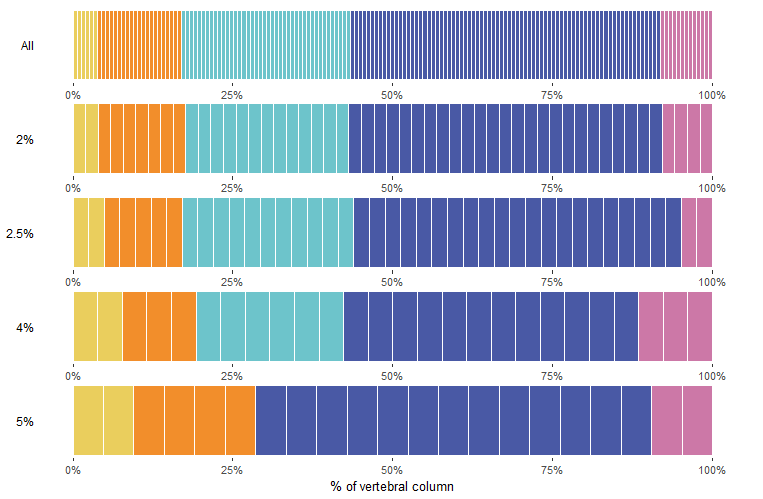


**Supplementary Figure 22:** shows five vertebral maps from *Notechis_scutatus*_NMV_Z77600. In order from top to bottom there is: all vertebrae sampled, vertebra sampled from every 2%, 2.5%, 4% and 5% of the vertebral column. The best model according to Akaike Interaction Criterion (AIC).


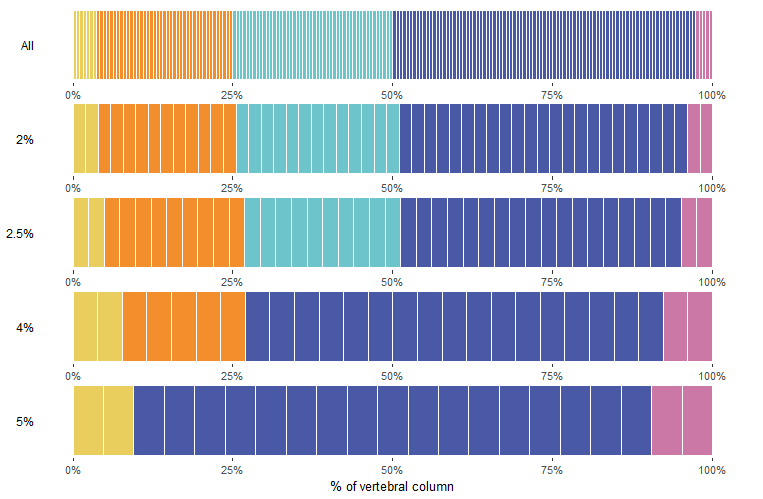
 **Supplementary Figure 23:** shows five vertebral maps from *Pseudonaja_textilis*_MZRC_10093. In order from top to bottom there is: all vertebrae sampled, vertebra sampled from every 2%, 2.5%, 4% and 5% of the vertebral column. The best model according to Akaike Interaction Criterion (AIC).


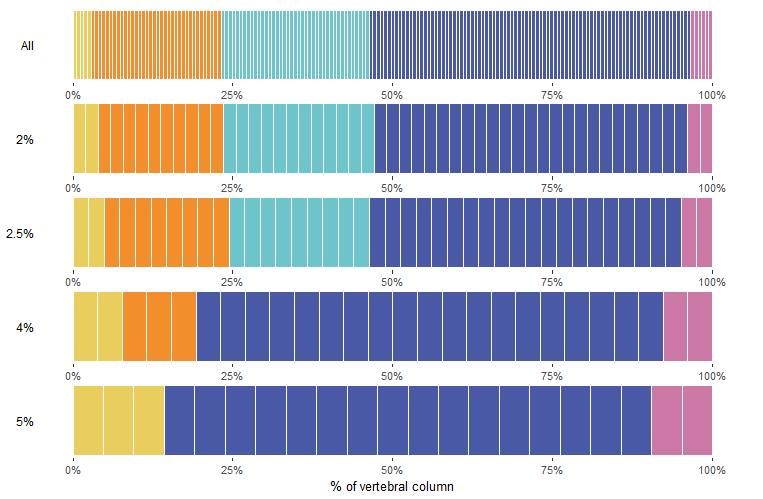


**Supplementary Figure 24:** shows five vertebral maps from *Pseudonaja_textilis*_NMV_D76368. In order from top to bottom there is: all vertebrae sampled, vertebra sampled from every 2%, 2.5%, 4% and 5% of the vertebral column. The best model according to Akaike Interaction Criterion (AIC).

**
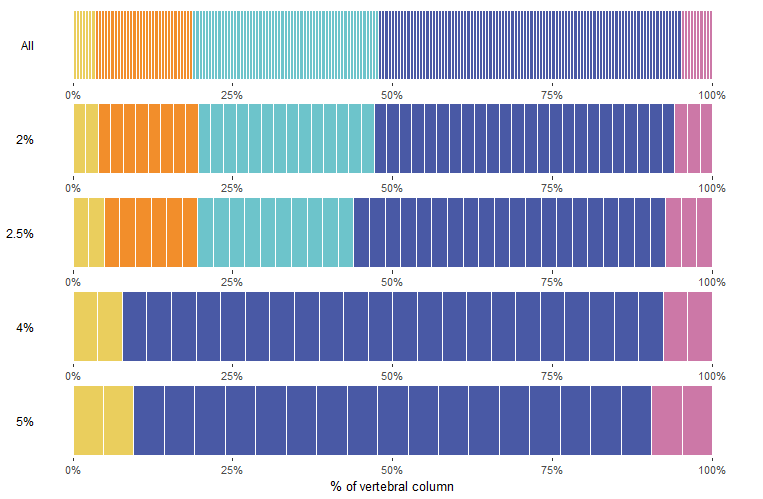
Supplementary Figure 25:** shows five vertebral maps from *Pseudonaja_textilis*_NMV_D76369. In order from top to bottom there is: all vertebrae sampled, vertebra sampled from every 2%, 2.5%, 4% and 5% of the vertebral column. The best model according to Akaike Interaction Criterion (AIC).


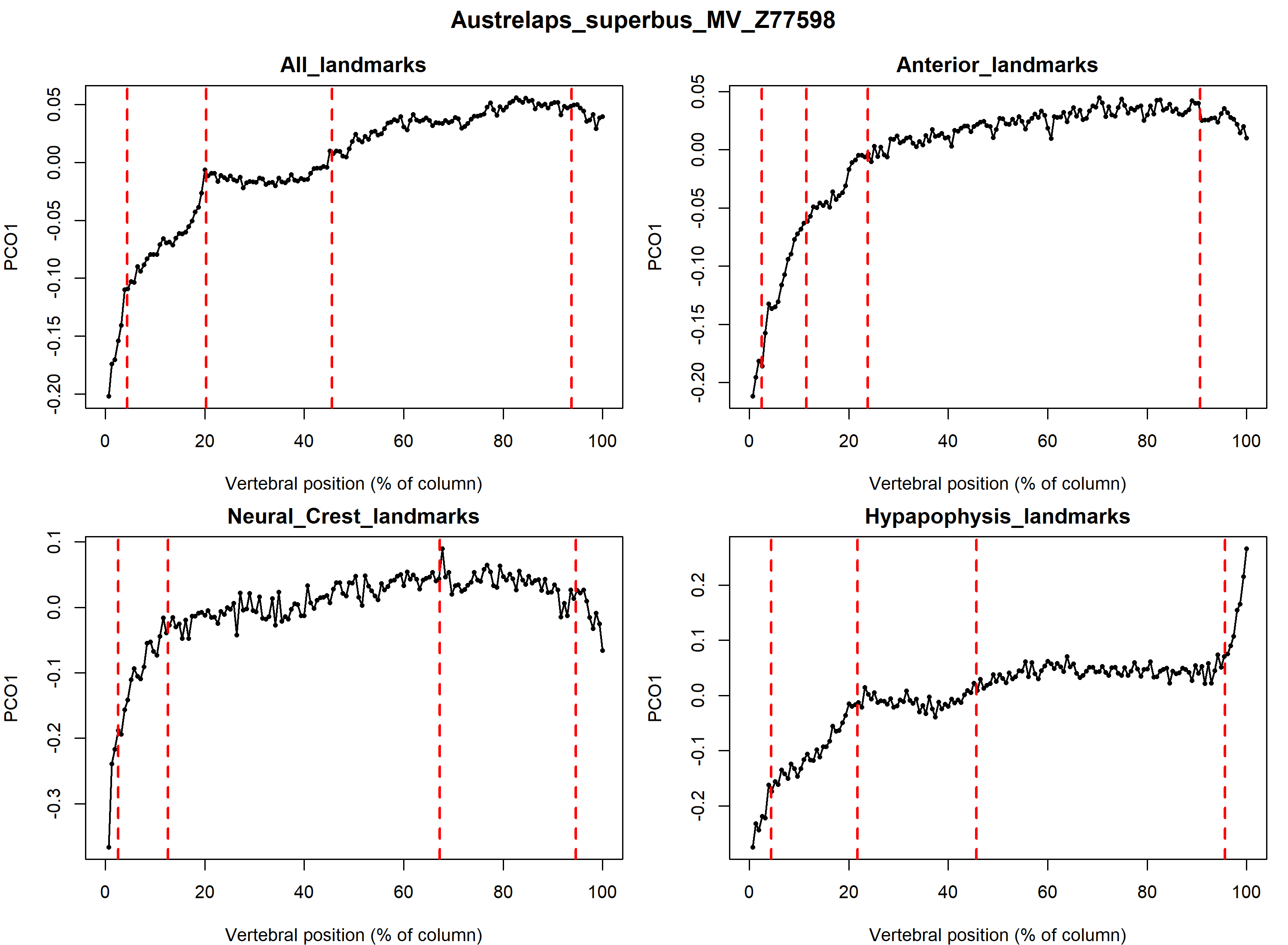

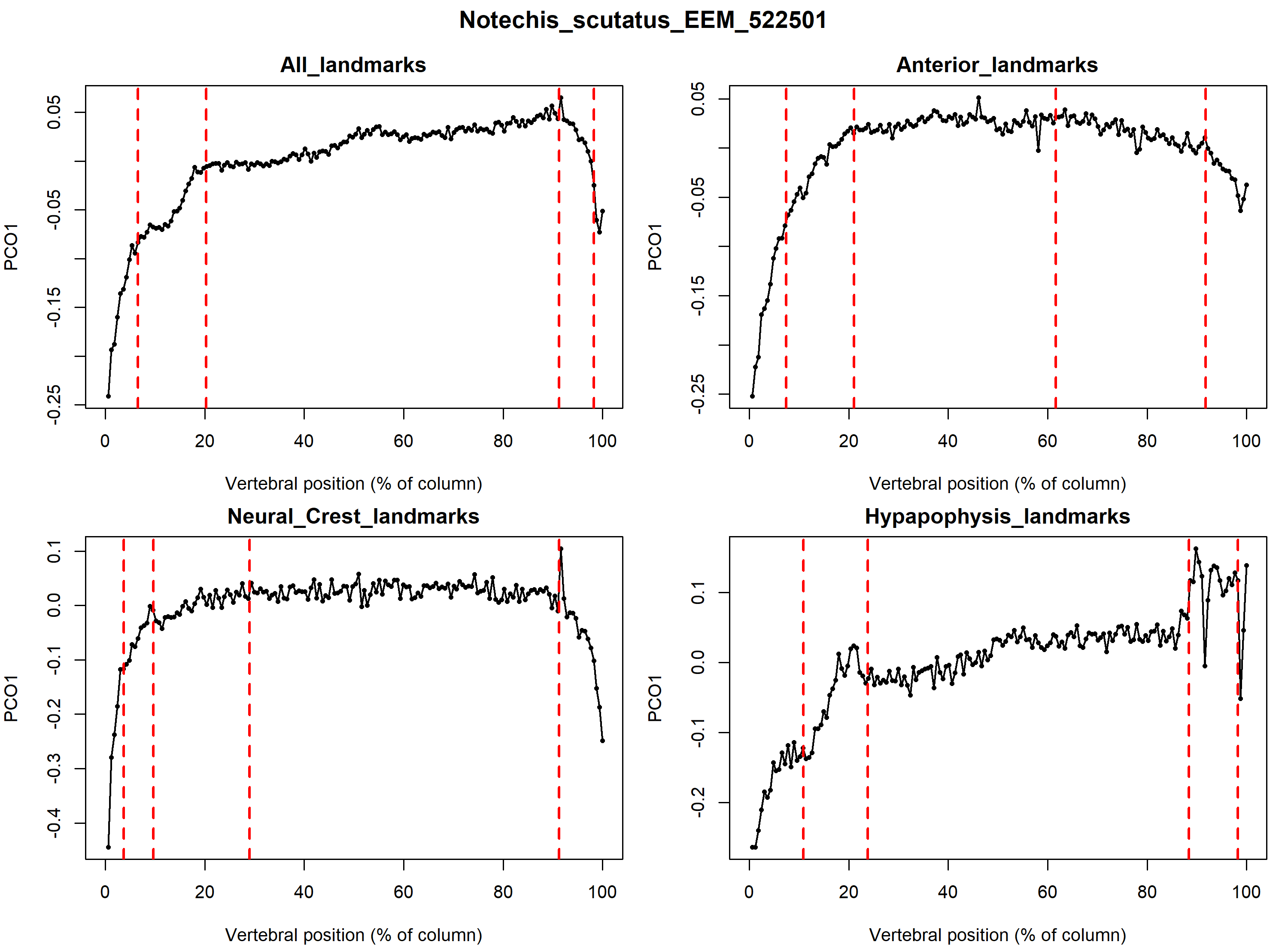
**Supplementary Figure 26:** Four segmented linear regressions from four landmarking schemes of the individual snake *Austrelaps superbus* NMV Z77598. The number and position of breakpoints were determined via Akaike Information Criterion (AIC) and represented by the dashed red lines.

**Supplementary Figure 27:** Four segmented linear regressions from four landmarking schemes of the individual snake *Notechis scutatus* EEM 522501. The number and position of breakpoints were determined via Akaike Information Criterion (AIC) and represented by the dashed red lines.


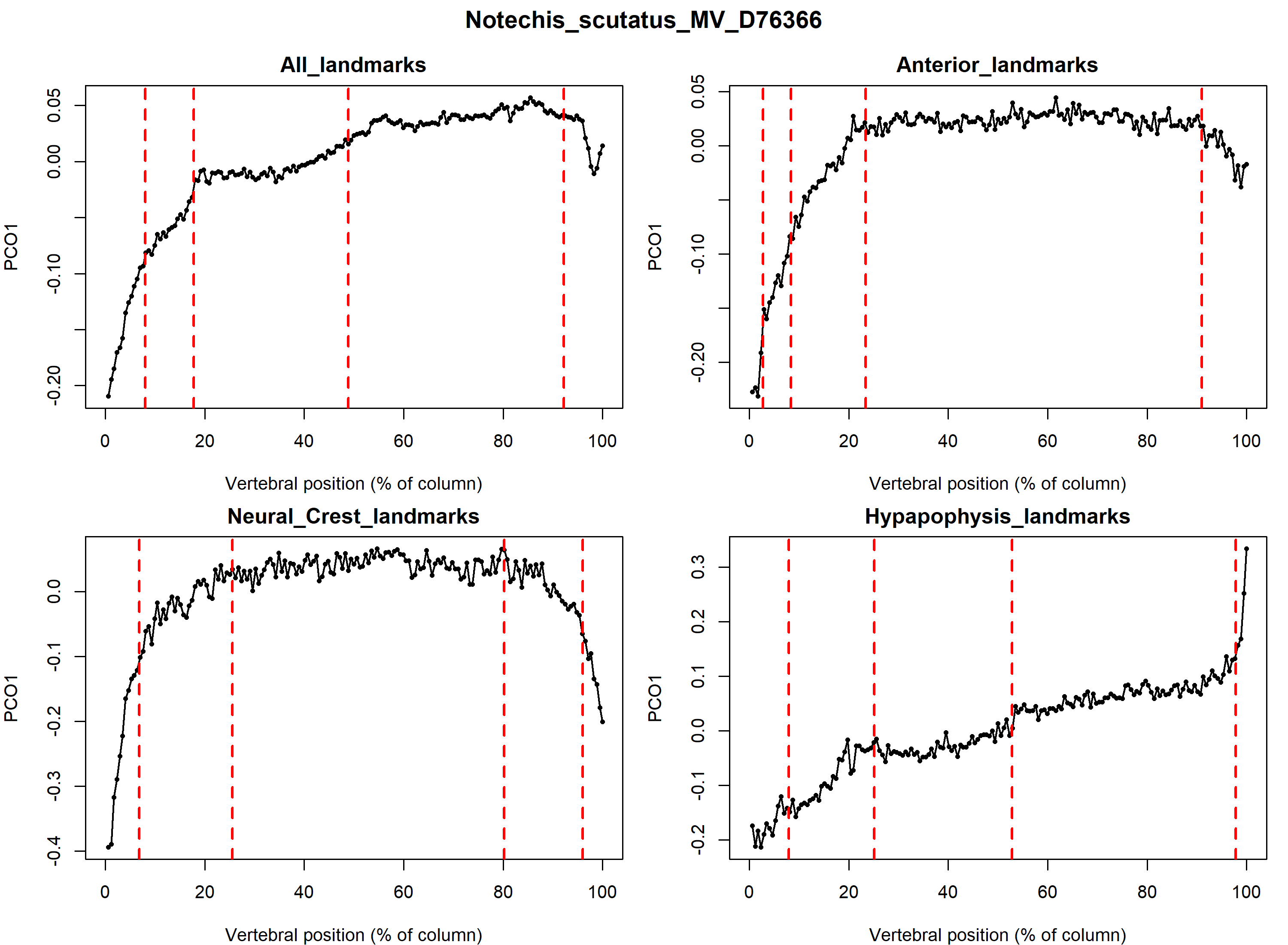

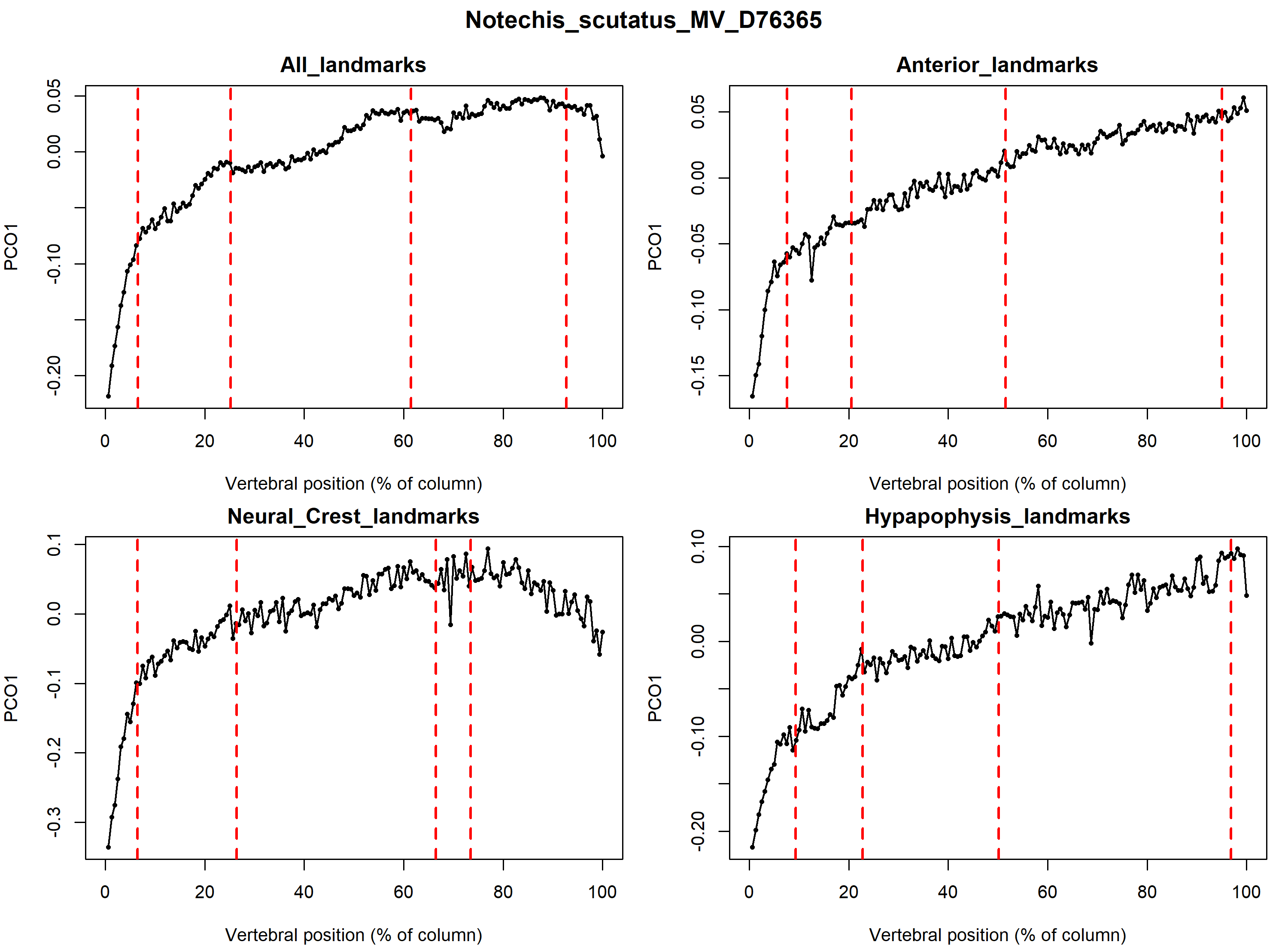
**Supplementary Figure 28:** Four segmented linear regressions from four landmarking schemes of the individual snake *Notechis scutatus* NMV D76365. The number and position of breakpoints were determined via Akaike Information Criterion (AIC) and represented by the dashed red lines.

**Supplementary Figure 29:** Four segmented linear regressions from four landmarking schemes of the individual snake *Notechis scutatus* NMV D76366. The number and position of breakpoints were determined via Akaike Information Criterion (AIC) and represented by the dashed red lines.


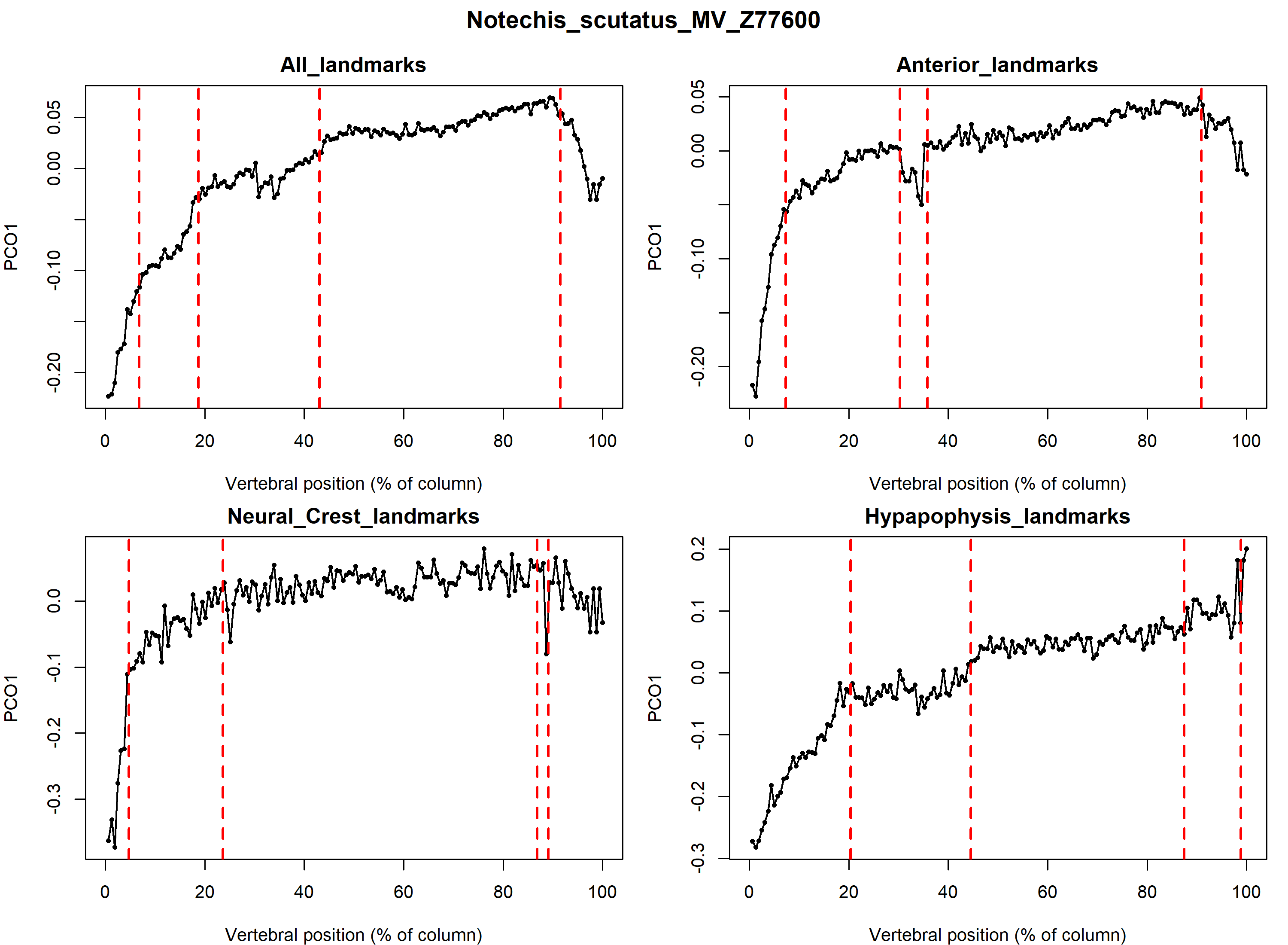

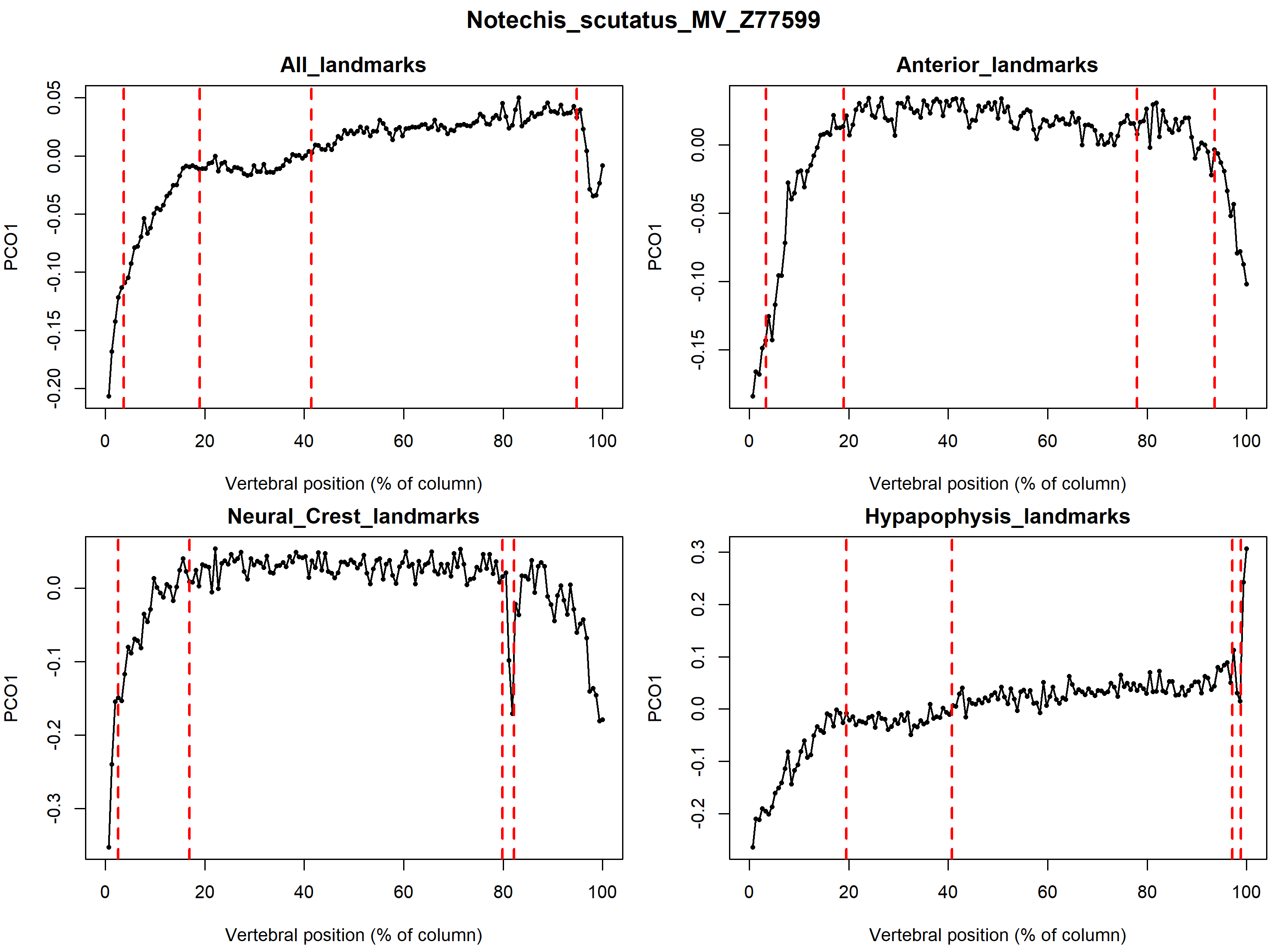
**Supplementary Figure 30:** Four segmented linear regressions from four landmarking schemes of the individual snake *Notechis scutatus* NMV Z77599. The number and position of breakpoints were determined via Akaike Information Criterion (AIC) and represented by the dashed red lines.

**Supplementary Figure 31:** Four segmented linear regressions from four landmarking schemes of the individual snake *Notechis scutatus* NMV Z77600. The number and position of breakpoints were determined via Akaike Information Criterion (AIC) and represented by the dashed red lines.


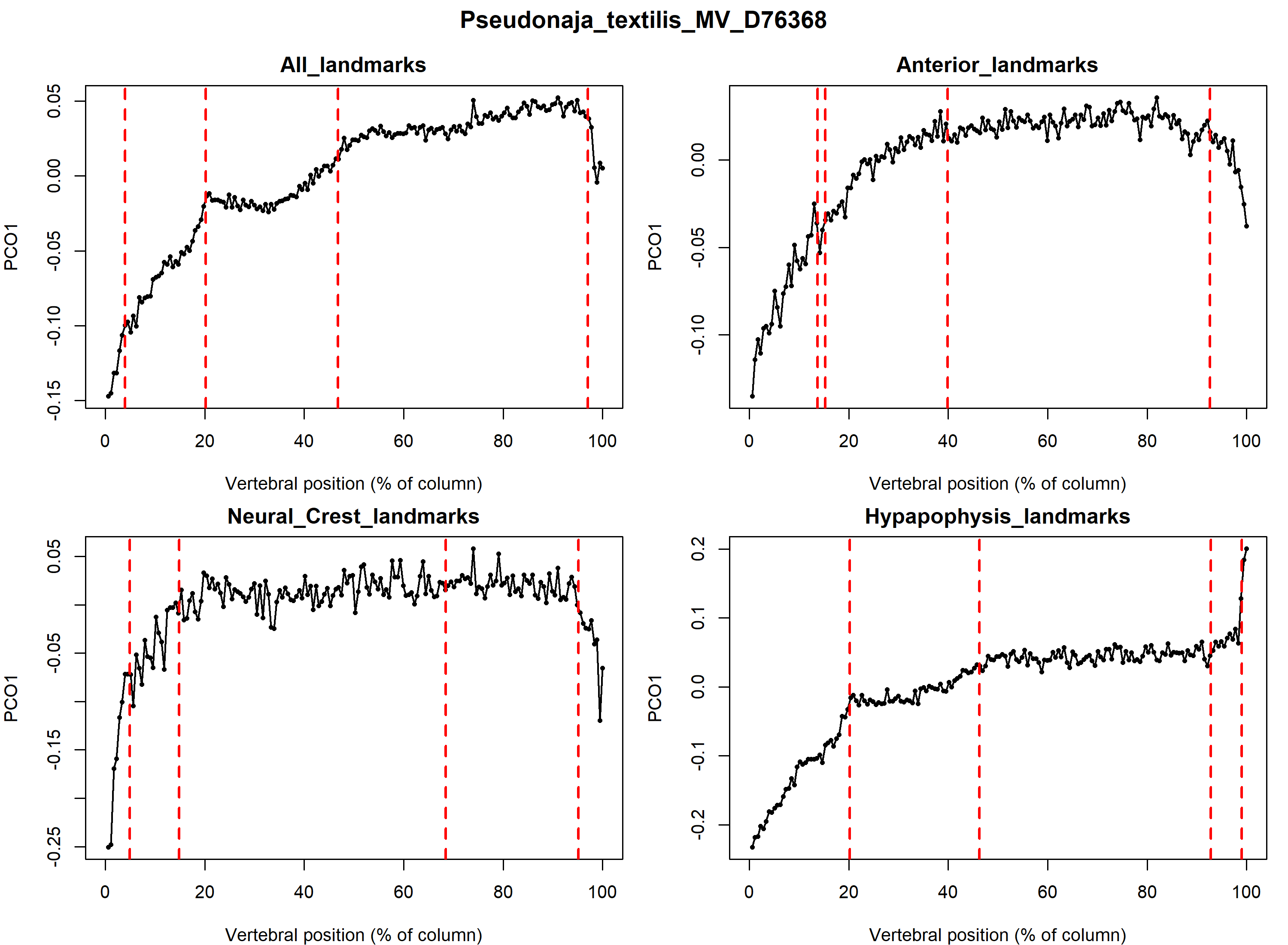

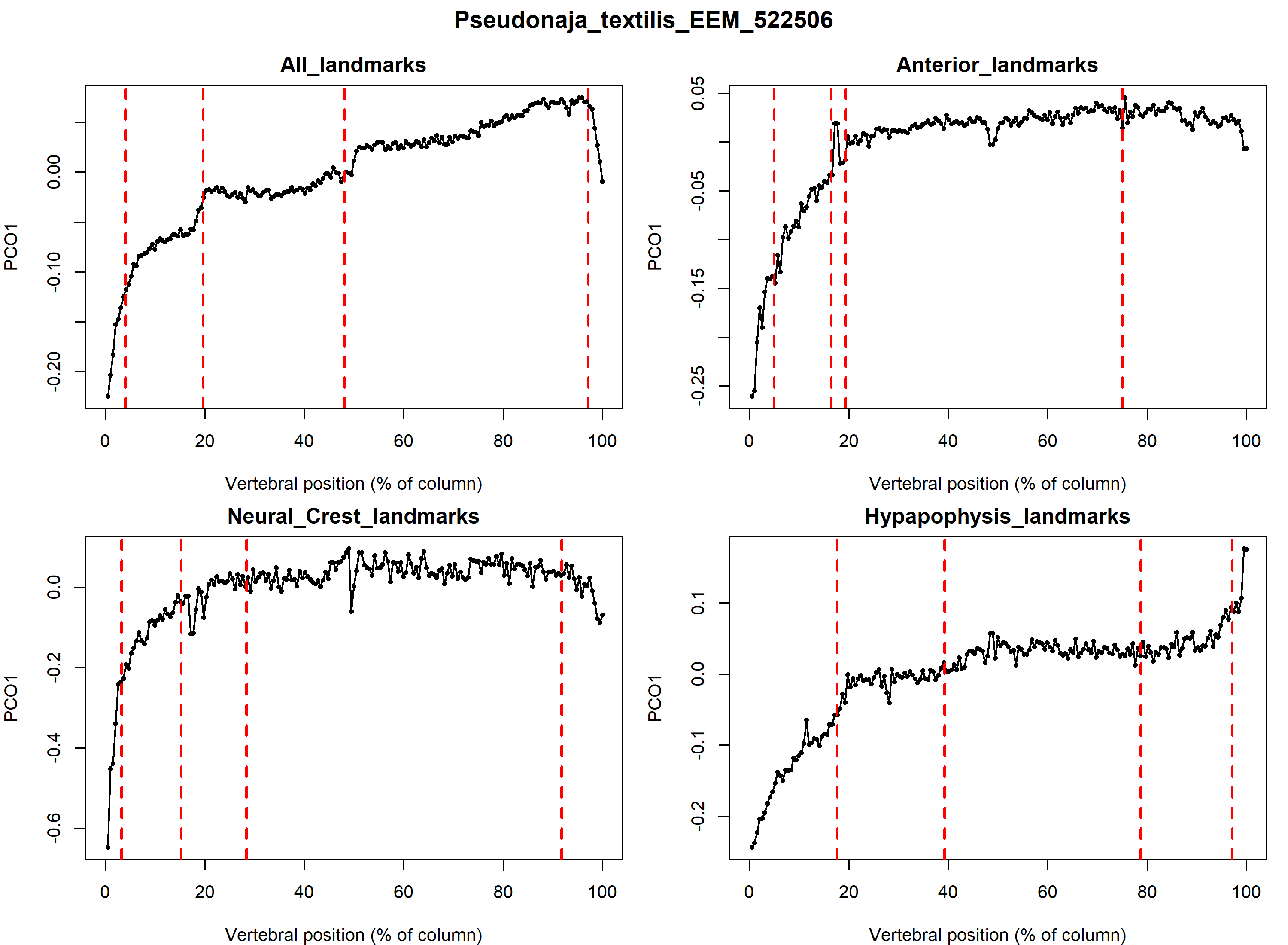
**Supplementary Figure 32:** Four segmented linear regressions from four landmarking schemes of the individual snake *Pseudonaja textilis* EEM 522506. The number and position of breakpoints were determined via Akaike Information Criterion (AIC) and represented by the dashed red lines.

**Supplementary Figure 33:** Four segmented linear regressions from four landmarking schemes of the individual snake *Pseudonaja textilis* NMV D76368. The number and position of breakpoints were determined via Akaike Information Criterion (AIC) and represented by the dashed red lines.


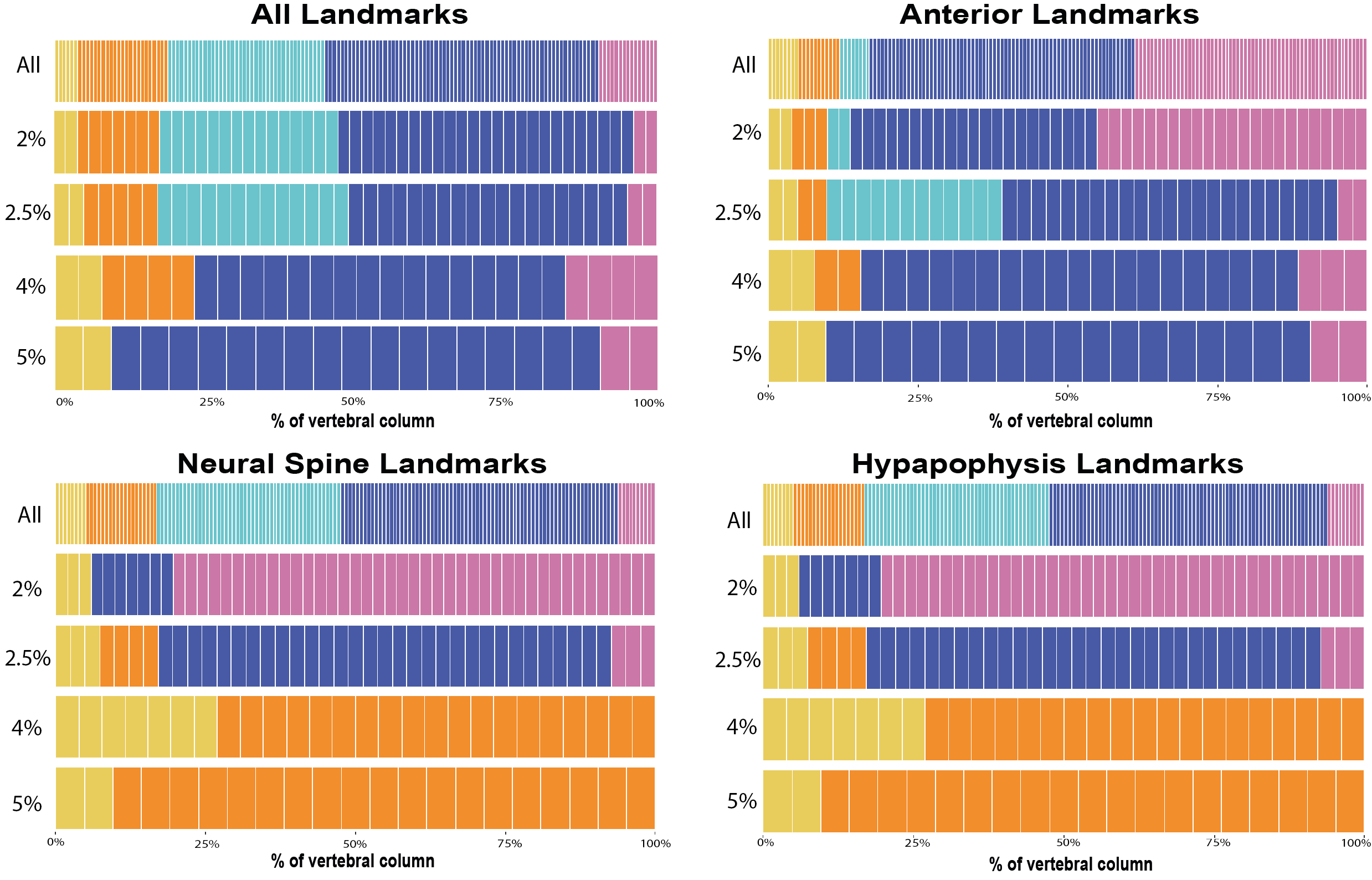

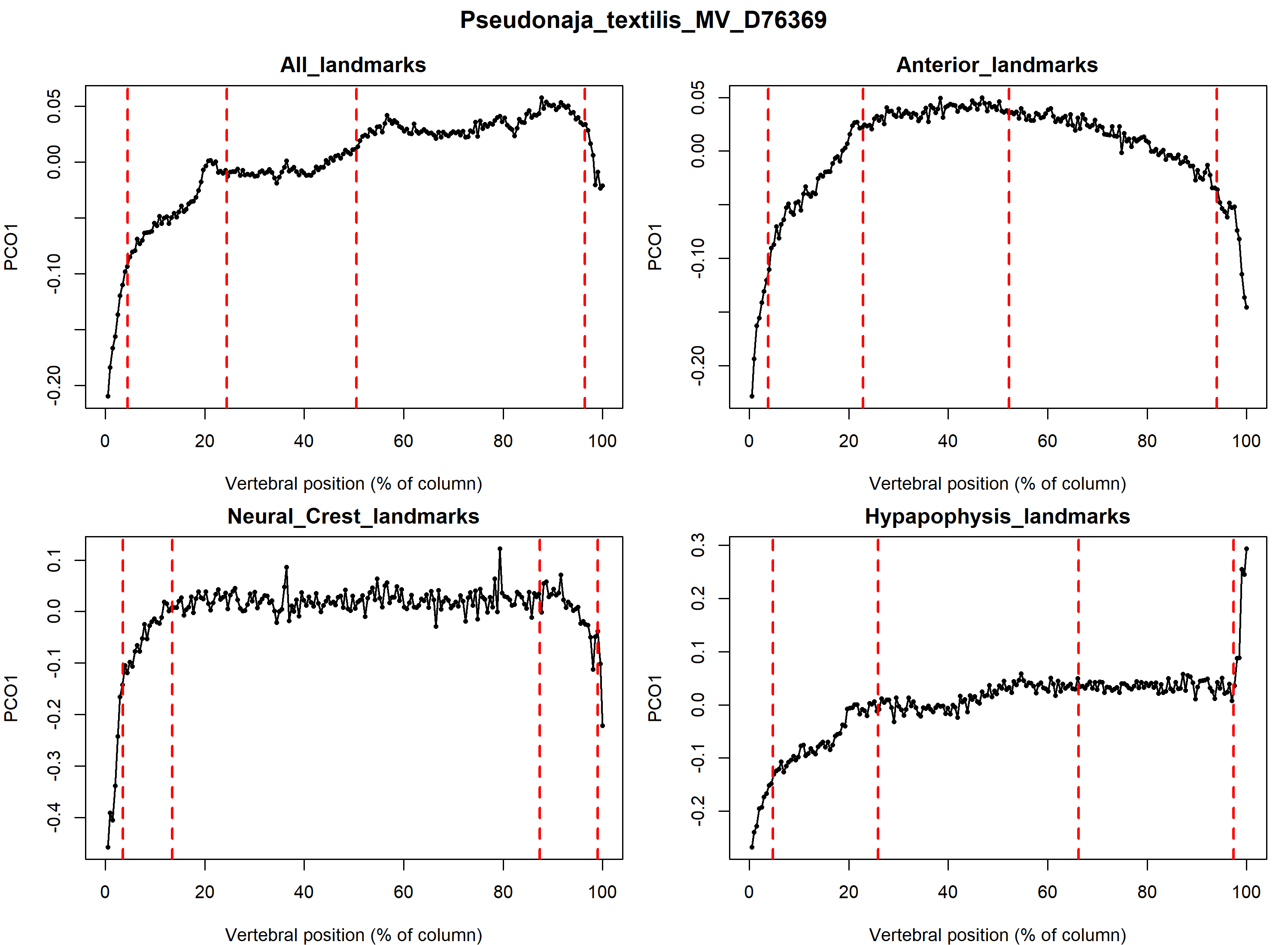
**Supplementary Figure 34:** Four segmented linear regressions from four landmarking schemes of the individual snake *Pseudonaja textilis* NMV D76369. The number and position of breakpoints were determined via Akaike Information Criterion (AIC) and represented by the dashed red lines.

**Supplementary Figure 35:** Subsampling of the 4 landmarking schemes for a snake, *Austrelaps superbus* NMV Z77598. Each square represents a vertebra and each colour shows a distinct region and their size. The subsampling intervals are every 2%, 2.5%, 4% and 5% of the precaudal column.


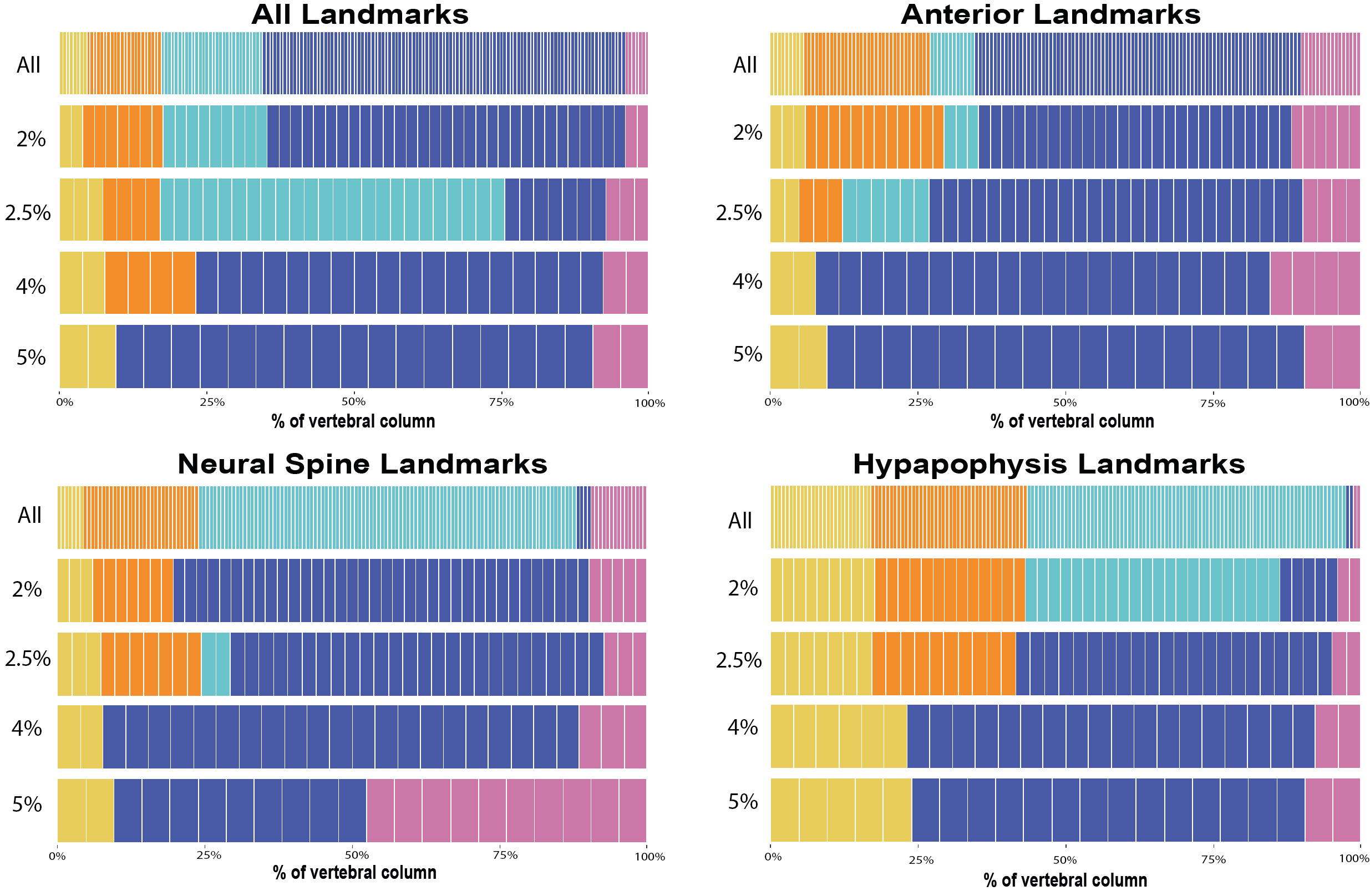

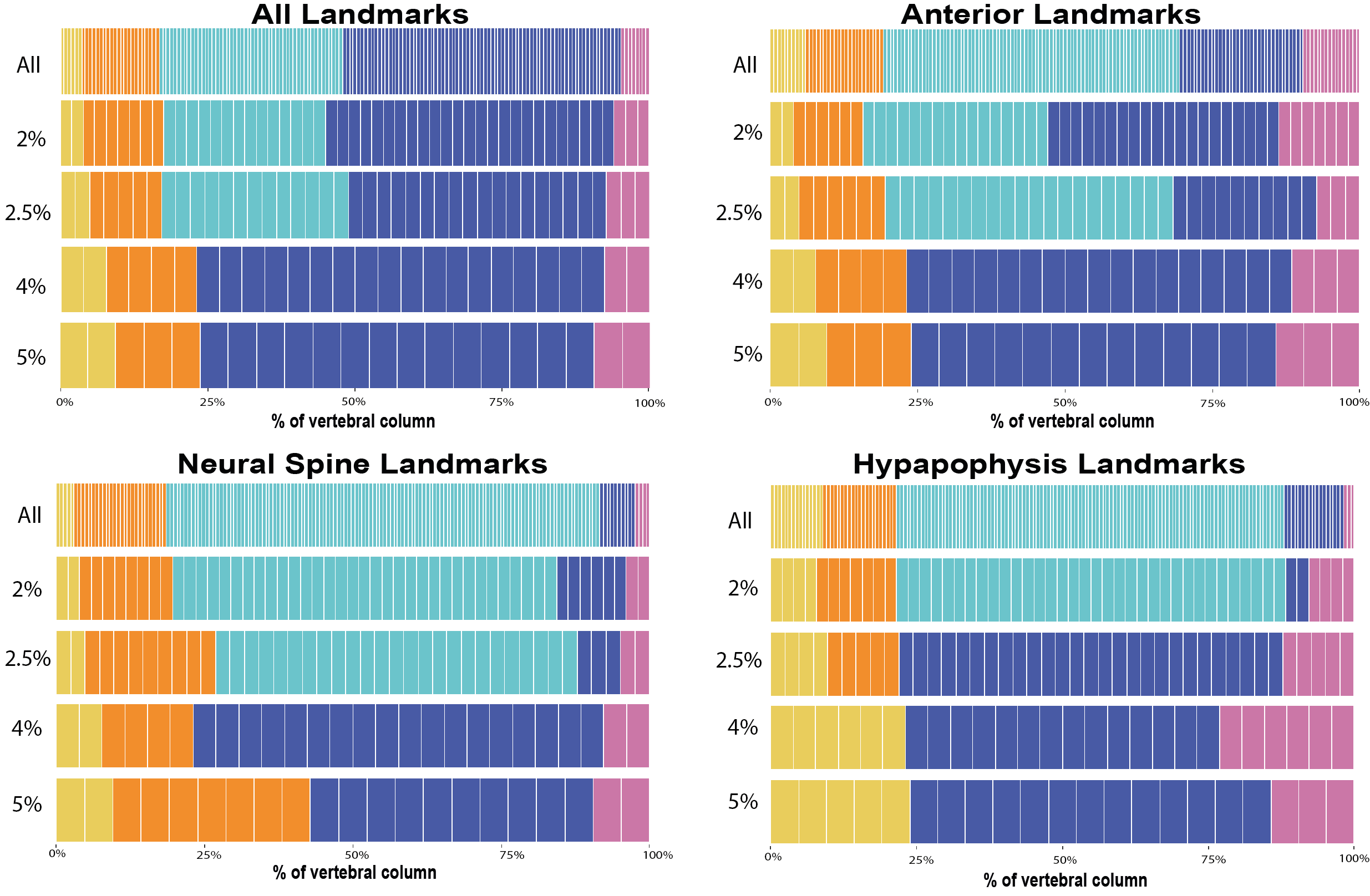
**Supplementary Figure 36:** Subsampling of the 4 landmarking schemes for a snake, *Notechis scutatus* EEM 522501. Each square represents a vertebra and each colour shows a distinct region and their size. The subsampling intervals are every 2%, 2.5%, 4% and 5% of the precaudal column.

**Supplementary Figure 37:** Subsampling of the 4 landmarking schemes for a snake, *Notechis scutatus* EEM 522504. Each square represents a vertebra and each colour shows a distinct region and their size. The subsampling intervals are every 2%, 2.5%, 4% and 5% of the precaudal column.


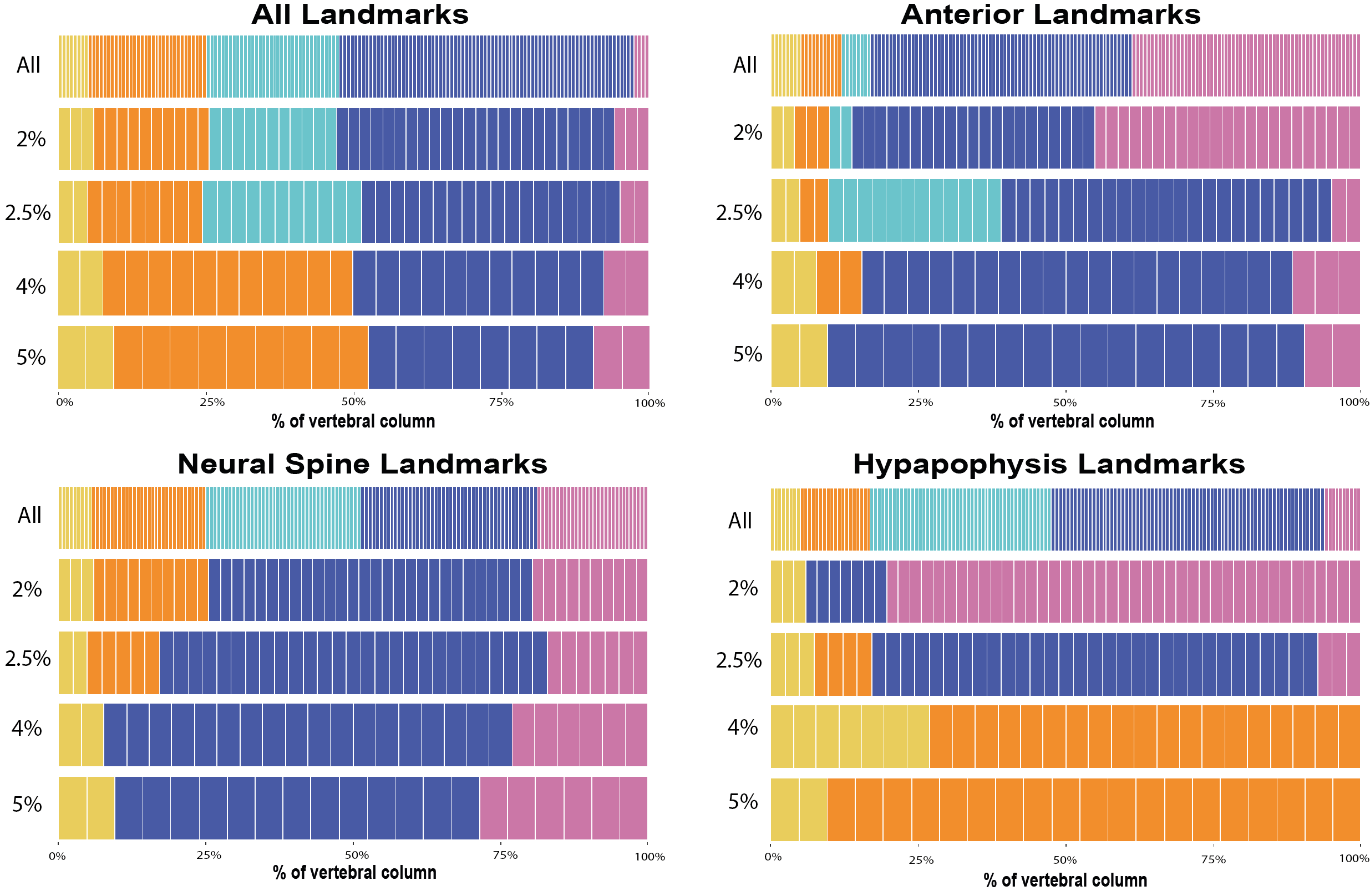


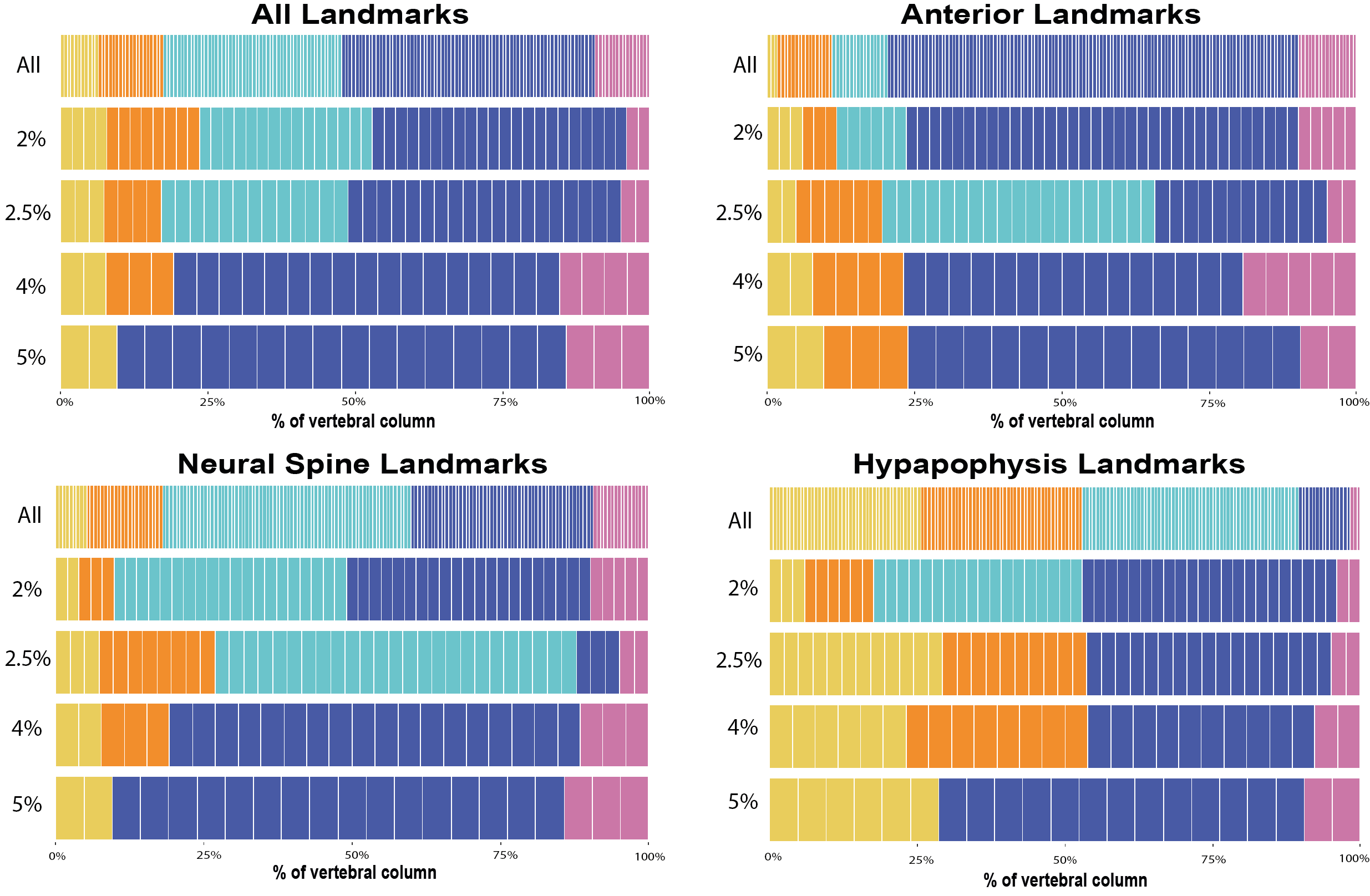
**Supplementary Figure 38:** Subsampling of the 4 landmarking schemes for a snake, *Notechis scutatus* NMV D76365. Each square represents a vertebra and each colour shows a distinct region and their size. The subsampling intervals are every 2%, 2.5%, 4% and 5% of the precaudal column.

**Supplementary Figure 39:** Subsampling of the 4 landmarking schemes for a snake, *Notechis scutatus* NMV D76366. Each square represents a vertebra and each colour shows a distinct region and their size. The subsampling intervals are every 2%, 2.5%, 4% and 5% of the precaudal column.


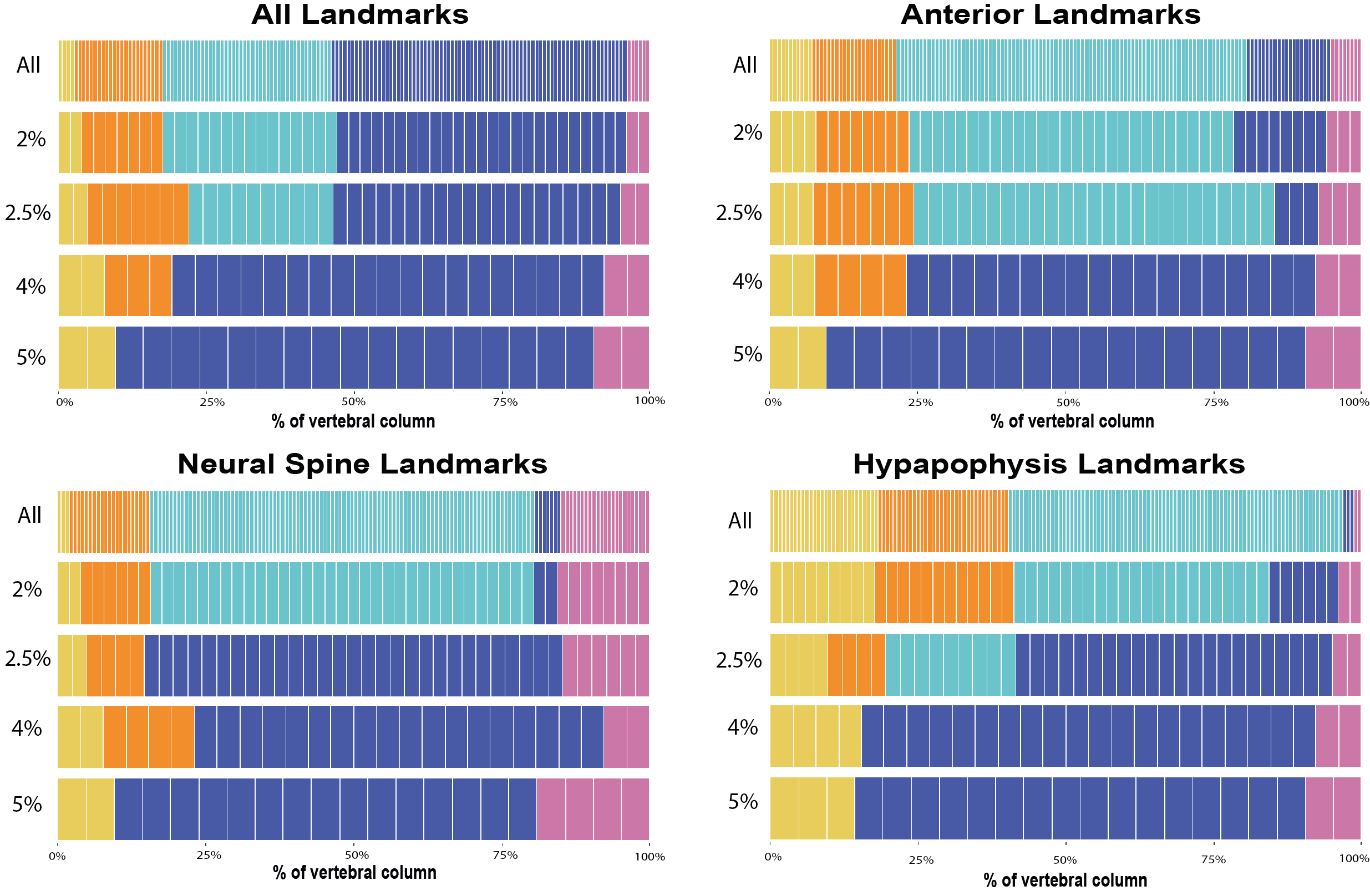
**Supplementary Figure 40:** Subsampling of the 4 landmarking schemes for a snake, *Notechis scutatus* NMV Z77599. Each square represents a vertebra and each colour shows a distinct region and their size. The subsampling intervals are every 2%, 2.5%, 4% and 5% of the precaudal column.


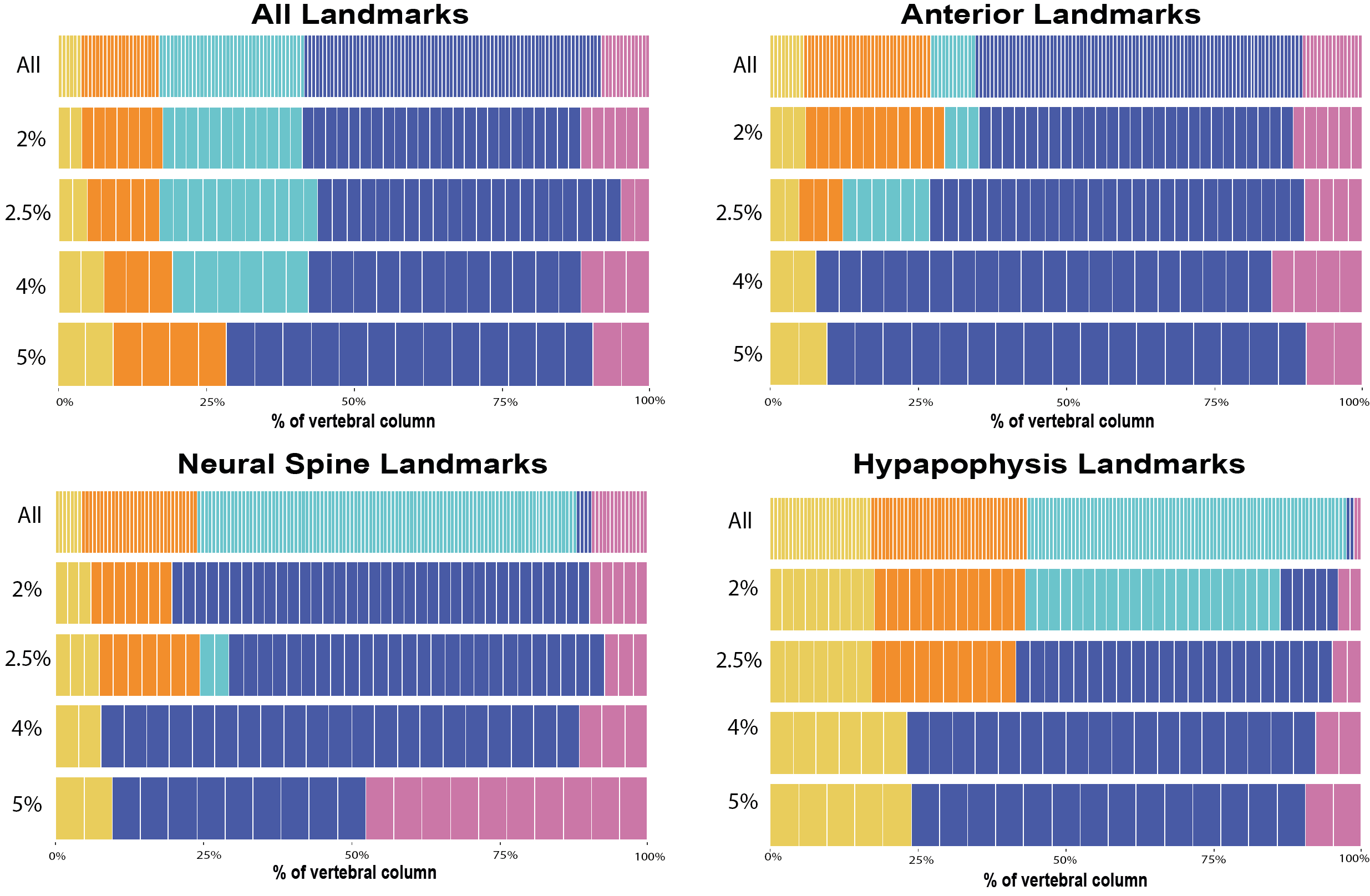


**Supplementary Figure 41:** Subsampling of the 4 landmarking schemes for a snake, *Notechis scutatus* NMV Z77600. Each square represents a vertebra and each colour shows a distinct region and their size. The subsampling intervals are every 2%, 2.5%, 4% and 5% of the precaudal column.


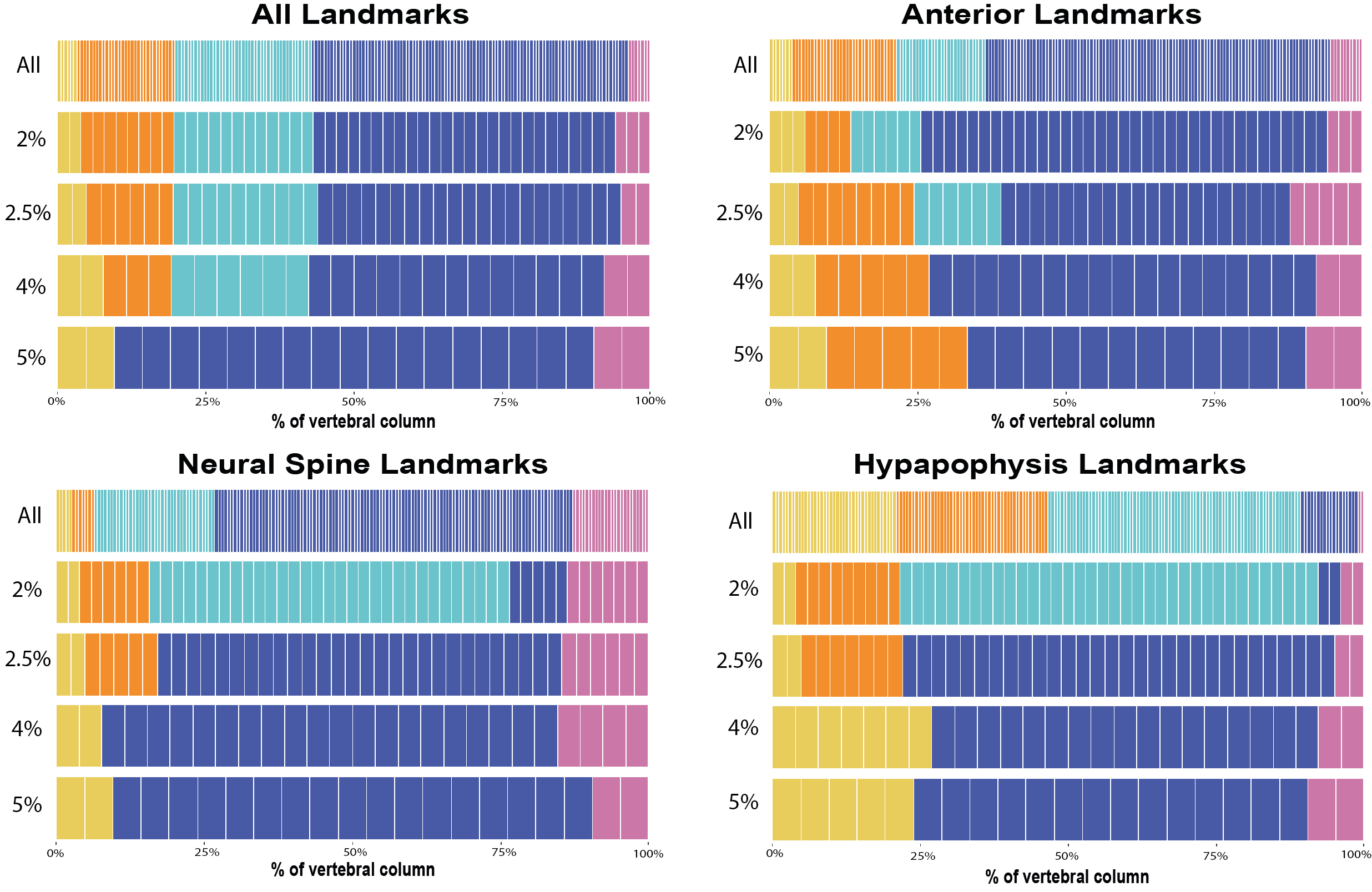

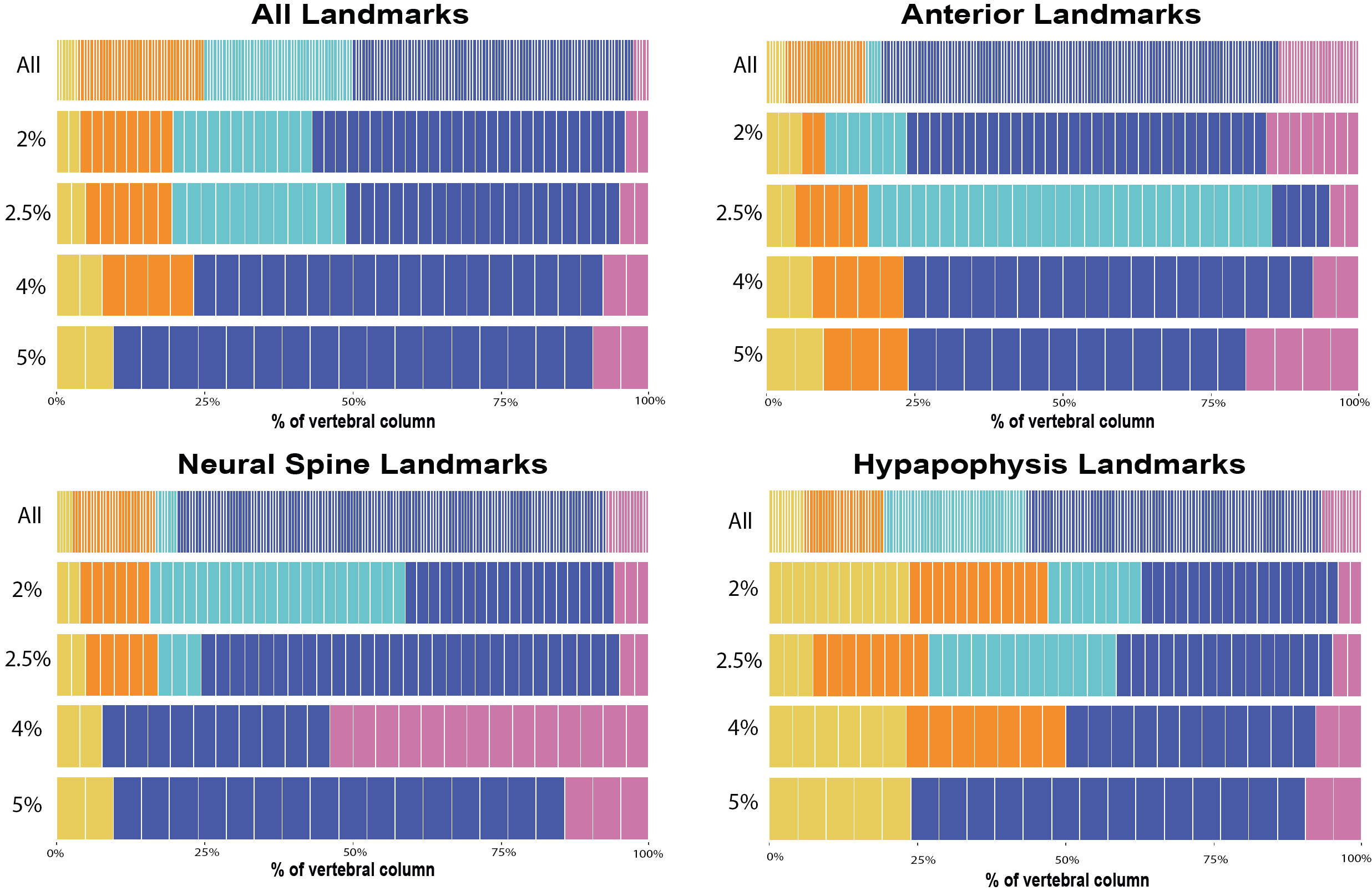
**Supplementary Figure 42:** Subsampling of the 4 landmarking schemes for a snake, *Pseudonaja textilis* EEM 522506. Each square represents a vertebra and each colour shows a distinct region and their size. The subsampling intervals are every 2%, 2.5%, 4% and 5% of the precaudal column.

**Supplementary Figure 43:** Subsampling of the 4 landmarking schemes for a snake, *Pseudonaja textilis* NMV D76367. Each square represents a vertebra and each colour shows a distinct region and their size. The subsampling intervals are every 2%, 2.5%, 4% and 5% of the precaudal column.


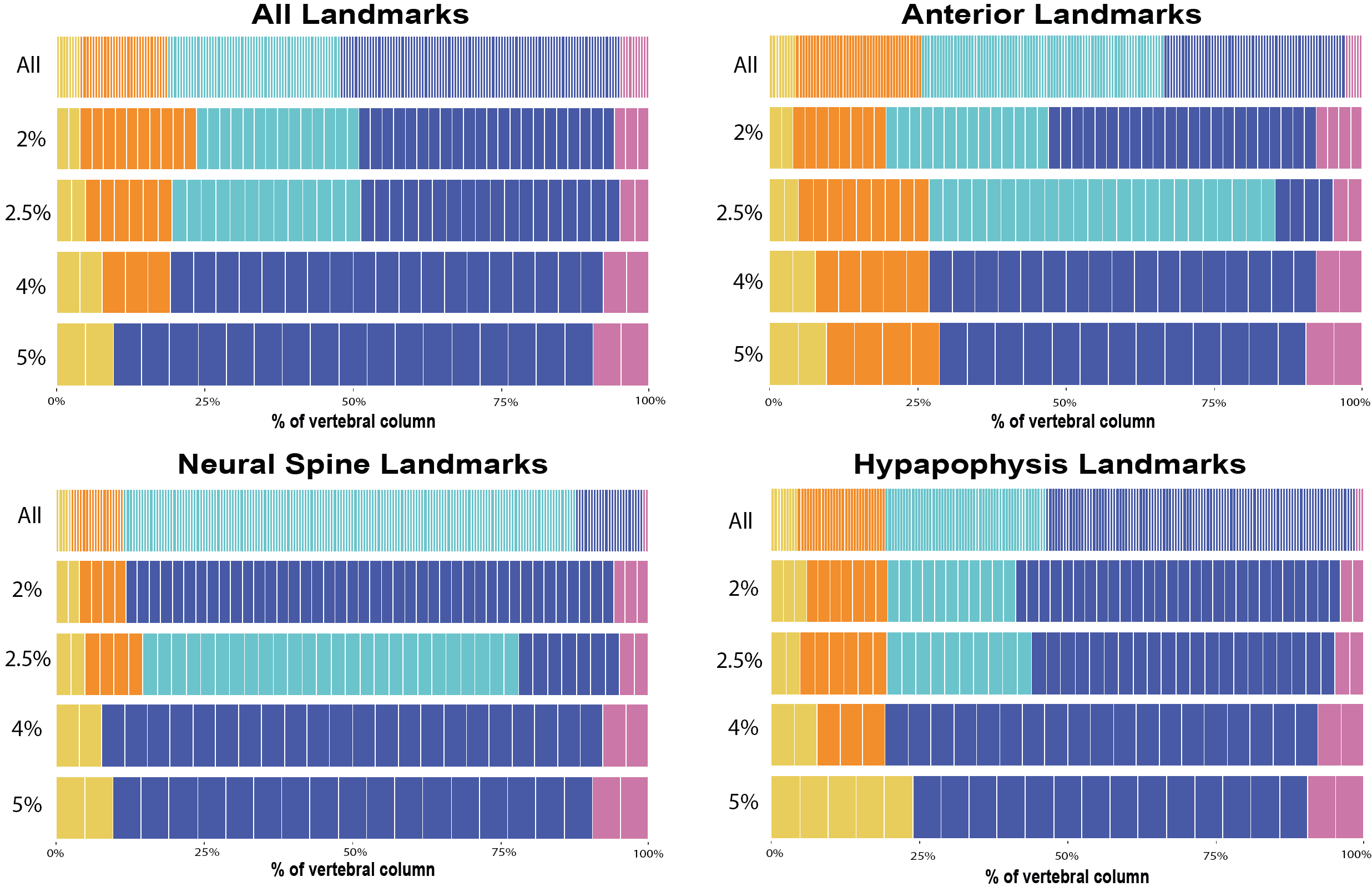

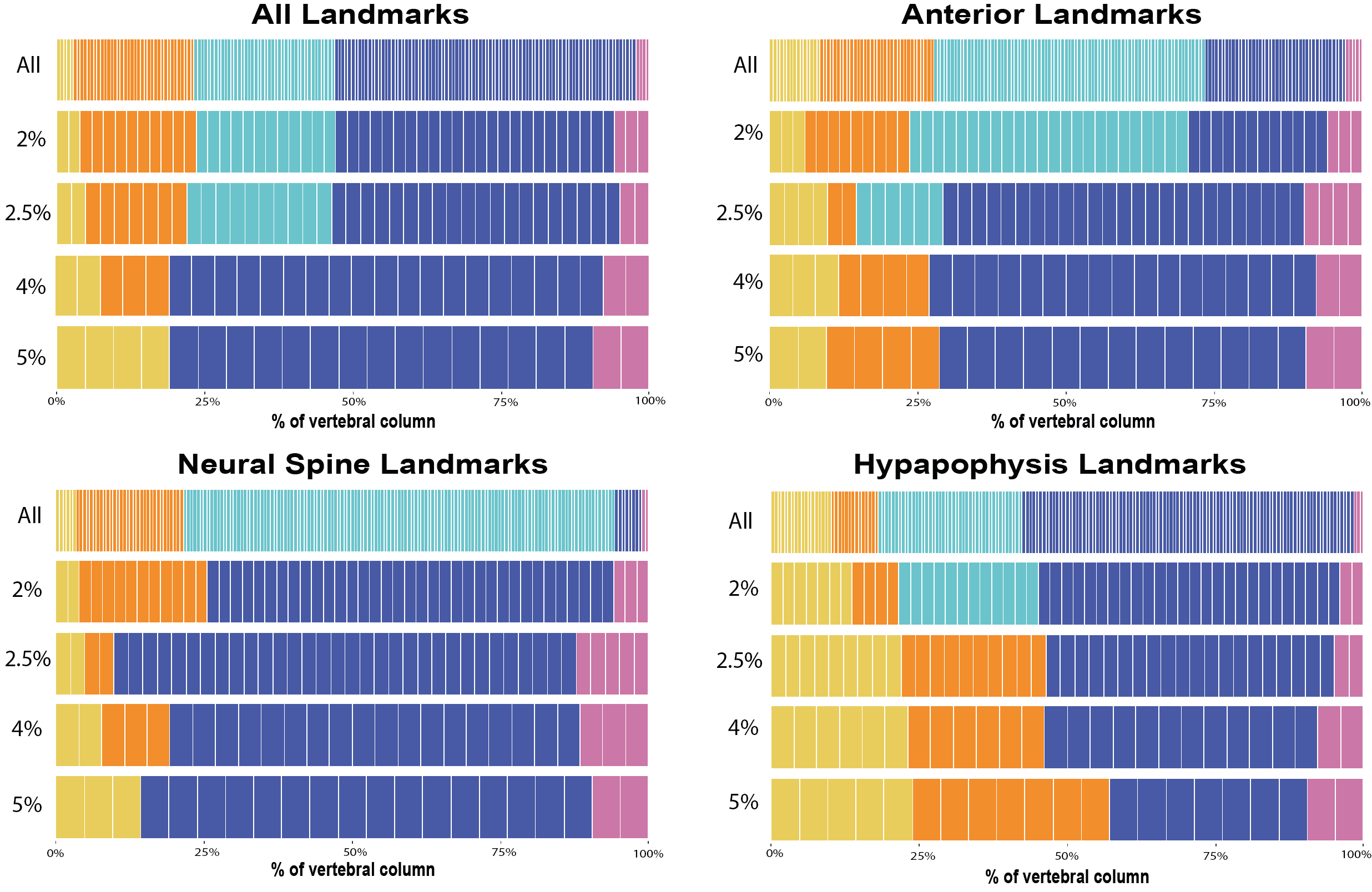
**Supplementary Figure 44:** Subsampling of the 4 landmarking schemes for a snake, *Pseudonaja textilis* NMV D76368. Each square represents a vertebra and each colour shows a distinct region and their size. The subsampling intervals are every 2%, 2.5%, 4% and 5% of the precaudal column.

**Supplementary Figure 45:** Subsampling of the 4 landmarking schemes for a snake, *Pseudonaja textilis* NMV D76369. Each square represents a vertebra and each colour shows a distinct region and their size. The subsampling intervals are every 2%, 2.5%, 4% and 5% of the precaudal column.

**Supplementary Table 1**: Individual snakes of this study, with their sex, lengths (total and snout-to-vent length, SVL), mass, vertebral counts of the pre-caudal and caudal regions, and heart positions against the number of vertebrae from the head, and as a percentage of the pre-caudal column. All snakes measured were adults.

| **Specimen ID** | **Genus** | **Species** | **Common Name** | **Sex** | **Length (cm)** | | **Mass (g)** | **Vertebrae count** | | | | **Heart Position** | | | **Heart Position (%)** | | |
| --- | --- | --- | --- | --- | --- | --- | --- | --- | --- | --- | --- | --- | --- | --- | --- | --- | --- |
|  |  |  |  |  | **Total** | **SVL** |  | **Pre-caudal** | **Caudal** | **Total** | **Start** | | **End** | **Start** | | **End** |  |
| Austrelaps_superbus_MZRC_10088 | *Austrelaps* | *superbus* | Lowland Copperhead | Male | 115.5 | 98.5 | 781.8 | 153 | 35 | 191 | 26 | | 31 | 16.7 | | 19.9 |  |
| Austrelaps_superbus_MV_Z77598 | *Austrelaps* | *superbus* | Lowland Copperhead | Female | 84 | 69.5 | NA | 155 |  | 157 | 27 | | 32 | 15.9 | | 19.1 |  |
| Notechis_scutatus_MZRC_10089 | *Notechis* | *scutatus* | Tiger Snake | Male | N/A | 78 | 189 | 169 | 49 | 221 | 32 | | 38 | 18.6 | | 22.1 |  |
| Notechis_scutatus_MZRC_10091 | *Notechis* | *scutatus* | Tiger Snake | Female | 114.2 | 99 | 452.11 | 174 | 52 | 229 | 33 | | 38 | 18.6 | | 21.5 |  |
| Notechis_scutatus_MV_Z77599 | *Notechis* | *scutatus* | Tiger Snake | Female | 99.5 | 85 | NA | 171 | 50 | 224 | 32 | | 37 | 18.4 | | 20.7 |  |
| Notechis_scutatus_MV_Z77600 | *Notechis* | *scutatus* | Tiger Snake | Female | 98 | 82.5 | NA | 172 |  | 175 | 32 | | 36 | 18.3 | | 20.6 |  |
| Notechis_scutatus_MV_D76365 | *Notechis* | *scutatus* | Tiger Snake | Male | 101.5 | 86 | 324.5 | 168 | 47 | 218 | 32 | | 37 | 18.7 | | 21.1 |  |
| Notechis_scutatus_MV_D76366 | *Notechis* | *scutatus* | Tiger Snake | Female |  |  | 435.4 | 177 | 48 | 228 | 34 | | 39 | 18.9 | | 21.7 |  |
| Pseudonaja_textilis_MZRC_10093 | *Pseudonaja* | *textilis* | Eastern Brown Snake | Female | 126 | 103 | 263.9 | 203 | 63 | 269 | 40 | | 46 | 19.4 | | 22.3 |  |
| Pseudonaja_textilis_MV_D76367 | *Pseudonaja* | *textilis* | Eastern Brown Snake | Male | 108.5 | 90 | 252.9 | 200 | 52 | 255 | 39 | | 45 | 19.2 | | 22.2 |  |
| Pseudonaja_textilis_MV_D76368 | *Pseudonaja* | *textilis* | Eastern Brown Snake | Female | 113 | 101 | 374.9 | 200 | 50 | 253 | 39 | | 45 | 19.2 | | 22.2 |  |
| Pseudonaja_textilis_MV_D76369 | *Pseudonaja* | *textilis* | Eastern Brown Snake | Female | 116.5 | 101 | 424.2 | 207 | 47 | 257 | 42 | | 47 | 20.0 | | 22.4 |  |

**Supplementary Table 2:** Table showcasing the scan data of each snake sampled in the dataset.

| **Date** | **Scan Title** | **Scan Time (min)** | **Tube Voltage (kV)** | **Tube Current (mA)** | **resolution (mm)** | **timing (msec)** | **images** | **filter** | **User** |
| --- | --- | --- | --- | --- | --- | --- | --- | --- | --- |
| 24-Jan-24 | NMV_D76367 | 15 | 70 | 300 | 52.5 | 500 | 1798 | none | Ammresh Ammresh; Jane Melville; Jay Black; ARC Fossil DP |
| 24-Jan-24 | NMV_D76368 | 15 | 70 | 300 | 55 | 500 | 1798 | none | Ammresh Ammresh; Jane Melville; Jay Black; ARC Fossil DP |
| 22-Feb-24 | NMV_Z77599_0-80% | 30 | 70 | 300 | 50 | 500 | 1798 | none | Ammresh Ammresh; Jane Melville; Jay Black; ARC Fossil DP |
| 22-Feb-24 | NMV_Z77599_80-85% | 10 | 70 | 300 | 9 | 500 | 1199 | none | Ammresh Ammresh; Jane Melville; Jay Black; ARC Fossil DP |
| 22-Feb-24 | NMV_Z77599_85-90% | 10 | 70 | 300 | 8 | 500 | 1199 | none | Ammresh Ammresh; Jane Melville; Jay Black; ARC Fossil DP |
| 22-Feb-24 | NMV_Z77599_90-95% | 10 | 70 | 300 | 7 | 500 | 1199 | none | Ammresh Ammresh; Jane Melville; Jay Black; ARC Fossil DP |
| 22-Feb-24 | NMV_Z77599_95-100% | 10 | 70 | 300 | 9 | 500 | 1199 | none | Ammresh Ammresh; Jane Melville; Jay Black; ARC Fossil DP |
| 22-Feb-24 | NMV_D76369 | 30 | 70 | 300 | 50 | 500 | 1798 | 0.1mm Cu | Ammresh Ammresh; Jane Melville; Jay Black; ARC Fossil DP |
| 22-Feb-24 | NMV_D76366_0-75% | 30 | 70 | 300 | 50 | 500 | 1798 | 0.1mm Cu | Ammresh Ammresh; Jane Melville; Jay Black; ARC Fossil DP |
| 22-Feb-24 | NMV_D76366_75-85% | 20 | 70 | 300 | 20 | 500 | 1199 | 0.1mm Cu | Ammresh Ammresh; Jane Melville; Jay Black; ARC Fossil DP |
| 22-Feb-24 | NMV_D76366_85-90% | 10 | 70 | 300 | 10 | 500 | 1199 | 0.1mm Cu | Ammresh Ammresh; Jane Melville; Jay Black; ARC Fossil DP |
| 22-Feb-24 | NMV_D76366_90-95% | 15 | 70 | 300 | 10 | 500 | 1798 | 0.1mm Cu | Ammresh Ammresh; Jane Melville; Jay Black; ARC Fossil DP |
| 22-Feb-24 | NMV_D76366_95-100% | 10 | 70 | 300 | 10 | 500 | 1199 | 0.1mm Cu | Ammresh Ammresh; Jane Melville; Jay Black; ARC Fossil DP |
| 1-Mar-24 | NMV_D76365 | 30 | 70 | 300 | 55 | 500 | 1799 | 0.1mm Cu | Ammresh Ammresh; Jane Melville; Jay Black; ARC Fossil DP |
| 1-Mar-24 | NMV_D76369 | 30 | 70 | 300 | 50 | 500 | 1799 | 0.1mm Cu | Ammresh Ammresh; Jane Melville; Jay Black; ARC Fossil DP |
| 26-Jun-24 | NMV_D77598 | 15 | 70 | 300 | 40 | 500 | 1799 | none | Ammresh Ammresh; Jane Melville; Jay Black; ARC Fossil DP |
| 20-Nov-24 | MZRC_10089 | 30 | 70 | 300 | 40 | 500 | 1799 | none | Ammresh Ammresh; Jane Melville; Jay Black; ARC Fossil DP |
| 20-Nov-24 | MZRC_10091 | 30 | 70 | 300 | 40 | 500 | 1799 | none | Ammresh Ammresh; Jane Melville; Jay Black; ARC Fossil DP |
| 20-Nov-24 | MZRC_10088 | 45 | 70 | 300 | 40 | 500 | 1799 | none | Ammresh Ammresh; Jane Melville; Jay Black; ARC Fossil DP |
| 20-Nov-24 | NMV_Z77600 | 45 | 70 | 300 | 40 | 500 | 1799 | none | Ammresh Ammresh; Jane Melville; Jay Black; ARC Fossil DP |
| 13-Mar-25 | NMV_Z77601 | 30 | 70 | 300 | 40 | 500 | 1799 | none | Ammresh Ammresh; Jane Melville; Jay Black; ARC Fossil DP |

**Supplementary Table 3**: Table describing the features, processes and laminae seen in typical snake vertebrae.

| Name | Description |
| --- | --- |
| Centropostzygapophyseal lamina (CPOL) | The CPOL connects the dorsolateral corner of the postzygapophyses to the neural spine. Starting from the postzygapophysis, it curves markedly before straightening up on an incline towards the centre, meeting the neural spine. |
| Centrum | The body of the vertebra, below the neural canal and between the condyle and the cotyle. |
| Condyle | The approximately hemispherical posterior articular surface of the centrum. |
| Cotyle | The anterior concavity of the centrum, for the reception of the condyle of the preceding vertebra. |
| Hypapophysis | A median ventral process, either spinelike, or sigmoid-shaped, compressed, or bulbous. It is found in the anterior vertebrae of all snakes but decreases in size and disappears along the vertebral column in some snake families. |
| Haemal keel | A two-pronged ventral process, appearing in the same place as the hypapophysis but in the caudal vertebrae of all snakes. There is a major blood vessel that runs through the keel. |
| Neural Canal | The wide canal in the middle of the centrum that houses the spinal cord |
| Neural Spine | A dorsal process, spine-like or plate-like, arising at the base of the zygosphene, spanning the length of the centrum and ending at the base of the zygantrum. |
| Prespinal Lamina (PRSL) | The PRSL develops on the base of the zygosphene, connecting the neural spine summit to its base anteriorly. |
| Postspinal Lamina (POSL) | The PRSL is on the opposite side of the PRSL, connecting the neural spine summit to its base posteriorly. |
| Prezygapophysis | One of a pair of anterior processes arising from near the junction of the neural arch and the centrum on each side, bearing articular facets facing upward. |
| Prezygapophysial Accessory Process | A spine-like process which, if present, projects laterally from just below the prezygapophysial articular facets, and is more or less continuous with the prezygapophysis. |
| Postzygapophysis | One of a pair of posterior processes bearing articular facets facing downward for the articulation with the prezygapophysis of the following vertebra. |
| Subcentral Foramen | Foramina located on either side of the ventral surface of the centrum. |
| Subcentral Ridge | Lamina located on the ventral surface of the centrum, running along the base of the hypapophysis. |
| Synapophysis | Process where the rib articulates with the vertebra. |
| Zygantrum | A mortise-like depression on the posterior part of the CPOL above the neural canal, articulating with the zygosphene of the following vertebra. |
| Zygosphene | A wedge-shaped anterior process above the neural canal, bearing two articular facets facing outward and downward for articulation with the zygantrum of the preceding vertebra. |

**Supplementary Table 4**: Table showing the location of each landmark placed on the vertebrae.

| Landmark no. | Location of landmark |
| --- | --- |
| 1 | Top of the cotyle |
| 2 | Left side of the cotyle, where it meets the centrum at the entrance of the neural canal |
| 3 | Right side (anterior view) of the cotyle, where it meets the centrum at the entrance of the neural canal |
| 4 | Left side (anterior view) where the zygosphene meets the centrum at the entrance of the neural canal |
| 5 | Right side (anterior view) where the zygosphene meets the centrum at the entrance of the neural canal |
| 6 | Right-hand (anterior view) corner of the zygosphene |
| 7 | Left-hand (anterior view) corner of the zygosphene |
| 8 | Left side (anterior view) at the end of the zygosphene, where it meets the centrum |
| 9 | Right side (anterior view) at the end of the zygosphene, where it meets the centrum |
| 10 | Left side (anterior view) middle of the back of the prezygapophysis, where it meets the centrum |
| 11 | Left side (anterior view) middle of the front of the prezygapophysis |
| 12 | Left side (anterior view) the front centre of the prezygapophysis, where the curvature of the prezygapophysis starts |
| 13 | Left side (anterior view) the back centre of the prezygapophysis, where the curvature of the prezygapophysis starts |
| 14 | Left side (anterior view) most distal end of the prezygapophyseal accessory process |
| 15 | Left side (anterior view) most distal end at the bottom of the synapophysis |
| 16 | Left side (anterior view) Relative to landmark 15, follow the curve at the bottom of the synapophysis |
| 17 | Left side (anterior view) at the dorsal region of the synapophysis, at the top of the curve |
| 18 | Left side (anterior view) at the anterior portion of the lateral foramen |
| 19 | Left side (anterior view) at the end of the postzygapophysis, where it meets the centrum |
| 20 | Left side (anterior view) the middle of the postzygapophysis, where the curvature is most prominent |
| 21 | Left side (anterior view) at the back in the centre of the postzygapophysis |
| 22 | Right side (anterior view) middle of the back of the prezygapophysis, where it meets the centrum |
| 23 | Right side (anterior view) middle of the front of the prezygapophysis |
| 24 | Right side (anterior view) the front centre of the prezygapophysis, where the curvature of the prezygapophysis starts |
| 25 | Right side (anterior view) the back centre of the prezygapophysis, where the curvature of the prezygapophysis starts |
| 26 | Right side (anterior view) most distal end of the prezygapophyseal accessory process |
| 27 | Right side (anterior view) most distal end at the bottom of the synapophysis |
| 28 | Right side (anterior view) Relative to landmark 15, follow the curve at the bottom of the synapophysis |
| 29 | Right side (anterior view) at the top of the synapophysis, at the top of the curve |
| 30 | Right side (anterior view) at the front of the lateral foramen |
| 31 | Right side (anterior view) at the end of the postzygapophysis, where it meets the centrum |
| 32 | Right side (anterior view) the middle of the postzygapophysis, where the curvature is most prominent |
| 33 | Right side (anterior view) at the back in the centre of the postzygapophysis |
| 34-59 | Semi-landmark curve starting from the base of the zygosphene, along the top of the neural spine and ending at the bottom of the neural spine at the start of the zygantrum |
| 60-80 | Semi-landmark curve of the hypapophyses, starying from the bottom of the cotyle, following the bottom of the hypapophyses towards the bottom end of the condyle |

**Supplementary Table 5**: The results of the output from *MorphoRegions::modelsupport* tested with a maximum of 6 regions. This table showcases their breakpoints, sum of residual scores (SumRSS), Akaike Information Criterion (AICc), deltaAICc model likelihood and Akaike weight values.

| **Snake** | **regions** | **breakpoint1** | **breakpoint2** | **breakpoint3** | **breakpoint4** | **breakpoint5** | **sumRSS** | **AICc** | **deltaAIC** | **model_lik** | **Ak_weight** |
| --- | --- | --- | --- | --- | --- | --- | --- | --- | --- | --- | --- |
| Austrelaps_superbus_MZRC_10088 | 6 | 6 | 30 | 74 | 146 | 154 | 0.00791 | -4728.95 | 0.00 | 1 | 1 |
| Austrelaps_superbus_MZRC_10088 | 5 | 6 | 30 | 74 | 149 | - | 0.01125 | -4590.16 | 138.80 | 7.25E-31 | 7.25E-31 |
| Austrelaps_superbus_MZRC_10088 | 4 | 7 | 41 | 149 | - | - | 0.01644 | -4439.26 | 289.69 | 1.24E-63 | 1.24E-63 |
| Austrelaps_superbus_MZRC_10088 | 3 | 41 | 149 | - | - | - | 0.02286 | -4309.61 | 419.35 | 8.72E-92 | 8.72E-92 |
| Austrelaps_superbus_MZRC_10088 | 2 | 38 | - | - | - | - | 0.07792 | -3783.89 | 945.06 | 6.07E-206 | 6.07E-206 |
| Austrelaps_superbus_MZRC_10088 | 1 | - | - | - | - | - | 0.22136 | -3338.10 | 1390.86 | 9.53E-303 | 9.53E-303 |
| Austrelaps_superbus_MV_Z77598 | 6 | 7 | 32 | 72 | 135 | 152 | 0.00838 | -4989.22 | 0.00 | 1 | 1 |
| Austrelaps_superbus_MV_Z77598 | 5 | 7 | 32 | 72 | 150 | - | 0.01204 | -4837.77 | 151.45 | 1.30E-33 | 1.30E-33 |
| Austrelaps_superbus_MV_Z77598 | 4 | 7 | 40 | 150 | - | - | 0.01809 | -4664.41 | 324.80 | 2.95E-71 | 2.95E-71 |
| Austrelaps_superbus_MV_Z77598 | 3 | 36 | 150 | - | - | - | 0.03308 | -4399.30 | 589.91 | 7.98E-129 | 7.98E-129 |
| Austrelaps_superbus_MV_Z77598 | 2 | 31 | - | - | - | - | 0.08453 | -3978.08 | 1011.14 | 2.71E-220 | 2.71E-220 |
| Austrelaps_superbus_MV_Z77598 | 1 | - | - | - | - | - | 0.19890 | -3594.82 | 1394.40 | 1.62E-303 | 1.62E-303 |
| Notechis_scutatus_MZRC_10089 | 6 | 11 | 32 | 102 | 157 | 169 | 0.01044 | -5310.67 | 0.00 | 1 | 1 |
| Notechis_scutatus_MZRC_10089 | 5 | 12 | 33 | 157 | 169 | - | 0.01337 | -5203.02 | 107.65 | 4.21E-24 | 4.21E-24 |
| Notechis_scutatus_MZRC_10089 | 4 | 12 | 33 | 157 | - | - | 0.01743 | -5086.03 | 224.63 | 1.66E-49 | 1.66E-49 |
| Notechis_scutatus_MZRC_10089 | 3 | 33 | 157 | - | - | - | 0.03363 | -4772.31 | 538.35 | 1.25E-117 | 1.25E-117 |
| Notechis_scutatus_MZRC_10089 | 2 | 37 | - | - | - | - | 0.14363 | -4059.95 | 1250.71 | 2.58E-272 | 2.58E-272 |
| Notechis_scutatus_MZRC_10089 | 1 | - | - | - | - | - | 0.32845 | -3660.15 | 1650.52 | 0 | 0 |
| Notechis_scutatus_MZRC_10091 | 6 | 11 | 33 | 51 | 164 | 175 | 0.01123 | -5520.59 | 0.00 | 1 | 1 |
| Notechis_scutatus_MZRC_10091 | 5 | 12 | 48 | 164 | 175 | - | 0.01472 | -5395.87 | 124.71 | 8.29E-28 | 8.29E-28 |
| Notechis_scutatus_MZRC_10091 | 4 | 12 | 48 | 172 | - | - | 0.02284 | -5182.28 | 338.30 | 3.46E-74 | 3.46E-74 |
| Notechis_scutatus_MZRC_10091 | 3 | 22 | 171 | - | - | - | 0.04310 | -4866.17 | 654.41 | 7.87E-143 | 7.87E-143 |
| Notechis_scutatus_MZRC_10091 | 2 | 39 | - | - | - | - | 0.14057 | -4263.99 | 1256.59 | 1.36E-273 | 1.36E-273 |
| Notechis_scutatus_MZRC_10091 | 1 | - | - | - | - | - | 0.26518 | -3947.21 | 1573.37 | 0 | 0 |
| Notechis_scutatus_MV_D76365 | 6 | 11 | 43 | 106 | 154 | 167 | 0.00680 | -3379.11 | 0.00 | 1 | 1 |
| Notechis_scutatus_MV_D76365 | 5 | 11 | 43 | 106 | 158 | - | 0.00846 | -3320.84 | 58.26 | 2.23E-13 | 2.23E-13 |
| Notechis_scutatus_MV_D76365 | 4 | 13 | 98 | 158 | - | - | 0.01263 | -3204.22 | 174.89 | 1.05E-38 | 1.05E-38 |
| Notechis_scutatus_MV_D76365 | 3 | 13 | 151 | - | - | - | 0.02506 | -2996.20 | 382.91 | 7.12E-84 | 7.12E-84 |
| Notechis_scutatus_MV_D76365 | 2 | 17 | - | - | - | - | 0.06429 | -2705.47 | 673.63 | 5.28E-147 | 5.28E-147 |
| Notechis_scutatus_MV_D76365 | 1 | - | - | - | - | - | 0.17993 | -2386.59 | 992.52 | 3.01E-216 | 3.01E-216 |
| Notechis_scutatus_MV_D76366 | 6 | 14 | 32 | 82 | 158 | 173 | 0.00629 | -3689.11 | 0.00 | 1 | 1 |
| Notechis_scutatus_MV_D76366 | 5 | 14 | 32 | 88 | 166 | - | 0.00985 | -3546.94 | 142.16 | 1.35E-31 | 1.35E-31 |
| Notechis_scutatus_MV_D76366 | 4 | 14 | 32 | 163 | - | - | 0.01632 | -3384.59 | 304.52 | 7.49E-67 | 7.49E-67 |
| Notechis_scutatus_MV_D76366 | 3 | 26 | 162 | - | - | - | 0.02482 | -3251.43 | 437.68 | 9.12E-96 | 9.12E-96 |
| Notechis_scutatus_MV_D76366 | 2 | 155 | - | - | - | - | 0.14974 | -2643.86 | 1045.25 | 1.06E-227 | 1.06E-227 |
| Notechis_scutatus_MV_D76366 | 1 | - | - | - | - | - | 0.32421 | -2388.53 | 1300.58 | 3.83E-283 | 3.83E-283 |
| Notechis_scutatus_MV_Z77599 | 6 | 6 | 32 | 72 | 160 | 169 | 0.01420 | -4709.96 | 0.00 | 1 | 1 |
| Notechis_scutatus_MV_Z77599 | 5 | 6 | 32 | 72 | 165 | - | 0.01808 | -4614.93 | 95.02 | 2.32E-21 | 2.32E-21 |
| Notechis_scutatus_MV_Z77599 | 4 | 7 | 38 | 165 | - | - | 0.02243 | -4531.46 | 178.50 | 1.74E-39 | 1.74E-39 |
| Notechis_scutatus_MV_Z77599 | 3 | 35 | 165 | - | - | - | 0.03444 | -4348.98 | 360.98 | 4.12E-79 | 4.12E-79 |
| Notechis_scutatus_MV_Z77599 | 2 | 160 | - | - | - | - | 0.13413 | -3735.94 | 974.02 | 3.12E-212 | 3.12E-212 |
| Notechis_scutatus_MV_Z77599 | 1 | - | - | - | - | - | 0.26344 | -3438.72 | 1271.24 | 8.98E-277 | 8.98E-277 |
| Notechis_scutatus_MV_Z77600 | 6 | 13 | 33 | 56 | 82 | 161 | 0.00965 | -3244.05 | 0.00 | 1 | 1 |
| Notechis_scutatus_MV_Z77600 | 5 | 13 | 33 | 73 | 161 | - | 0.01243 | -3175.41 | 68.64 | 1.25E-15 | 1.25E-15 |
| Notechis_scutatus_MV_Z77600 | 4 | 38 | 73 | 161 | - | - | 0.01727 | -3082.52 | 161.53 | 8.38E-36 | 8.38E-36 |
| Notechis_scutatus_MV_Z77600 | 3 | 39 | 161 | - | - | - | 0.02263 | -3007.72 | 236.33 | 4.80E-52 | 4.80E-52 |
| Notechis_scutatus_MV_Z77600 | 2 | 43 | - | - | - | - | 0.10050 | -2544.40 | 699.66 | 1.18E-152 | 1.18E-152 |
| Notechis_scutatus_MV_Z77600 | 1 | - | - | - | - | - | 0.29457 | -2212.88 | 1031.18 | 1.21E-224 | 1.21E-224 |
| Pseudonaja_textilis_MZRC_10093 | 6 | 8 | 41 | 101 | 191 | 203 | 0.01464 | -6005.69 | 0.00 | 1 | 1 |
| Pseudonaja_textilis_MZRC_10093 | 5 | 8 | 41 | 101 | 200 | - | 0.02078 | -5820.15 | 185.55 | 5.12E-41 | 5.12E-41 |
| Pseudonaja_textilis_MZRC_10093 | 4 | 8 | 53 | 200 | - | - | 0.02910 | -5641.59 | 364.10 | 8.63E-80 | 8.63E-80 |
| Pseudonaja_textilis_MZRC_10093 | 3 | 34 | 200 | - | - | - | 0.05833 | -5256.37 | 749.32 | 1.93E-163 | 1.93E-163 |
| Pseudonaja_textilis_MZRC_10093 | 2 | 32 | - | - | - | - | 0.12413 | -4836.27 | 1169.42 | 1.16E-254 | 1.16E-254 |
| Pseudonaja_textilis_MZRC_10093 | 1 | - | - | - | - | - | 0.30070 | -4341.11 | 1664.58 | 0 | 0 |
| Pseudonaja_textilis_MV_D76367 | 6 | 9 | 39 | 85 | 177 | 198 | 0.01130 | -5977.37 | 0.00 | 1 | 1 |
| Pseudonaja_textilis_MV_D76367 | 5 | 9 | 39 | 85 | 195 | - | 0.01535 | -5821.41 | 155.96 | 1.36E-34 | 1.36E-34 |
| Pseudonaja_textilis_MV_D76367 | 4 | 9 | 52 | 194 | - | - | 0.02739 | -5512.35 | 465.02 | 1.05E-101 | 1.05E-101 |
| Pseudonaja_textilis_MV_D76367 | 3 | 42 | 194 | - | - | - | 0.05740 | -5112.56 | 864.82 | 1.61E-188 | 1.61E-188 |
| Pseudonaja_textilis_MV_D76367 | 2 | 42 | - | - | - | - | 0.12058 | -4711.05 | 1266.32 | 1.05E-275 | 1.05E-275 |
| Pseudonaja_textilis_MV_D76367 | 1 | - | - | - | - | - | 0.27153 | -4270.20 | 1707.18 | 0 | 0 |
| Pseudonaja_textilis_MV_D76368 | 6 | 8 | 41 | 97 | 195 | 201 | 0.01194 | -5593.86 | 0.00 | 1 | 1 |
| Pseudonaja_textilis_MV_D76368 | 5 | 8 | 41 | 95 | 197 | - | 0.01526 | -5479.93 | 113.92 | 1.83E-25 | 1.83E-25 |
| Pseudonaja_textilis_MV_D76368 | 4 | 40 | 97 | 197 | - | - | 0.02017 | -5347.57 | 246.28 | 3.31E-54 | 3.31E-54 |
| Pseudonaja_textilis_MV_D76368 | 3 | 41 | 197 | - | - | - | 0.02948 | -5161.58 | 432.28 | 1.36E-94 | 1.36E-94 |
| Pseudonaja_textilis_MV_D76368 | 2 | 41 | - | - | - | - | 0.10545 | -4499.69 | 1094.17 | 2.54E-238 | 2.54E-238 |
| Pseudonaja_textilis_MV_D76368 | 1 | - | - | - | - | - | 0.24025 | -4076.99 | 1516.87 | 0 | 0 |
| Pseudonaja_textilis_MV_D76369 | 6 | 9 | 52 | 106 | 182 | 199 | 0.01498 | -6374.99 | 0.00 | 1 | 1 |
| Pseudonaja_textilis_MV_D76369 | 5 | 9 | 52 | 106 | 199 | - | 0.01955 | -6228.91 | 146.08 | 1.91E-32 | 1.91E-32 |
| Pseudonaja_textilis_MV_D76369 | 4 | 9 | 52 | 202 | - | - | 0.02887 | -6007.00 | 367.99 | 1.24E-80 | 1.24E-80 |
| Pseudonaja_textilis_MV_D76369 | 3 | 48 | 202 | - | - | - | 0.05071 | -5679.22 | 695.77 | 8.23E-152 | 8.23E-152 |
| Pseudonaja_textilis_MV_D76369 | 2 | 48 | - | - | - | - | 0.22839 | -4777.49 | 1597.50 | 0 | 0 |
| Pseudonaja_textilis_MV_D76369 | 1 | - | - | - | - | - | 0.42882 | -4408.31 | 1966.68 | 0 | 0 |

**Supplementary Table 6**: Table showing the weighted mean breakpoints and weighted standard deviations for each region, averaged for each species. On the left we have the values for 6 regions and on the right the values for the 5 region model. Weighted standard deviations correspond to the variability in each breakpoint. All values were calculated using *Morphoregions::Bpvar*

|  | **6 regions** | | |  | **5 regions** | | |
| --- | --- | --- | --- | --- | --- | --- | --- |
|  | breakpoints | Mean | Standard Deviation |  | breakpoints | Mean | Standard Deviation |
| *Austrelaps superbus* | breakpoint1 | 6.737792856 | 0.25971347 |  | breakpoint1 | 6.873988859 | 0.324817195 |
|  | breakpoint2 | 31.00180317 | 0.118074748 |  | breakpoint2 | 31.03514863 | 0.207812334 |
|  | breakpoint3 | 72.98620142 | 0.213030976 |  | breakpoint3 | 73.03604988 | 0.448982505 |
|  | breakpoint4 | 140.2479774 | 0.906305012 |  | breakpoint4 | 149.187937 | 0.509368959 |
|  | breakpoint5 | 153.0369194 | 0.141694339 |  |  |  |  |
| *Notechis scutatus* | breakpoint1 | 11.06978759 | 0.733890288 |  | breakpoint1 | 10.92288216 | 0.778929989 |
|  | breakpoint2 | 34.37852251 | 0.685355122 |  | breakpoint2 | 35.12378108 | 0.665518153 |
|  | breakpoint3 | 80.84346037 | 4.63693749 |  | breakpoint3 | 122.7406954 | 1.211646943 |
|  | breakpoint4 | 145.4508004 | 1.072200849 |  | breakpoint4 | 164.6490959 | 1.138552336 |
|  | breakpoint5 | 168.7651203 | 0.381945784 |  |  |  |  |
| *Pseudonaja textilis* | breakpoint1 | 8.938429303 | 0.519388753 |  | breakpoint1 | 8.958800917 | 0.419452863 |
|  | breakpoint2 | 43.24934857 | 0.838969659 |  | breakpoint2 | 42.96391214 | 1.085376304 |
|  | breakpoint3 | 97.44830382 | 0.638941597 |  | breakpoint3 | 97.57946788 | 0.652434156 |
|  | breakpoint4 | 186.4456471 | 2.505016979 |  | breakpoint4 | 197.9387287 | 0.547869371 |
|  | breakpoint5 | 201.3227721 | 0.23214832 |  |  |  |  |

**Supplementary Table 7**: The table shows the average number of vertebrae in each region of the chosen 5-region model, along with the percentage length each region occupies within the pre-cloacal vertebral column, for each species.

| Species | Average vertebral number | Region | | | | |
| --- | --- | --- | --- | --- | --- | --- |
|  |  | Cervical | Anterior Thoracic | Middle Thoracic | Posterior Thoracic | Lumbar |
|  |  | Percentage | Percentage | Percentage | Percentage | Percentage |
| *Austrelaps superbus* | 154 | 2.92% | 15.58% | 27.60% | 49.68% | 4.22% |
| *Notechis scutatus* | 172 | 3.97% | 14.33% | 26.04% | 51.21% | 4.45% |
| *Pseudonaja textilis* | 203 | 3.33% | 16.67% | 27.04% | 49.88% | 3.09% |

**Supplementary Table 8:** Linear regression results testing the relationship between heart position and vertebral regionalisation in elapid snakes. The table shows parameter estimates from a linear model relating heart midpoint position to the second vertebral breakpoint (bp2) along the pre-caudal column. Breakpoint position increases at a slope of 0.95 (p = 0.001), indicating that the anterior thoracic regional boundary closely tracks heart placement across species.

|  | Estimate | Std. Error | t value | Pr(>\|t\|) |
| --- | --- | --- | --- | --- |
| (Intercept) | 2.215 | 6.916 | 0.320 | 0.755 |
| heart_mid | 0.949 | 0.204 | 4.645 | 0.001 |

**Supplementary Table 9**: The results of the output from *MorphoRegions::modelsupport* tested with 5 regions on 4 different subsamples (vertebrae sampled at every 5%, 4%, 2.5% and 2%). This table showcases their breakpoints, sum of residual scores, AICc, deltaAICc model likelihood and Akaike weight values.

| **Snake** | **Sampling** | **regions** | **breakpoint1** | **breakpoint2** | **breakpoint3** | **breakpoint4** | **sumRSS** | **AICc** | **deltaAIC** | **model_lik** | **Ak_weight** |
| --- | --- | --- | --- | --- | --- | --- | --- | --- | --- | --- | --- |
| Austrelaps_superbus_MZRC_10088 | 5% | 4 | 11 | 26 | 141 | - | 0.000635 | -167.202244 | 0 | 1 | 0.986010965 |
| Austrelaps_superbus_MZRC_10088 | 5% | 3 | 34 | 141 | - | - | 0.003022 | -157.773212 | 9.429032597 | 0.008964201 | 0.0088388 |
| Austrelaps_superbus_MZRC_10088 | 5% | 2 | 26 | - | - | - | 0.006227 | -156.590133 | 10.61211105 | 0.004961459 | 0.004892053 |
| Austrelaps_superbus_MZRC_10088 | 5% | 5 | 11 | 26 | 73 | 141 | 0.000151 | -150.706602 | 16.49564194 | 0.000261828 | 0.000258166 |
| Austrelaps_superbus_MZRC_10088 | 5% | 1 | - | - | - | - | 0.032350 | -131.322562 | 35.87968213 | 1.62E-08 | 1.59E-08 |
| Austrelaps_superbus_MZRC_10088 | 4% | 5 | 10 | 27 | 71 | 144 | 0.000247 | -234.462977 | 0 | 1 | 0.877092012 |
| Austrelaps_superbus_MZRC_10088 | 4% | 4 | 10 | 34 | 144 | - | 0.000762 | -230.532213 | 3.930764068 | 0.140102353 | 0.122882654 |
| Austrelaps_superbus_MZRC_10088 | 4% | 3 | 34 | 144 | - | - | 0.002752 | -213.523281 | 20.93969638 | 2.84E-05 | 2.49E-05 |
| Austrelaps_superbus_MZRC_10088 | 4% | 2 | 34 | - | - | - | 0.005833 | -205.461477 | 29.00150005 | 5.04E-07 | 4.42E-07 |
| Austrelaps_superbus_MZRC_10088 | 4% | 1 | - | - | - | - | 0.031263 | -170.287240 | 64.17573678 | 1.16E-14 | 1.02E-14 |
| Austrelaps_superbus_MZRC_10088 | 2.5% | 5 | 8 | 26 | 78 | 145 | 0.000775 | -401.762138 | 0 | 1 | 0.996841397 |
| Austrelaps_superbus_MZRC_10088 | 2.5% | 4 | 8 | 26 | 145 | - | 0.001411 | -390.253207 | 11.5089304 | 0.003168601 | 0.003158592 |
| Austrelaps_superbus_MZRC_10088 | 2.5% | 3 | 38 | 145 | - | - | 0.003373 | -365.126762 | 36.63537551 | 1.11E-08 | 1.10E-08 |
| Austrelaps_superbus_MZRC_10088 | 2.5% | 2 | 34 | - | - | - | 0.006232 | -348.744492 | 53.01764574 | 3.07E-12 | 3.06E-12 |
| Austrelaps_superbus_MZRC_10088 | 2.5% | 1 | - | - | - | - | 0.037211 | -282.877644 | 118.8844932 | 1.53E-26 | 1.52E-26 |
| Austrelaps_superbus_MZRC_10088 | 2% | 5 | 7 | 27 | 74 | 147 | 0.000831 | -522.579658 | 0 | 1 | 0.999988598 |
| Austrelaps_superbus_MZRC_10088 | 2% | 4 | 7 | 27 | 147 | - | 0.001608 | -499.816273 | 22.76338437 | 1.14E-05 | 1.14E-05 |
| Austrelaps_superbus_MZRC_10088 | 2% | 3 | 37 | 147 | - | - | 0.003083 | -475.965676 | 46.61398125 | 7.55E-11 | 7.55E-11 |
| Austrelaps_superbus_MZRC_10088 | 2% | 2 | 34 | - | - | - | 0.005927 | -450.732460 | 71.84719703 | 2.50E-16 | 2.50E-16 |
| Austrelaps_superbus_MZRC_10088 | 2% | 1 | - | - | - | - | 0.039310 | -361.322672 | 161.256986 | 9.63E-36 | 9.63E-36 |
| Austrelaps_superbus_MV_Z77598 | 5% | 4 | 12 | 27 | 135 | - | 0.000595 | -168.559081 | 0 | 1 | 0.469645303 |
| Austrelaps_superbus_MV_Z77598 | 5% | 3 | 12 | 27 | - | - | 0.001846 | -168.119387 | 0.439694528 | 0.802641381 | 0.376956755 |
| Austrelaps_superbus_MV_Z77598 | 5% | 2 | 19 | - | - | - | 0.003931 | -166.249460 | 2.309620849 | 0.31511727 | 0.147993346 |
| Austrelaps_superbus_MV_Z77598 | 5% | 5 | 12 | 27 | 66 | 143 | 0.000099 | -159.629623 | 8.929458761 | 0.01150781 | 0.005404589 |
| Austrelaps_superbus_MV_Z77598 | 5% | 1 | - | - | - | - | 0.030532 | -132.537149 | 36.02193233 | 1.51E-08 | 7.07E-09 |
| Austrelaps_superbus_MV_Z77598 | 4% | 4 | 10 | 29 | 78 | - | 0.000797 | -229.361129 | 0 | 1 | 0.780035194 |
| Austrelaps_superbus_MV_Z77598 | 4% | 5 | 10 | 29 | 72 | 127 | 0.000337 | -226.404120 | 2.957009419 | 0.227978328 | 0.177831119 |
| Austrelaps_superbus_MV_Z77598 | 4% | 3 | 10 | 29 | - | - | 0.001873 | -223.519827 | 5.841302145 | 0.053898584 | 0.042042792 |
| Austrelaps_superbus_MV_Z77598 | 4% | 2 | 16 | - | - | - | 0.004669 | -211.246352 | 18.11477712 | 0.000116527 | 9.09E-05 |
| Austrelaps_superbus_MV_Z77598 | 4% | 1 | - | - | - | - | 0.031174 | -170.361625 | 58.99950477 | 1.54E-13 | 1.20E-13 |
| Austrelaps_superbus_MV_Z77598 | 2.5% | 5 | 7 | 31 | 69 | 127 | 0.000578 | -413.772335 | 0 | 1 | 0.999712911 |
| Austrelaps_superbus_MV_Z77598 | 2.5% | 4 | 7 | 39 | 131 | - | 0.001184 | -397.461472 | 16.31086271 | 0.000287171 | 0.000287089 |
| Austrelaps_superbus_MV_Z77598 | 2.5% | 3 | 7 | 31 | - | - | 0.003176 | -367.596816 | 46.17551905 | 9.40E-11 | 9.40E-11 |
| Austrelaps_superbus_MV_Z77598 | 2.5% | 2 | 27 | - | - | - | 0.006297 | -348.320333 | 65.45200171 | 6.13E-15 | 6.13E-15 |
| Austrelaps_superbus_MV_Z77598 | 2.5% | 1 | - | - | - | - | 0.036506 | -283.662398 | 130.1099374 | 5.58E-29 | 5.58E-29 |
| Austrelaps_superbus_MV_Z77598 | 2% | 5 | 6 | 32 | 72 | 136 | 0.000793 | -525.007474 | 0 | 1 | 0.999951961 |
| Austrelaps_superbus_MV_Z77598 | 2% | 4 | 6 | 32 | 78 | - | 0.001449 | -505.120586 | 19.88688882 | 4.80E-05 | 4.80E-05 |
| Austrelaps_superbus_MV_Z77598 | 2% | 3 | 6 | 32 | - | - | 0.003673 | -467.043298 | 57.96417633 | 2.59E-13 | 2.59E-13 |
| Austrelaps_superbus_MV_Z77598 | 2% | 2 | 22 | - | - | - | 0.007714 | -437.291780 | 87.7156945 | 8.97E-20 | 8.97E-20 |
| Austrelaps_superbus_MV_Z77598 | 2% | 1 | - | - | - | - | 0.040339 | -360.005647 | 165.0018279 | 1.48E-36 | 1.48E-36 |
| Notechis_scutatus_MZRC_10089 | 5% | 4 | 12 | 38 | 155 | - | 0.000406 | -176.587550 | 0 | 1 | 0.999582367 |
| Notechis_scutatus_MZRC_10089 | 5% | 5 | 12 | 30 | 97 | 155 | 0.000096 | -160.122503 | 16.46504661 | 0.000265865 | 0.000265754 |
| Notechis_scutatus_MZRC_10089 | 5% | 3 | 12 | 155 | - | - | 0.002850 | -159.003498 | 17.58405207 | 0.00015194 | 0.000151876 |
| Notechis_scutatus_MZRC_10089 | 5% | 2 | 12 | - | - | - | 0.015548 | -137.374602 | 39.21294803 | 3.06E-09 | 3.05E-09 |
| Notechis_scutatus_MZRC_10089 | 5% | 1 | - | - | - | - | 0.062774 | -117.400717 | 59.1868329 | 1.41E-13 | 1.40E-13 |
| Notechis_scutatus_MZRC_10089 | 4% | 4 | 11 | 38 | 159 | - | 0.000488 | -242.088132 | 0 | 1 | 0.993998729 |
| Notechis_scutatus_MZRC_10089 | 4% | 5 | 11 | 25 | 99 | 159 | 0.000273 | -231.868602 | 10.2195299 | 0.006037502 | 0.006001269 |
| Notechis_scutatus_MZRC_10089 | 4% | 3 | 11 | 159 | - | - | 0.004337 | -201.692454 | 40.39567835 | 1.69E-09 | 1.68E-09 |
| Notechis_scutatus_MZRC_10089 | 4% | 2 | 17 | - | - | - | 0.016037 | -179.163986 | 62.92414583 | 2.17E-14 | 2.16E-14 |
| Notechis_scutatus_MZRC_10089 | 4% | 1 | - | - | - | - | 0.064092 | -151.622142 | 90.46599039 | 2.27E-20 | 2.25E-20 |
| Notechis_scutatus_MZRC_10089 | 2.5% | 5 | 8 | 30 | 101 | 160 | 0.000620 | -410.943218 | 0 | 1 | 0.99808606 |
| Notechis_scutatus_MZRC_10089 | 2.5% | 4 | 8 | 30 | 160 | - | 0.001156 | -398.429867 | 12.51335142 | 0.00191761 | 0.00191394 |
| Notechis_scutatus_MZRC_10089 | 2.5% | 3 | 16 | 155 | - | - | 0.008393 | -327.751283 | 83.1919355 | 8.61E-19 | 8.60E-19 |
| Notechis_scutatus_MZRC_10089 | 2.5% | 2 | 34 | - | - | - | 0.019053 | -302.923814 | 108.0194039 | 3.50E-24 | 3.49E-24 |
| Notechis_scutatus_MZRC_10089 | 2.5% | 1 | - | - | - | - | 0.072760 | -255.384876 | 155.558342 | 1.66E-34 | 1.66E-34 |
| Notechis_scutatus_MZRC_10089 | 2% | 5 | 7 | 32 | 102 | 159 | 0.000817 | -523.470207 | 0 | 1 | 0.996393036 |
| Notechis_scutatus_MZRC_10089 | 2% | 4 | 7 | 32 | 159 | - | 0.001261 | -512.227656 | 11.24255094 | 0.003620021 | 0.003606964 |
| Notechis_scutatus_MZRC_10089 | 2% | 3 | 17 | 159 | - | - | 0.009183 | -420.303778 | 103.1664294 | 3.96E-23 | 3.95E-23 |
| Notechis_scutatus_MZRC_10089 | 2% | 2 | 32 | - | - | - | 0.022006 | -383.827686 | 139.6425209 | 4.75E-31 | 4.74E-31 |
| Notechis_scutatus_MZRC_10089 | 2% | 1 | - | - | - | - | 0.085671 | -321.592399 | 201.8778077 | 1.45E-44 | 1.45E-44 |
| Notechis_scutatus_MZRC_10091 | 5% | 3 | 13 | 161 | - | - | 0.001412 | -173.758805 | 0 | 1 | 0.552794509 |
| Notechis_scutatus_MZRC_10091 | 5% | 4 | 13 | 39 | 161 | - | 0.000474 | -173.334867 | 0.423937973 | 0.808989787 | 0.447205112 |
| Notechis_scutatus_MZRC_10091 | 5% | 5 | 13 | 30 | 47 | 161 | 0.000201 | -144.723272 | 29.03553305 | 4.95E-07 | 2.74E-07 |
| Notechis_scutatus_MZRC_10091 | 5% | 2 | 13 | - | - | - | 0.012001 | -142.813633 | 30.94517209 | 1.91E-07 | 1.05E-07 |
| Notechis_scutatus_MZRC_10091 | 5% | 1 | - | - | - | - | 0.040377 | -126.667537 | 47.09126772 | 5.95E-11 | 3.29E-11 |
| Notechis_scutatus_MZRC_10091 | 4% | 4 | 11 | 39 | 164 | - | 0.000705 | -232.527745 | 0 | 1 | 0.984383871 |
| Notechis_scutatus_MZRC_10091 | 4% | 5 | 11 | 32 | 52 | 164 | 0.000367 | -224.202008 | 8.325736677 | 0.015562854 | 0.015319823 |
| Notechis_scutatus_MZRC_10091 | 4% | 3 | 11 | 164 | - | - | 0.002472 | -216.310987 | 16.21675763 | 0.000301006 | 0.000296306 |
| Notechis_scutatus_MZRC_10091 | 4% | 2 | 11 | - | - | - | 0.011226 | -188.437408 | 44.09033631 | 2.67E-10 | 2.62E-10 |
| Notechis_scutatus_MZRC_10091 | 4% | 1 | - | - | - | - | 0.037143 | -165.806320 | 66.72142504 | 3.25E-15 | 3.20E-15 |
| Notechis_scutatus_MZRC_10091 | 2.5% | 5 | 13 | 30 | 135 | 165 | 0.000802 | -400.362626 | 0 | 1 | 0.961296815 |
| Notechis_scutatus_MZRC_10091 | 2.5% | 4 | 13 | 30 | 165 | - | 0.001290 | -393.937903 | 6.4247229 | 0.040261426 | 0.03870318 |
| Notechis_scutatus_MZRC_10091 | 2.5% | 3 | 17 | 165 | - | - | 0.003612 | -362.321785 | 38.04084145 | 5.49E-09 | 5.28E-09 |
| Notechis_scutatus_MZRC_10091 | 2.5% | 2 | 21 | - | - | - | 0.012316 | -320.813667 | 79.54895939 | 5.32E-18 | 5.12E-18 |
| Notechis_scutatus_MZRC_10091 | 2.5% | 1 | - | - | - | - | 0.044894 | -275.181975 | 125.1806512 | 6.57E-28 | 6.31E-28 |
| Notechis_scutatus_MZRC_10091 | 2% | 5 | 11 | 32 | 49 | 171 | 0.001266 | -501.113904 | 0 | 1 | 0.984289092 |
| Notechis_scutatus_MZRC_10091 | 2% | 4 | 6 | 42 | 171 | - | 0.001844 | -492.838776 | 8.275128773 | 0.015961681 | 0.015710908 |
| Notechis_scutatus_MZRC_10091 | 2% | 3 | 14 | 171 | - | - | 0.005575 | -445.757256 | 55.35664787 | 9.54E-13 | 9.39E-13 |
| Notechis_scutatus_MZRC_10091 | 2% | 2 | 18 | - | - | - | 0.011443 | -417.177702 | 83.93620216 | 5.94E-19 | 5.84E-19 |
| Notechis_scutatus_MZRC_10091 | 2% | 1 | - | - | - | - | 0.042769 | -357.022551 | 144.0913536 | 5.14E-32 | 5.06E-32 |
| Notechis_scutatus_MV_D76365 | 5% | 4 | 11 | 102 | 154 | - | 0.000470 | -173.522937 | 0 | 1 | 0.916947352 |
| Notechis_scutatus_MV_D76365 | 5% | 3 | 11 | 146 | - | - | 0.001794 | -168.718583 | 4.80435403 | 0.090520674 | 0.083002692 |
| Notechis_scutatus_MV_D76365 | 5% | 2 | 11 | - | - | - | 0.007114 | -153.794574 | 19.7283626 | 5.20E-05 | 4.77E-05 |
| Notechis_scutatus_MV_D76365 | 5% | 5 | 11 | 36 | 76 | 154 | 0.000174 | -147.704837 | 25.81810018 | 2.48E-06 | 2.27E-06 |
| Notechis_scutatus_MV_D76365 | 5% | 1 | - | - | - | - | 0.039615 | -127.067631 | 46.45530616 | 8.17E-11 | 7.49E-11 |
| Notechis_scutatus_MV_D76365 | 4% | 4 | 9 | 102 | 158 | - | 0.001060 | -221.928735 | 0 | 1 | 0.936028385 |
| Notechis_scutatus_MV_D76365 | 4% | 5 | 9 | 42 | 102 | 158 | 0.000498 | -216.256732 | 5.672002886 | 0.058659752 | 0.054907193 |
| Notechis_scutatus_MV_D76365 | 4% | 3 | 9 | 151 | - | - | 0.002845 | -212.653896 | 9.274838583 | 0.009682654 | 0.009063239 |
| Notechis_scutatus_MV_D76365 | 4% | 2 | 9 | - | - | - | 0.008800 | -194.767441 | 27.16129383 | 1.26E-06 | 1.18E-06 |
| Notechis_scutatus_MV_D76365 | 4% | 1 | - | - | - | - | 0.041680 | -162.809794 | 59.11894086 | 1.45E-13 | 1.36E-13 |
| Notechis_scutatus_MV_D76365 | 2.5% | 5 | 7 | 40 | 84 | 163 | 0.000672 | -407.647565 | 0 | 1 | 0.999999875 |
| Notechis_scutatus_MV_D76365 | 2.5% | 4 | 11 | 84 | 163 | - | 0.002004 | -375.862529 | 31.7850363 | 1.25E-07 | 1.25E-07 |
| Notechis_scutatus_MV_D76365 | 2.5% | 3 | 11 | 97 | - | - | 0.003956 | -358.592676 | 49.05488902 | 2.23E-11 | 2.23E-11 |
| Notechis_scutatus_MV_D76365 | 2.5% | 2 | 16 | - | - | - | 0.011363 | -324.116952 | 83.53061378 | 7.27E-19 | 7.27E-19 |
| Notechis_scutatus_MV_D76365 | 2.5% | 1 | - | - | - | - | 0.046582 | -273.668540 | 133.9790258 | 8.07E-30 | 8.07E-30 |
| Notechis_scutatus_MV_D76365 | 2% | 5 | 9 | 42 | 77 | 165 | 0.001098 | -508.384011 | 0 | 1 | 0.999999673 |
| Notechis_scutatus_MV_D76365 | 2% | 4 | 9 | 98 | 165 | - | 0.002442 | -478.517810 | 29.86620142 | 3.27E-07 | 3.27E-07 |
| Notechis_scutatus_MV_D76365 | 2% | 3 | 9 | 88 | - | - | 0.004174 | -460.511676 | 47.87233534 | 4.02E-11 | 4.02E-11 |
| Notechis_scutatus_MV_D76365 | 2% | 2 | 13 | - | - | - | 0.012816 | -411.397930 | 96.98608177 | 8.70E-22 | 8.70E-22 |
| Notechis_scutatus_MV_D76365 | 2% | 1 | - | - | - | - | 0.050243 | -348.808472 | 159.5755391 | 2.23E-35 | 2.23E-35 |
| Notechis_scutatus_MV_D76366 | 5% | 3 | 12 | 89 | - | - | 0.001330 | -175.001499 | 0 | 1 | 0.699081766 |
| Notechis_scutatus_MV_D76366 | 5% | 4 | 12 | 29 | 144 | - | 0.000476 | -173.243360 | 1.758138665 | 0.415169116 | 0.290237159 |
| Notechis_scutatus_MV_D76366 | 5% | 2 | 20 | - | - | - | 0.003859 | -166.638551 | 8.362947608 | 0.015275977 | 0.010679157 |
| Notechis_scutatus_MV_D76366 | 5% | 5 | 12 | 29 | 81 | 144 | 0.000161 | -149.388528 | 25.61297055 | 2.74E-06 | 1.92E-06 |
| Notechis_scutatus_MV_D76366 | 5% | 1 | - | - | - | - | 0.032838 | -131.007542 | 43.99395648 | 2.80E-10 | 1.96E-10 |
| Notechis_scutatus_MV_D76366 | 4% | 4 | 10 | 37 | 144 | - | 0.000513 | -240.815113 | 0 | 1 | 0.726441349 |
| Notechis_scutatus_MV_D76366 | 4% | 5 | 10 | 30 | 79 | 158 | 0.000209 | -238.857964 | 1.957148431 | 0.375846593 | 0.273030506 |
| Notechis_scutatus_MV_D76366 | 4% | 3 | 17 | 102 | - | - | 0.001680 | -226.355884 | 14.45922922 | 0.0007248 | 0.000526525 |
| Notechis_scutatus_MV_D76366 | 4% | 2 | 24 | - | - | - | 0.004074 | -214.788699 | 26.02641418 | 2.23E-06 | 1.62E-06 |
| Notechis_scutatus_MV_D76366 | 4% | 1 | - | - | - | - | 0.032939 | -168.929153 | 71.88595976 | 2.46E-16 | 1.78E-16 |
| Notechis_scutatus_MV_D76366 | 2.5% | 5 | 12 | 29 | 85 | 170 | 0.000573 | -414.156214 | 0 | 1 | 0.999956709 |
| Notechis_scutatus_MV_D76366 | 2.5% | 4 | 12 | 29 | 152 | - | 0.001286 | -394.061184 | 20.09503008 | 4.33E-05 | 4.33E-05 |
| Notechis_scutatus_MV_D76366 | 2.5% | 3 | 24 | 152 | - | - | 0.003352 | -365.378817 | 48.77739709 | 2.56E-11 | 2.56E-11 |
| Notechis_scutatus_MV_D76366 | 2.5% | 2 | 29 | - | - | - | 0.007792 | -339.581560 | 74.57465418 | 6.40E-17 | 6.40E-17 |
| Notechis_scutatus_MV_D76366 | 2.5% | 1 | - | - | - | - | 0.050220 | -270.585528 | 143.5706859 | 6.67E-32 | 6.67E-32 |
| Notechis_scutatus_MV_D76366 | 2% | 5 | 10 | 30 | 79 | 151 | 0.001166 | -505.333587 | 0 | 1 | 0.999008843 |
| Notechis_scutatus_MV_D76366 | 2% | 4 | 10 | 30 | 151 | - | 0.001893 | -491.502294 | 13.8312931 | 0.00099214 | 0.000991156 |
| Notechis_scutatus_MV_D76366 | 2% | 3 | 20 | 93 | - | - | 0.004175 | -460.505285 | 44.8283014 | 1.84E-10 | 1.84E-10 |
| Notechis_scutatus_MV_D76366 | 2% | 2 | 30 | - | - | - | 0.008186 | -434.259502 | 71.07408455 | 3.69E-16 | 3.68E-16 |
| Notechis_scutatus_MV_D76366 | 2% | 1 | - | - | - | - | 0.055481 | -343.750739 | 161.5828481 | 8.18E-36 | 8.17E-36 |
| Notechis_scutatus_MV_Z77599 | 5% | 3 | 12 | 158 | - | - | 0.000959 | -181.871974 | 0 | 1 | 0.998382661 |
| Notechis_scutatus_MV_Z77599 | 5% | 4 | 12 | 33 | 158 | - | 0.000582 | -169.021256 | 12.85071728 | 0.001619952 | 0.001617332 |
| Notechis_scutatus_MV_Z77599 | 5% | 2 | 12 | - | - | - | 0.011199 | -144.264996 | 37.60697784 | 6.82E-09 | 6.81E-09 |
| Notechis_scutatus_MV_Z77599 | 5% | 5 | 12 | 33 | 80 | 158 | 0.000298 | -136.405692 | 45.46628149 | 1.34E-10 | 1.34E-10 |
| Notechis_scutatus_MV_Z77599 | 5% | 1 | - | - | - | - | 0.048238 | -122.932250 | 58.93972373 | 1.59E-13 | 1.59E-13 |
| Notechis_scutatus_MV_Z77599 | 4% | 4 | 10 | 34 | 162 | - | 0.000698 | -232.796877 | 0 | 1 | 0.798166446 |
| Notechis_scutatus_MV_Z77599 | 4% | 5 | 10 | 34 | 78 | 162 | 0.000293 | -230.029605 | 2.767272382 | 0.250665427 | 0.200072733 |
| Notechis_scutatus_MV_Z77599 | 4% | 3 | 10 | 162 | - | - | 0.002099 | -220.563799 | 12.23307752 | 0.002206079 | 0.001760818 |
| Notechis_scutatus_MV_Z77599 | 4% | 2 | 18 | - | - | - | 0.008862 | -194.586257 | 38.2106197 | 5.04E-09 | 4.02E-09 |
| Notechis_scutatus_MV_Z77599 | 4% | 1 | - | - | - | - | 0.044425 | -161.151528 | 71.64534873 | 2.77E-16 | 2.21E-16 |
| Notechis_scutatus_MV_Z77599 | 2.5% | 5 | 7 | 41 | 80 | 166 | 0.000902 | -395.560118 | 0 | 1 | 0.99992888 |
| Notechis_scutatus_MV_Z77599 | 2.5% | 4 | 7 | 29 | 158 | - | 0.001975 | -376.457933 | 19.10218548 | 7.11E-05 | 7.11E-05 |
| Notechis_scutatus_MV_Z77599 | 2.5% | 3 | 12 | 158 | - | - | 0.004277 | -355.388917 | 40.17120188 | 1.89E-09 | 1.89E-09 |
| Notechis_scutatus_MV_Z77599 | 2.5% | 2 | 12 | - | - | - | 0.013954 | -315.694096 | 79.86602204 | 4.54E-18 | 4.54E-18 |
| Notechis_scutatus_MV_Z77599 | 2.5% | 1 | - | - | - | - | 0.051725 | -269.374825 | 126.1852935 | 3.97E-28 | 3.97E-28 |
| Notechis_scutatus_MV_Z77599 | 2% | 5 | 6 | 34 | 81 | 168 | 0.001019 | -512.201826 | 0 | 1 | 0.999995624 |
| Notechis_scutatus_MV_Z77599 | 2% | 4 | 6 | 34 | 168 | - | 0.002047 | -487.523299 | 24.67852711 | 4.38E-06 | 4.38E-06 |
| Notechis_scutatus_MV_Z77599 | 2% | 3 | 18 | 168 | - | - | 0.005492 | -446.525135 | 65.67669086 | 5.48E-15 | 5.48E-15 |
| Notechis_scutatus_MV_Z77599 | 2% | 2 | 18 | - | - | - | 0.011679 | -416.137143 | 96.06468311 | 1.38E-21 | 1.38E-21 |
| Notechis_scutatus_MV_Z77599 | 2% | 1 | - | - | - | - | 0.050987 | -348.058960 | 164.142866 | 2.27E-36 | 2.27E-36 |
| Notechis_scutatus_MV_Z77600 | 5% | 4 | 13 | 31 | 147 | - | 0.000812 | -162.030466 | 0 | 1 | 0.860692229 |
| Notechis_scutatus_MV_Z77600 | 5% | 3 | 31 | 147 | - | - | 0.002936 | -158.377901 | 3.652564434 | 0.161011061 | 0.138580969 |
| Notechis_scutatus_MV_Z77600 | 5% | 2 | 39 | - | - | - | 0.009430 | -147.876102 | 14.15436323 | 0.000844149 | 0.000726552 |
| Notechis_scutatus_MV_Z77600 | 5% | 5 | 13 | 31 | 92 | 157 | 0.000370 | -131.887292 | 30.14317356 | 2.85E-07 | 2.45E-07 |
| Notechis_scutatus_MV_Z77600 | 5% | 1 | - | - | - | - | 0.046166 | -123.853842 | 38.17662321 | 5.13E-09 | 4.41E-09 |
| Notechis_scutatus_MV_Z77600 | 4% | 4 | 11 | 32 | 153 | - | 0.001176 | -219.234742 | 0 | 1 | 0.810268835 |
| Notechis_scutatus_MV_Z77600 | 4% | 5 | 11 | 32 | 72 | 153 | 0.000497 | -216.321139 | 2.913603465 | 0.232980217 | 0.188776609 |
| Notechis_scutatus_MV_Z77600 | 4% | 3 | 32 | 153 | - | - | 0.003711 | -205.746888 | 13.48785404 | 0.001178012 | 0.000954506 |
| Notechis_scutatus_MV_Z77600 | 4% | 2 | 39 | - | - | - | 0.012332 | -185.995584 | 33.23915811 | 6.06E-08 | 4.91E-08 |
| Notechis_scutatus_MV_Z77600 | 4% | 1 | - | - | - | - | 0.051136 | -157.493640 | 61.74110268 | 3.92E-14 | 3.17E-14 |
| Notechis_scutatus_MV_Z77600 | 2.5% | 5 | 7 | 31 | 76 | 157 | 0.001729 | -368.871388 | 0 | 1 | 0.913879343 |
| Notechis_scutatus_MV_Z77600 | 2.5% | 4 | 7 | 31 | 152 | - | 0.002668 | -364.140465 | 4.730923235 | 0.093905942 | 0.0858187 |
| Notechis_scutatus_MV_Z77600 | 2.5% | 3 | 35 | 152 | - | - | 0.004551 | -352.841048 | 16.03033982 | 0.000330412 | 0.000301957 |
| Notechis_scutatus_MV_Z77600 | 2.5% | 2 | 39 | - | - | - | 0.017834 | -305.635149 | 63.23623863 | 1.86E-14 | 1.70E-14 |
| Notechis_scutatus_MV_Z77600 | 2.5% | 1 | - | - | - | - | 0.072867 | -255.324519 | 113.5468691 | 2.21E-25 | 2.02E-25 |
| Notechis_scutatus_MV_Z77600 | 2% | 5 | 6 | 32 | 72 | 157 | 0.001784 | -483.619761 | 0 | 1 | 0.999802545 |
| Notechis_scutatus_MV_Z77600 | 2% | 4 | 6 | 32 | 153 | - | 0.003087 | -466.560075 | 17.0596861 | 0.000197486 | 0.000197447 |
| Notechis_scutatus_MV_Z77600 | 2% | 3 | 35 | 153 | - | - | 0.005509 | -446.360411 | 37.25934939 | 8.11E-09 | 8.11E-09 |
| Notechis_scutatus_MV_Z77600 | 2% | 2 | 42 | - | - | - | 0.020968 | -386.292651 | 97.32710983 | 7.34E-22 | 7.34E-22 |
| Notechis_scutatus_MV_Z77600 | 2% | 1 | - | - | - | - | 0.083157 | -323.111213 | 160.5085473 | 1.40E-35 | 1.40E-35 |
| Pseudonaja_textilis_MZRC_10093 | 5% | 4 | 13 | 45 | 187 | - | 0.000443 | -174.763907 | 0 | 1 | 0.541082214 |
| Pseudonaja_textilis_MZRC_10093 | 5% | 3 | 13 | 187 | - | - | 0.001367 | -174.434495 | 0.329411235 | 0.848143346 | 0.458915279 |
| Pseudonaja_textilis_MZRC_10093 | 5% | 2 | 13 | - | - | - | 0.008585 | -149.847459 | 24.91644802 | 3.89E-06 | 2.10E-06 |
| Pseudonaja_textilis_MZRC_10093 | 5% | 5 | 13 | 32 | 83 | 187 | 0.000184 | -146.548155 | 28.21575172 | 7.46E-07 | 4.04E-07 |
| Pseudonaja_textilis_MZRC_10093 | 5% | 1 | - | - | - | - | 0.041274 | -126.206232 | 48.55767468 | 2.86E-11 | 1.55E-11 |
| Pseudonaja_textilis_MZRC_10093 | 4% | 4 | 11 | 45 | 191 | - | 0.000583 | -237.474620 | 0 | 1 | 0.998553176 |
| Pseudonaja_textilis_MZRC_10093 | 4% | 5 | 11 | 34 | 83 | 191 | 0.000365 | -224.326020 | 13.14859945 | 0.001395783 | 0.001393764 |
| Pseudonaja_textilis_MZRC_10093 | 4% | 3 | 11 | 191 | - | - | 0.002335 | -217.789368 | 19.68525133 | 5.31E-05 | 5.31E-05 |
| Pseudonaja_textilis_MZRC_10093 | 4% | 2 | 18 | - | - | - | 0.009582 | -192.554418 | 44.92020163 | 1.76E-10 | 1.76E-10 |
| Pseudonaja_textilis_MZRC_10093 | 4% | 1 | - | - | - | - | 0.040098 | -163.815835 | 73.65878494 | 1.01E-16 | 1.01E-16 |
| Pseudonaja_textilis_MZRC_10093 | 2.5% | 5 | 8 | 38 | 98 | 196 | 0.000718 | -404.877288 | 0 | 1 | 0.998366009 |
| Pseudonaja_textilis_MZRC_10093 | 2.5% | 4 | 8 | 50 | 196 | - | 0.001351 | -392.047100 | 12.8301886 | 0.001636666 | 0.001633991 |
| Pseudonaja_textilis_MZRC_10093 | 2.5% | 3 | 13 | 196 | - | - | 0.004048 | -357.650098 | 47.22718997 | 5.56E-11 | 5.55E-11 |
| Pseudonaja_textilis_MZRC_10093 | 2.5% | 2 | 17 | - | - | - | 0.012223 | -321.124073 | 83.75321552 | 6.50E-19 | 6.49E-19 |
| Pseudonaja_textilis_MZRC_10093 | 2.5% | 1 | - | - | - | - | 0.041268 | -278.635300 | 126.2419879 | 3.86E-28 | 3.86E-28 |
| Pseudonaja_textilis_MZRC_10093 | 2% | 5 | 7 | 49 | 87 | 198 | 0.000985 | -513.900166 | 0 | 1 | 0.897958565 |
| Pseudonaja_textilis_MZRC_10093 | 2% | 4 | 7 | 49 | 198 | - | 0.001329 | -509.550676 | 4.349489929 | 0.113637131 | 0.102041435 |
| Pseudonaja_textilis_MZRC_10093 | 2% | 3 | 18 | 198 | - | - | 0.004615 | -455.392019 | 58.50814701 | 1.97E-13 | 1.77E-13 |
| Pseudonaja_textilis_MZRC_10093 | 2% | 2 | 18 | - | - | - | 0.012296 | -413.513808 | 100.3863571 | 1.59E-22 | 1.43E-22 |
| Pseudonaja_textilis_MZRC_10093 | 2% | 1 | - | - | - | - | 0.043247 | -356.455077 | 157.4450884 | 6.47E-35 | 5.81E-35 |
| Pseudonaja_textilis_MV_D76367 | 5% | 3 | 12 | 80 | - | - | 0.001953 | -166.936788 | 0 | 1 | 0.852600838 |
| Pseudonaja_textilis_MV_D76367 | 5% | 4 | 12 | 31 | 178 | - | 0.000780 | -162.876484 | 4.060303873 | 0.131315568 | 0.111959763 |
| Pseudonaja_textilis_MV_D76367 | 5% | 2 | 12 | - | - | - | 0.005151 | -160.573898 | 6.362889296 | 0.041525622 | 0.03540478 |
| Pseudonaja_textilis_MV_D76367 | 5% | 5 | 12 | 31 | 80 | 169 | 0.000183 | -146.713336 | 20.22345113 | 4.06E-05 | 3.46E-05 |
| Pseudonaja_textilis_MV_D76367 | 5% | 1 | - | - | - | - | 0.038643 | -127.589397 | 39.34739076 | 2.86E-09 | 2.44E-09 |
| Pseudonaja_textilis_MV_D76367 | 4% | 5 | 10 | 49 | 80 | 182 | 0.000325 | -227.386450 | 0 | 1 | 0.98622158 |
| Pseudonaja_textilis_MV_D76367 | 4% | 4 | 10 | 33 | 182 | - | 0.001199 | -218.733533 | 8.652916216 | 0.013214268 | 0.013032196 |
| Pseudonaja_textilis_MV_D76367 | 4% | 3 | 10 | 107 | - | - | 0.002806 | -213.011735 | 14.37471468 | 0.000756085 | 0.000745667 |
| Pseudonaja_textilis_MV_D76367 | 4% | 2 | 10 | - | - | - | 0.007591 | -198.610737 | 28.77571272 | 5.64E-07 | 5.56E-07 |
| Pseudonaja_textilis_MV_D76367 | 4% | 1 | - | - | - | - | 0.041140 | -163.149127 | 64.23732249 | 1.12E-14 | 1.11E-14 |
| Pseudonaja_textilis_MV_D76367 | 2.5% | 5 | 8 | 36 | 88 | 188 | 0.001067 | -388.643881 | 0 | 1 | 0.999142508 |
| Pseudonaja_textilis_MV_D76367 | 2.5% | 4 | 8 | 36 | 88 | - | 0.002071 | -374.522477 | 14.12140376 | 0.000858176 | 0.00085744 |
| Pseudonaja_textilis_MV_D76367 | 2.5% | 3 | 8 | 36 | - | - | 0.004304 | -355.132222 | 33.51165871 | 5.28E-08 | 5.28E-08 |
| Pseudonaja_textilis_MV_D76367 | 2.5% | 2 | 12 | - | - | - | 0.011545 | -323.465554 | 65.17832688 | 7.03E-15 | 7.02E-15 |
| Pseudonaja_textilis_MV_D76367 | 2.5% | 1 | - | - | - | - | 0.042786 | -277.154290 | 111.4895909 | 6.17E-25 | 6.17E-25 |
| Pseudonaja_textilis_MV_D76367 | 2% | 5 | 7 | 41 | 85 | 186 | 0.001264 | -501.199931 | 0 | 1 | 0.99999947 |
| Pseudonaja_textilis_MV_D76367 | 2% | 4 | 7 | 41 | 85 | - | 0.002759 | -472.298656 | 28.90127514 | 5.30E-07 | 5.30E-07 |
| Pseudonaja_textilis_MV_D76367 | 2% | 3 | 10 | 85 | - | - | 0.005541 | -446.071500 | 55.12843096 | 1.07E-12 | 1.07E-12 |
| Pseudonaja_textilis_MV_D76367 | 2% | 2 | 36 | - | - | - | 0.013176 | -409.987547 | 91.21238389 | 1.56E-20 | 1.56E-20 |
| Pseudonaja_textilis_MV_D76367 | 2% | 1 | - | - | - | - | 0.049087 | -349.994953 | 151.204978 | 1.47E-33 | 1.47E-33 |
| Pseudonaja_textilis_MV_D76368 | 5% | 4 | 14 | 44 | 96 | - | 0.000289 | -183.729743 | 0 | 1 | 0.600224548 |
| Pseudonaja_textilis_MV_D76368 | 5% | 3 | 25 | 96 | - | - | 0.000932 | -182.487449 | 1.242294053 | 0.537327755 | 0.322517309 |
| Pseudonaja_textilis_MV_D76368 | 5% | 2 | 34 | - | - | - | 0.002079 | -179.628467 | 4.101275823 | 0.128652808 | 0.077220574 |
| Pseudonaja_textilis_MV_D76368 | 5% | 5 | 14 | 44 | 96 | 185 | 0.000079 | -164.371990 | 19.35775325 | 6.26E-05 | 3.76E-05 |
| Pseudonaja_textilis_MV_D76368 | 5% | 1 | - | - | - | - | 0.018672 | -142.863220 | 40.86652274 | 1.34E-09 | 8.02E-10 |
| Pseudonaja_textilis_MV_D76368 | 4% | 4 | 12 | 44 | 94 | - | 0.000648 | -234.740528 | 0 | 1 | 0.78261055 |
| Pseudonaja_textilis_MV_D76368 | 4% | 3 | 19 | 94 | - | - | 0.001351 | -232.023000 | 2.717527947 | 0.256978213 | 0.20111386 |
| Pseudonaja_textilis_MV_D76368 | 4% | 2 | 36 | - | - | - | 0.002606 | -226.404552 | 8.335976685 | 0.015483376 | 0.012117453 |
| Pseudonaja_textilis_MV_D76368 | 4% | 5 | 12 | 44 | 94 | 167 | 0.000366 | -224.265392 | 10.47513645 | 0.005313162 | 0.004158136 |
| Pseudonaja_textilis_MV_D76368 | 4% | 1 | - | - | - | - | 0.020929 | -180.720727 | 54.01980101 | 1.86E-12 | 1.46E-12 |
| Pseudonaja_textilis_MV_D76368 | 2.5% | 5 | 9 | 44 | 96 | 190 | 0.000448 | -424.287448 | 0 | 1 | 0.999995227 |
| Pseudonaja_textilis_MV_D76368 | 2.5% | 4 | 9 | 44 | 96 | - | 0.001119 | -399.765763 | 24.52168544 | 4.73E-06 | 4.73E-06 |
| Pseudonaja_textilis_MV_D76368 | 2.5% | 3 | 44 | 96 | - | - | 0.001830 | -390.198126 | 34.08932248 | 3.96E-08 | 3.96E-08 |
| Pseudonaja_textilis_MV_D76368 | 2.5% | 2 | 39 | - | - | - | 0.004280 | -364.151643 | 60.1358056 | 8.74E-14 | 8.74E-14 |
| Pseudonaja_textilis_MV_D76368 | 2.5% | 1 | - | - | - | - | 0.025950 | -297.654914 | 126.6325346 | 3.18E-28 | 3.18E-28 |
| Pseudonaja_textilis_MV_D76368 | 2% | 5 | 12 | 48 | 98 | 192 | 0.000914 | -517.725677 | 0 | 1 | 0.999973604 |
| Pseudonaja_textilis_MV_D76368 | 2% | 4 | 12 | 48 | 98 | - | 0.001712 | -496.638963 | 21.08671408 | 2.64E-05 | 2.64E-05 |
| Pseudonaja_textilis_MV_D76368 | 2% | 3 | 48 | 98 | - | - | 0.002685 | -483.013146 | 34.71253081 | 2.90E-08 | 2.90E-08 |
| Pseudonaja_textilis_MV_D76368 | 2% | 2 | 44 | - | - | - | 0.005367 | -455.793352 | 61.93232432 | 3.56E-14 | 3.56E-14 |
| Pseudonaja_textilis_MV_D76368 | 2% | 1 | - | - | - | - | 0.028788 | -377.211614 | 140.5140625 | 3.07E-31 | 3.07E-31 |
| Pseudonaja_textilis_MV_D76369 | 5% | 3 | 13 | 188 | - | - | 0.001305 | -175.407588 | 0 | 1 | 0.95587799 |
| Pseudonaja_textilis_MV_D76369 | 5% | 4 | 13 | 44 | 188 | - | 0.000576 | -169.256245 | 6.15134306 | 0.046158621 | 0.04412201 |
| Pseudonaja_textilis_MV_D76369 | 5% | 5 | 13 | 44 | 105 | 188 | 0.000369 | -131.952261 | 43.45532719 | 3.66E-10 | 3.50E-10 |
| Pseudonaja_textilis_MV_D76369 | 5% | 2 | 13 | - | - | - | 0.031978 | -122.232037 | 53.17555154 | 2.84E-12 | 2.71E-12 |
| Pseudonaja_textilis_MV_D76369 | 5% | 1 | - | - | - | - | 0.075850 | -113.427293 | 61.98029506 | 3.48E-14 | 3.32E-14 |
| Pseudonaja_textilis_MV_D76369 | 4% | 4 | 11 | 36 | 192 | - | 0.000957 | -224.591805 | 0 | 1 | 0.976935463 |
| Pseudonaja_textilis_MV_D76369 | 4% | 3 | 11 | 192 | - | - | 0.002420 | -216.866471 | 7.725334143 | 0.021011885 | 0.020527255 |
| Pseudonaja_textilis_MV_D76369 | 4% | 5 | 11 | 36 | 93 | 192 | 0.000571 | -212.685151 | 11.90665427 | 0.002597185 | 0.002537282 |
| Pseudonaja_textilis_MV_D76369 | 4% | 2 | 11 | - | - | - | 0.029752 | -163.096916 | 61.49488946 | 4.43E-14 | 4.33E-14 |
| Pseudonaja_textilis_MV_D76369 | 4% | 1 | - | - | - | - | 0.074783 | -147.611120 | 76.98068487 | 1.92E-17 | 1.88E-17 |
| Pseudonaja_textilis_MV_D76369 | 2.5% | 5 | 8 | 39 | 105 | 200 | 0.001516 | -374.272478 | 0 | 1 | 0.923750895 |
| Pseudonaja_textilis_MV_D76369 | 2.5% | 4 | 8 | 39 | 193 | - | 0.002353 | -369.283601 | 4.988876871 | 0.082542791 | 0.076248977 |
| Pseudonaja_textilis_MV_D76369 | 2.5% | 3 | 13 | 193 | - | - | 0.005831 | -342.682210 | 31.59026722 | 1.38E-07 | 1.28E-07 |
| Pseudonaja_textilis_MV_D76369 | 2.5% | 2 | 13 | - | - | - | 0.022177 | -296.698110 | 77.5743672 | 1.43E-17 | 1.32E-17 |
| Pseudonaja_textilis_MV_D76369 | 2.5% | 1 | - | - | - | - | 0.064594 | -260.265814 | 114.0066642 | 1.75E-25 | 1.62E-25 |
| Pseudonaja_textilis_MV_D76369 | 2% | 5 | 7 | 48 | 105 | 202 | 0.001897 | -480.510721 | 0 | 1 | 0.999938179 |
| Pseudonaja_textilis_MV_D76369 | 2% | 4 | 7 | 40 | 198 | - | 0.003434 | -461.128324 | 19.38239636 | 6.18E-05 | 6.18E-05 |
| Pseudonaja_textilis_MV_D76369 | 2% | 3 | 15 | 192 | - | - | 0.008376 | -424.999147 | 55.51157325 | 8.83E-13 | 8.83E-13 |
| Pseudonaja_textilis_MV_D76369 | 2% | 2 | 15 | - | - | - | 0.019362 | -390.355682 | 90.15503862 | 2.65E-20 | 2.65E-20 |
| Pseudonaja_textilis_MV_D76369 | 2% | 1 | - | - | - | - | 0.062823 | -337.412470 | 143.0982507 | 8.45E-32 | 8.44E-32 |

**Supplementary Table 10**: Results of each snake’s breakpoint uncertainty, calculated using *MorphoRegions::calcBPvar*. This table showcases each snake’s breakpoints, weighted means (wMean) and weighted standard deviations (wSD).

| Sampling |  | Austrelaps_superbus_MZRC_10088 | | | |
| --- | --- | --- | --- | --- | --- |
|  |  | breakpoint1 | breakpoint2 | breakpoint3 | breakpoint4 |
| 2% | wMean | 7.00 | 27.52 | 73.91 | 150.00 |
|  | wSD | 0.04 | 2.16 | 1.61 | 0.09 |
| 2.50% | wMean | 8.01 | 26.86 | 76.80 | 145.98 |
|  | wSD | 0.15 | 2.16 | 3.59 | 2.35 |
| 4% | wMean | 10.00 | 27.01 | 42.75 | 144.00 |
|  | wSD | 0.00 | 0.32 | 4.11 | 0.049 |
| 5% | wMean | 11.00 | 26.00 | 73.00 | 141.00 |
|  | wSD | 0.00 | 0.07 | 0.15 | 2.62E-05 |
|  |  | Austrelaps_superbus_MV_Z77598 | | | |
| Sampling |  | breakpoint1 | breakpoint2 | breakpoint3 | breakpoint4 |
| 2% | wMean | 6.00 | 35.27 | 75.42 | 142.04 |
|  | wSD | 0.00 | 3.41 | 6.97 | 6.89 |
| 2.50% | wMean | 7.00 | 33.32 | 69.82 | 144.42 |
|  | wSD | 0.00 | 3.01 | 3.96 | 3.63 |
| 4% | wMean | 10.00 | 29.85 | 69.55 | 135.36 |
|  | wSD | 0.00 | 3.97 | 19.93 | 7.95 |
| 5% | wMean | 12.00 | 27.04 | 64.92 | 141.99 |
|  | wSD | 0.00 | 0.68 | 2.00 | 0.35 |
|  |  | Notechis_scutatus_MZRC_10089 | | | |
| Sampling |  | breakpoint1 | breakpoint2 | breakpoint3 | breakpoint4 |
| 2% | wMean | 7.00 | 35.07 | 79.43 | 161.96 |
|  | wSD | 0.00 | 4.34 | 4.79 | 0.37 |
| 2.50% | wMean | 8.00 | 30.05 | 104.01 | 160.95 |
|  | wSD | 0.00 | 0.71 | 25.40 | 1.72 |
| 4% | wMean | 11.00 | 37.39 | 89.48 | 159.00 |
|  | wSD | 0.00 | 6.23 | 13.20 | 0.01 |
| 5% | wMean | 12.00 | 30.03 | 96.89 | 155.00 |
|  | wSD | 0.00 | 0.54 | 1.46 | 0.00 |
|  |  | Notechis_scutatus_MZRC_10091 | | | |
| Sampling |  | breakpoint1 | breakpoint2 | breakpoint3 | breakpoint4 |
| 2% | wMean | 8.73 | 40.36 | 134.56 | 171.00 |
|  | wSD | 2.49 | 4.35 | 33.35 | 0.00 |
| 2.50% | wMean | 8.54 | 24.71 | 44.82 | 168.44 |
|  | wSD | 2.62 | 6.41 | 17.21 | 1.39 |
| 4% | wMean | 11.00 | 36.01 | 108.68 | 164.00 |
|  | wSD | 0.00 | 4.35 | 47.44 | 0.00 |
| 5% | wMean | 13.00 | 33.17 | 64.51 | 161.00 |
|  | wSD | 0.00 | 4.30 | 26.41 | 0.00 |
|  |  | Notechis_scutatus_MV_D76365 | | | |
| Sampling |  | breakpoint1 | breakpoint2 | breakpoint3 | breakpoint4 |
| 2% | wMean | 8.57 | 38.45 | 84.80 | 164.90 |
|  | wSD | 1.05 | 6.21 | 4.46 | 0.66 |
| 2.50% | wMean | 7.00 | 38.31 | 86.17 | 162.99 |
|  | wSD | 0.01 | 3.92 | 5.34 | 0.19 |
| 4% | wMean | 9.00 | 44.14 | 87.09 | 157.98 |
|  | wSD | 0.00 | 16.58 | 12.63 | 0.35 |
| 5% | wMean | 11.00 | 47.64 | 95.03 | 154.00 |
|  | wSD | 0.00 | 22.55 | 19.35 | 0.04 |
|  |  | Notechis_scutatus_MV_D76366 | | | |
| Sampling |  | breakpoint1 | breakpoint2 | breakpoint3 | breakpoint4 |
| 2% | wMean | 10.51 | 34.74 | 90.33 | 158.11 |
|  | wSD | 1.56 | 5.03 | 12.98 | 6.46 |
| 2.50% | wMean | 11.67 | 33.72 | 77.02 | 157.12 |
|  | wSD | 1.37 | 5.76 | 14.80 | 4.40 |
| 4% | wMean | 10.00 | 36.72 | 92.58 | 152.78 |
|  | wSD | 0.00 | 6.74 | 8.97 | 4.50 |
| 5% | wMean | 12.00 | 45.98 | 99.54 | 152.28 |
|  | wSD | 0.00 | 0.70 | 2.90 | 1.65 |
|  |  | Notechis_scutatus_MV_Z77599 | | | |
| Sampling |  | breakpoint1 | breakpoint2 | breakpoint3 | breakpoint4 |
| 2% | wMean | 6.00 | 30.79 | 91.39 | 166.94 |
|  | wSD | 0.00 | 4.10 | 21.26 | 0.60 |
| 2.50% | wMean | 7.00 | 34.13 | 137.99 | 159.87 |
|  | wSD | 0.00 | 5.26 | 0.76 | 2.48 |
| 4% | wMean | 10.00 | 33.18 | 78.23 | 160.00 |
|  | wSD | 0.00 | 2.52 | 3.91 | 0.00 |
| 5% | wMean | 12.00 | 39.25 | 88.10 | 157.00 |
|  | wSD | 0.00 | 11.40 | 9.46 | 0.00 |
|  |  | Notechis_scutatus_MV_Z77600 | | | |
| Sampling |  | breakpoint1 | breakpoint2 | breakpoint3 | breakpoint4 |
| 2% | wMean | 6.00 | 34.60 | 76.05 | 158.84 |
|  | wSD | 0.00 | 7.05 | 3.79 | 3.19 |
| 2.50% | wMean | 7.00 | 34.86 | 81.53 | 167.66 |
|  | wSD | 0.00 | 8.58 | 6.92 | 2.02 |
| 4% | wMean | 11.00 | 39.42 | 74.81 | 156.08 |
|  | wSD | 0.00 | 9.34 | 6.06 | 4.40 |
| 5% | wMean | 13.00 | 36.38 | 84.33 | 155.87 |
|  | wSD | 0.00 | 6.78 | 9.88 | 3.18 |
|  |  | Pseudonaja_textilis_MZRC_10093 | | | |
| Sampling |  | breakpoint1 | breakpoint2 | breakpoint3 | breakpoint4 |
| 2% | wMean | 7.00 | 52.55 | 104.57 | 198.00 |
|  | wSD | 0.00 | 1.63 | 10.13 | 0.00 |
| 2.50% | wMean | 8.00 | 48.36 | 103.30 | 196.00 |
|  | wSD | 0.15 | 7.24 | 15.23 | 0.00 |
| 4% | wMean | 11.00 | 51.99 | 108.12 | 191.00 |
|  | wSD | 0.00 | 3.76 | 16.51 | 0.01 |
| 5% | wMean | 13.00 | 38.45 | 83.54 | 187.00 |
|  | wSD | 0.00 | 6.54 | 4.31 | 0.00 |
|  |  | Pseudonaja_textilis_MV_D76367 | | | |
| Sampling |  | breakpoint1 | breakpoint2 | breakpoint3 | breakpoint4 |
| 2% | wMean | 7.00 | 41.76 | 85.00 | 188.40 |
|  | wSD | 0.03 | 4.53 | 0.05 | 3.69 |
| 2.50% | wMean | 8.00 | 36.96 | 87.96 | 187.89 |
|  | wSD | 0.00 | 2.73 | 0.99 | 0.79 |
| 4% | wMean | 10.00 | 45.62 | 82.74 | 182.00 |
|  | wSD | 0.00 | 5.85 | 9.01 | 0.09 |
| 5% | wMean | 12.00 | 31.76 | 80.37 | 178.00 |
|  | wSD | 0.00 | 3.08 | 3.90 | 0.16 |
|  |  | Pseudonaja_textilis_MV_D76368 | | | |
| Sampling |  | breakpoint1 | breakpoint2 | breakpoint3 | breakpoint4 |
| 2% | wMean | 9.28 | 47.80 | 98.34 | 194.53 |
|  | wSD | 0.87 | 1.25 | 5.11 | 1.96 |
| 2.50% | wMean | 9.00 | 49.92 | 96.02 | 193.62 |
|  | wSD | 0.13 | 2.68 | 0.78 | 1.17 |
| 4% | wMean | 12.00 | 42.04 | 95.06 | 189.00 |
|  | wSD | 0.00 | 3.44 | 12.43 | 0.00 |
| 5% | wMean | 14.00 | 43.98 | 96.55 | 185.00 |
|  | wSD | 0.00 | 0.49 | 3.49 | 0.00 |
|  |  | Pseudonaja_textilis_MV_D76369 | | | |
| Sampling |  | breakpoint1 | breakpoint2 | breakpoint3 | breakpoint4 |
| 2% | wMean | 7.00 | 46.11 | 101.15 | 198.75 |
|  | wSD | 0.04 | 5.38 | 13.89 | 2.46 |
| 2.50% | wMean | 8.00 | 41.10 | 94.95 | 195.15 |
|  | wSD | 0.00 | 4.49 | 12.40 | 3.23 |
| 4% | wMean | 11.00 | 38.06 | 93.05 | 192.00 |
|  | wSD | 0.00 | 5.35 | 22.09 | 0.02 |
| 5% | wMean | 13.00 | 39.58 | 88.47 | 188.00 |
|  | wSD | 0.00 | 5.97 | 22.00 | 0.00 |

**Supplementary Table 11**: The results of the output from *MorphoRegions::modelsupport* tested with 5 regions on 4 different landmarking schemes (All landmarks, Anterior landmarks, Neural Spine landmarks and Hypapophysis landmarks). This table showcases their breakpoints, sum of residual scores, AICc, deltaAICc model likelihood and Akaike weight values.

| **Snake** | **Scheme** | **regions** | **breakpoint1** | **breakpoint2** | **breakpoint3** | **breakpoint4** | **sumRSS** | **AICc** | **deltaAIC** | **model_lik** | **Ak_weight** |
| --- | --- | --- | --- | --- | --- | --- | --- | --- | --- | --- | --- |
| Austrelaps superbus EEM 522503 | All landmarks | 5 | 7 | 30 | 74 | 149 | 0.011 | -4590.897 | 0 | 1 | 1 |
| Austrelaps superbus EEM 522503 | All landmarks | 4 | 8 | 41 | 148 | - | 0.015 | -4480.573 | 110.324 | 1.11E-24 | 1.11E-24 |
| Austrelaps superbus EEM 522503 | All landmarks | 3 | 41 | 148 | - | - | 0.021 | -4352.951 | 237.946 | 2.14E-52 | 2.14E-52 |
| Austrelaps superbus EEM 522503 | All landmarks | 2 | 38 | - | - | - | 0.074 | -3805.773 | 785.124 | 3.25E-171 | 3.25E-171 |
| Austrelaps superbus EEM 522503 | All landmarks | 1 | - | - | - | - | 0.183 | -3421.022 | 1169.875 | 9.22E-255 | 9.22E-255 |
| Austrelaps superbus EEM 522503 | Anterior landmarks | 5 | 6 | 26 | 67 | 147 | 0.019 | -4357.144 | 0 | 1 | 1 |
| Austrelaps superbus EEM 522503 | Anterior landmarks | 4 | 6 | 31 | 144 | - | 0.031 | -4156.507 | 200.637 | 2.71E-44 | 2.71E-44 |
| Austrelaps superbus EEM 522503 | Anterior landmarks | 3 | 26 | 144 | - | - | 0.055 | -3920.767 | 436.377 | 1.75E-95 | 1.75E-95 |
| Austrelaps superbus EEM 522503 | Anterior landmarks | 2 | 26 | - | - | - | 0.087 | -3736.214 | 620.929 | 1.47E-135 | 1.47E-135 |
| Austrelaps superbus EEM 522503 | Anterior landmarks | 1 | - | - | - | - | 0.379 | -3100.512 | 1256.632 | 1.34E-273 | 1.34E-273 |
| Austrelaps superbus EEM 522503 | Neural Spine landmarks | 5 | 7 | 26 | 98 | 151 | 0.102 | -4996.789 | 0 | 1 | 1 |
| Austrelaps superbus EEM 522503 | Neural Spine landmarks | 4 | 7 | 28 | 147 | - | 0.12 | -4919.404 | 77.385 | 1.57E-17 | 1.57E-17 |
| Austrelaps superbus EEM 522503 | Neural Spine landmarks | 3 | 28 | 147 | - | - | 0.149 | -4814.834 | 181.955 | 3.08E-40 | 3.08E-40 |
| Austrelaps superbus EEM 522503 | Neural Spine landmarks | 2 | 28 | - | - | - | 0.232 | -4572.694 | 424.095 | 8.11E-93 | 8.11E-93 |
| Austrelaps superbus EEM 522503 | Neural Spine landmarks | 1 | - | - | - | - | 0.497 | -4143.992 | 852.796 | 6.57E-186 | 6.57E-186 |
| Austrelaps superbus EEM 522503 | Hypapophysis landmarks | 5 | 10 | 45 | 108 | 148 | 0.083 | -3707.565 | 0 | 1 | 1 |
| Austrelaps superbus EEM 522503 | Hypapophysis landmarks | 4 | 10 | 45 | 148 | - | 0.096 | -3663.189 | 44.376 | 2.31E-10 | 2.31E-10 |
| Austrelaps superbus EEM 522503 | Hypapophysis landmarks | 3 | 42 | 148 | - | - | 0.116 | -3594.032 | 113.533 | 2.22E-25 | 2.22E-25 |
| Austrelaps superbus EEM 522503 | Hypapophysis landmarks | 2 | 45 | - | - | - | 0.174 | -3430.752 | 276.813 | 7.78E-61 | 7.78E-61 |
| Austrelaps superbus EEM 522503 | Hypapophysis landmarks | 1 | - | - | - | - | 0.31 | -3190.249 | 517.316 | 4.64E-113 | 4.64E-113 |
| Austrelaps superbus MV Z77598 | All landmarks | 5 | 7 | 32 | 72 | 150 | 0.012 | -4838.427 | 0 | 1 | 1 |
| Austrelaps superbus MV Z77598 | All landmarks | 4 | 7 | 40 | 149 | - | 0.019 | -4639.878 | 198.549 | 7.68E-44 | 7.68E-44 |
| Austrelaps superbus MV Z77598 | All landmarks | 3 | 31 | 150 | - | - | 0.034 | -4386.048 | 452.38 | 5.85E-99 | 5.85E-99 |
| Austrelaps superbus MV Z77598 | All landmarks | 2 | 31 | - | - | - | 0.075 | -4033.17 | 805.258 | 1.38E-175 | 1.38E-175 |
| Austrelaps superbus MV Z77598 | All landmarks | 1 | - | - | - | - | 0.179 | -3643.097 | 1195.33 | 2.74E-260 | 2.74E-260 |
| Austrelaps superbus MV Z77598 | Anterior landmarks | 5 | 4 | 18 | 35 | 143 | 0.021 | -4575.611 | 0 | 1 | 1 |
| Austrelaps superbus MV Z77598 | Anterior landmarks | 4 | 6 | 37 | 143 | - | 0.026 | -4488.342 | 87.268 | 1.12E-19 | 1.12E-19 |
| Austrelaps superbus MV Z77598 | Anterior landmarks | 3 | 6 | 37 | - | - | 0.043 | -4273.965 | 301.646 | 3.15E-66 | 3.15E-66 |
| Austrelaps superbus MV Z77598 | Anterior landmarks | 2 | 31 | - | - | - | 0.065 | -4101.75 | 473.86 | 1.27E-103 | 1.27E-103 |
| Austrelaps superbus MV Z77598 | Anterior landmarks | 1 | - | - | - | - | 0.275 | -3444.326 | 1131.285 | 2.21E-246 | 2.21E-246 |
| Austrelaps superbus MV Z77598 | Neural Spine landmarks | 5 | 4 | 19 | 106 | 150 | 0.08 | -3957.605 | 0 | 1 | 0.999999998 |
| Austrelaps superbus MV Z77598 | Neural Spine landmarks | 4 | 4 | 21 | 121 | - | 0.09 | -3917.175 | 40.43 | 1.66E-09 | 1.66E-09 |
| Austrelaps superbus MV Z77598 | Neural Spine landmarks | 3 | 16 | 117 | - | - | 0.109 | -3846.778 | 110.827 | 8.60E-25 | 8.60E-25 |
| Austrelaps superbus MV Z77598 | Neural Spine landmarks | 2 | 19 | - | - | - | 0.165 | -3665.678 | 291.927 | 4.06E-64 | 4.06E-64 |
| Austrelaps superbus MV Z77598 | Neural Spine landmarks | 1 | - | - | - | - | 0.447 | -3218.354 | 739.251 | 2.98E-161 | 2.98E-161 |
| Austrelaps superbus MV Z77598 | Hypapophysis landmarks | 5 | 7 | 33 | 72 | 151 | 0.057 | -2613.573 | 0 | 1 | 1 |
| Austrelaps superbus MV Z77598 | Hypapophysis landmarks | 4 | 38 | 69 | 151 | - | 0.069 | -2567.581 | 45.992 | 1.03E-10 | 1.03E-10 |
| Austrelaps superbus MV Z77598 | Hypapophysis landmarks | 3 | 33 | 154 | - | - | 0.087 | -2506.792 | 106.781 | 6.50E-24 | 6.50E-24 |
| Austrelaps superbus MV Z77598 | Hypapophysis landmarks | 2 | 42 | - | - | - | 0.161 | -2325.303 | 288.27 | 2.53E-63 | 2.53E-63 |
| Austrelaps superbus MV Z77598 | Hypapophysis landmarks | 1 | - | - | - | - | 0.345 | -2100.02 | 513.553 | 3.04E-112 | 3.04E-112 |
| Notechis scutatus EEM 522501 | All landmarks | 5 | 11 | 32 | 157 | 159 | 0.015 | -5142.966 | 0 | 1 | 1 |
| Notechis scutatus EEM 522501 | All landmarks | 4 | 11 | 32 | 157 | - | 0.019 | -5050 | 92.967 | 6.49E-21 | 6.49E-21 |
| Notechis scutatus EEM 522501 | All landmarks | 3 | 32 | 157 | - | - | 0.035 | -4746.78 | 396.187 | 9.31E-87 | 9.31E-87 |
| Notechis scutatus EEM 522501 | All landmarks | 2 | 36 | - | - | - | 0.138 | -4079.781 | 1063.185 | 1.36E-231 | 1.36E-231 |
| Notechis scutatus EEM 522501 | All landmarks | 1 | - | - | - | - | 0.308 | -3692.492 | 1450.474 | 1.0803e-315 | 1.0803e-315 |
| Notechis scutatus EEM 522501 | Anterior landmarks | 5 | 13 | 36 | 102 | 160 | 0.014 | -3315.579 | 0 | 1 | 1 |
| Notechis scutatus EEM 522501 | Anterior landmarks | 4 | 13 | 36 | 126 | - | 0.018 | -3235.814 | 79.765 | 4.78E-18 | 4.78E-18 |
| Notechis scutatus EEM 522501 | Anterior landmarks | 3 | 25 | 113 | - | - | 0.03 | -3079.106 | 236.473 | 4.47E-52 | 4.47E-52 |
| Notechis scutatus EEM 522501 | Anterior landmarks | 2 | 32 | - | - | - | 0.058 | -2873.291 | 442.288 | 9.09E-97 | 9.09E-97 |
| Notechis scutatus EEM 522501 | Anterior landmarks | 1 | - | - | - | - | 0.39 | -2247.299 | 1068.28 | 1.06E-232 | 1.06E-232 |
| Notechis scutatus EEM 522501 | Neural Spine landmarks | 5 | 7 | 19 | 50 | 157 | 0.086 | -4268.924 | 0 | 1 | 1 |
| Notechis scutatus EEM 522501 | Neural Spine landmarks | 4 | 7 | 36 | 157 | - | 0.1 | -4210.06 | 58.864 | 1.65E-13 | 1.65E-13 |
| Notechis scutatus EEM 522501 | Neural Spine landmarks | 3 | 13 | 157 | - | - | 0.145 | -4040.466 | 228.458 | 2.46E-50 | 2.46E-50 |
| Notechis scutatus EEM 522501 | Neural Spine landmarks | 2 | 17 | - | - | - | 0.341 | -3627.458 | 641.466 | 5.10E-140 | 5.10E-140 |
| Notechis scutatus EEM 522501 | Neural Spine landmarks | 1 | - | - | - | - | 0.74 | -3253.368 | 1015.556 | 2.98E-221 | 2.98E-221 |
| Notechis scutatus EEM 522501 | Hypapophysis landmarks | 5 | 18 | 40 | 152 | 169 | 0.111 | -2623.538 | 0 | 1 | 0.9999983 |
| Notechis scutatus EEM 522501 | Hypapophysis landmarks | 4 | 40 | 152 | 169 | - | 0.124 | -2596.99 | 26.548 | 1.72E-06 | 1.72E-06 |
| Notechis scutatus EEM 522501 | Hypapophysis landmarks | 3 | 40 | 152 | - | - | 0.181 | -2481.88 | 141.659 | 1.73E-31 | 1.73E-31 |
| Notechis scutatus EEM 522501 | Hypapophysis landmarks | 2 | 40 | - | - | - | 0.309 | -2314.88 | 308.658 | 9.46E-68 | 9.46E-68 |
| Notechis scutatus EEM 522501 | Hypapophysis landmarks | 1 | - | - | - | - | 0.522 | -2149.981 | 473.558 | 1.47E-103 | 1.47E-103 |
| Notechis scutatus EEM 522504 | All landmarks | 5 | 11 | 32 | 152 | 172 | 0.012 | -3529.67 | 0 | 1 | 1 |
| Notechis scutatus EEM 522504 | All landmarks | 4 | 11 | 48 | 169 | - | 0.017 | -3409.948 | 119.722 | 1.01E-26 | 1.01E-26 |
| Notechis scutatus EEM 522504 | All landmarks | 3 | 16 | 169 | - | - | 0.03 | -3229.808 | 299.862 | 7.69E-66 | 7.69E-66 |
| Notechis scutatus EEM 522504 | All landmarks | 2 | 19 | - | - | - | 0.103 | -2808.233 | 721.437 | 2.20E-157 | 2.20E-157 |
| Notechis scutatus EEM 522504 | All landmarks | 1 | - | - | - | - | 0.211 | -2569.407 | 960.263 | 3.03E-209 | 3.03E-209 |
| Notechis scutatus EEM 522504 | Anterior landmarks | 5 | 21 | 47 | 109 | 167 | 0.019 | -3363.557 | 0 | 1 | 1 |
| Notechis scutatus EEM 522504 | Anterior landmarks | 4 | 21 | 50 | 157 | - | 0.024 | -3292.862 | 70.695 | 4.45E-16 | 4.45E-16 |
| Notechis scutatus EEM 522504 | Anterior landmarks | 3 | 38 | 156 | - | - | 0.038 | -3146.344 | 217.212 | 6.81E-48 | 6.81E-48 |
| Notechis scutatus EEM 522504 | Anterior landmarks | 2 | 43 | - | - | - | 0.094 | -2840.653 | 522.904 | 2.84E-114 | 2.84E-114 |
| Notechis scutatus EEM 522504 | Anterior landmarks | 1 | - | - | - | - | 0.377 | -2367.87 | 995.687 | 6.16E-217 | 6.16E-217 |
| Notechis scutatus EEM 522504 | Neural Spine landmarks | 5 | 13 | 31 | 160 | 176 | 0.205 | -4021.452 | 0 | 1 | 0.99999976 |
| Notechis scutatus EEM 522504 | Neural Spine landmarks | 4 | 13 | 31 | 174 | - | 0.224 | -3990.963 | 30.489 | 2.40E-07 | 2.40E-07 |
| Notechis scutatus EEM 522504 | Neural Spine landmarks | 3 | 13 | 31 | - | - | 0.269 | -3909.609 | 111.843 | 5.17E-25 | 5.17E-25 |
| Notechis scutatus EEM 522504 | Neural Spine landmarks | 2 | 18 | - | - | - | 0.323 | -3829.167 | 192.285 | 1.76E-42 | 1.76E-42 |
| Notechis scutatus EEM 522504 | Neural Spine landmarks | 1 | - | - | - | - | 0.771 | -3390.189 | 631.263 | 8.37E-138 | 8.37E-138 |
| Notechis scutatus EEM 522504 | Hypapophysis landmarks | 5 | 14 | 32 | 48 | 161 | 0.13 | -4259.711 | 0 | 1 | 0.999999999 |
| Notechis scutatus EEM 522504 | Hypapophysis landmarks | 4 | 14 | 52 | 161 | - | 0.145 | -4217.681 | 42.03 | 7.47E-10 | 7.47E-10 |
| Notechis scutatus EEM 522504 | Hypapophysis landmarks | 3 | 31 | 159 | - | - | 0.188 | -4098.449 | 161.262 | 9.60E-36 | 9.60E-36 |
| Notechis scutatus EEM 522504 | Hypapophysis landmarks | 2 | 48 | - | - | - | 0.247 | -3969.048 | 290.663 | 7.65E-64 | 7.65E-64 |
| Notechis scutatus EEM 522504 | Hypapophysis landmarks | 1 | - | - | - | - | 0.482 | -3635.077 | 624.634 | 2.30E-136 | 2.30E-136 |
| Notechis scutatus MV D76365 | All landmarks | 5 | 10 | 43 | 106 | 159 | 0.009 | -3293.926 | 0 | 1 | 1 |
| Notechis scutatus MV D76365 | All landmarks | 4 | 12 | 89 | 159 | - | 0.014 | -3161.526 | 132.399 | 1.78E-29 | 1.78E-29 |
| Notechis scutatus MV D76365 | All landmarks | 3 | 13 | 151 | - | - | 0.027 | -2973.884 | 320.041 | 3.19E-70 | 3.19E-70 |
| Notechis scutatus MV D76365 | All landmarks | 2 | 17 | - | - | - | 0.069 | -2680.621 | 613.304 | 6.65E-134 | 6.65E-134 |
| Notechis scutatus MV D76365 | All landmarks | 1 | - | - | - | - | 0.184 | -2380.297 | 913.629 | 4.06E-199 | 4.06E-199 |
| Notechis scutatus MV D76365 | Anterior landmarks | 5 | 13 | 33 | 84 | 162 | 0.02 | -4775.477 | 0 | 1 | 1 |
| Notechis scutatus MV D76365 | Anterior landmarks | 4 | 13 | 46 | 162 | - | 0.023 | -4711.908 | 63.569 | 1.57E-14 | 1.57E-14 |
| Notechis scutatus MV D76365 | Anterior landmarks | 3 | 13 | 46 | - | - | 0.031 | -4592.312 | 183.165 | 1.68E-40 | 1.68E-40 |
| Notechis scutatus MV D76365 | Anterior landmarks | 2 | 22 | - | - | - | 0.055 | -4325.186 | 450.291 | 1.66E-98 | 1.66E-98 |
| Notechis scutatus MV D76365 | Anterior landmarks | 1 | - | - | - | - | 0.226 | -3664.924 | 1110.552 | 7.02E-242 | 7.02E-242 |
| Notechis scutatus MV D76365 | Neural Spine landmarks | 5 | 11 | 42 | 126 | 130 | 0.135 | -3852.053 | 0 | 1 | 0.999969078 |
| Notechis scutatus MV D76365 | Neural Spine landmarks | 4 | 11 | 42 | 126 | - | 0.146 | -3831.285 | 20.768 | 3.09E-05 | 3.09E-05 |
| Notechis scutatus MV D76365 | Neural Spine landmarks | 3 | 12 | 126 | - | - | 0.168 | -3777.61 | 74.442 | 6.84E-17 | 6.84E-17 |
| Notechis scutatus MV D76365 | Neural Spine landmarks | 2 | 17 | - | - | - | 0.287 | -3535.35 | 316.703 | 1.69E-69 | 1.69E-69 |
| Notechis scutatus MV D76365 | Neural Spine landmarks | 1 | - | - | - | - | 0.617 | -3183.358 | 668.695 | 6.23E-146 | 6.23E-146 |
| Notechis scutatus MV D76365 | Hypapophysis landmarks | 5 | 16 | 39 | 79 | 167 | 0.065 | -4204.351 | 0 | 1 | 1 |
| Notechis scutatus MV D76365 | Hypapophysis landmarks | 4 | 16 | 39 | 165 | - | 0.076 | -4145.488 | 58.863 | 1.65E-13 | 1.65E-13 |
| Notechis scutatus MV D76365 | Hypapophysis landmarks | 3 | 13 | 98 | - | - | 0.091 | -4072.349 | 132.002 | 2.17E-29 | 2.17E-29 |
| Notechis scutatus MV D76365 | Hypapophysis landmarks | 2 | 30 | - | - | - | 0.122 | -3946.373 | 257.978 | 9.57E-57 | 9.57E-57 |
| Notechis scutatus MV D76365 | Hypapophysis landmarks | 1 | - | - | - | - | 0.261 | -3595.155 | 609.196 | 5.19E-133 | 5.19E-133 |
| Notechis scutatus MV D76366 | All landmarks | 5 | 14 | 32 | 88 | 166 | 0.011 | -3513.433 | 0 | 1 | 1 |
| Notechis scutatus MV D76366 | All landmarks | 4 | 14 | 32 | 162 | - | 0.019 | -3338.187 | 175.246 | 8.83E-39 | 8.83E-39 |
| Notechis scutatus MV D76366 | All landmarks | 3 | 26 | 161 | - | - | 0.027 | -3217.664 | 295.77 | 5.95E-65 | 5.95E-65 |
| Notechis scutatus MV D76366 | All landmarks | 2 | 31 | - | - | - | 0.138 | -2673.096 | 840.337 | 3.34E-183 | 3.34E-183 |
| Notechis scutatus MV D76366 | All landmarks | 1 | - | - | - | - | 0.312 | -2401.345 | 1112.089 | 3.26E-242 | 3.26E-242 |
| Notechis scutatus MV D76366 | Anterior landmarks | 5 | 5 | 15 | 45 | 164 | 0.027 | -5009.399 | 0 | 1 | 1 |
| Notechis scutatus MV D76366 | Anterior landmarks | 4 | 15 | 45 | 164 | - | 0.032 | -4946.817 | 62.582 | 2.57E-14 | 2.57E-14 |
| Notechis scutatus MV D76366 | Anterior landmarks | 3 | 37 | 164 | - | - | 0.048 | -4745.929 | 263.47 | 6.14E-58 | 6.14E-58 |
| Notechis scutatus MV D76366 | Anterior landmarks | 2 | 37 | - | - | - | 0.1 | -4382.779 | 626.62 | 8.54E-137 | 8.54E-137 |
| Notechis scutatus MV D76366 | Anterior landmarks | 1 | - | - | - | - | 0.454 | -3618.62 | 1390.779 | 9.91E-303 | 9.91E-303 |
| Notechis scutatus MV D76366 | Neural Spine landmarks | 5 | 13 | 48 | 157 | 176 | 0.092 | -2779.359 | 0 | 1 | 0.999999323 |
| Notechis scutatus MV D76366 | Neural Spine landmarks | 4 | 13 | 48 | 157 | - | 0.103 | -2750.948 | 28.412 | 6.77E-07 | 6.77E-07 |
| Notechis scutatus MV D76366 | Neural Spine landmarks | 3 | 19 | 157 | - | - | 0.155 | -2621.337 | 158.022 | 4.85E-35 | 4.85E-35 |
| Notechis scutatus MV D76366 | Neural Spine landmarks | 2 | 23 | - | - | - | 0.368 | -2334.649 | 444.71 | 2.71E-97 | 2.71E-97 |
| Notechis scutatus MV D76366 | Neural Spine landmarks | 1 | - | - | - | - | 1.07 | -1977.912 | 801.448 | 9.29E-175 | 9.29E-175 |
| Notechis scutatus MV D76366 | Hypapophysis landmarks | 5 | 15 | 46 | 95 | 176 | 0.057 | -2940.656 | 0 | 1 | 1 |
| Notechis scutatus MV D76366 | Hypapophysis landmarks | 4 | 15 | 46 | 176 | - | 0.07 | -2882.774 | 57.882 | 2.70E-13 | 2.70E-13 |
| Notechis scutatus MV D76366 | Hypapophysis landmarks | 3 | 30 | 176 | - | - | 0.086 | -2825.146 | 115.51 | 8.26E-26 | 8.26E-26 |
| Notechis scutatus MV D76366 | Hypapophysis landmarks | 2 | 26 | - | - | - | 0.164 | -2613.598 | 327.058 | 9.55E-72 | 9.55E-72 |
| Notechis scutatus MV D76366 | Hypapophysis landmarks | 1 | - | - | - | - | 0.237 | -2496.239 | 444.417 | 3.13E-97 | 3.13E-97 |
| Notechis scutatus MV Z77599 | All landmarks | 5 | 7 | 38 | 160 | 169 | 0.02 | -4576.369 | 0 | 1 | 1 |
| Notechis scutatus MV Z77599 | All landmarks | 4 | 7 | 38 | 165 | - | 0.024 | -4491.739 | 84.63 | 4.20E-19 | 4.20E-19 |
| Notechis scutatus MV Z77599 | All landmarks | 3 | 35 | 165 | - | - | 0.039 | -4288.574 | 287.795 | 3.21E-63 | 3.21E-63 |
| Notechis scutatus MV Z77599 | All landmarks | 2 | 160 | - | - | - | 0.129 | -3752.217 | 824.152 | 1.09E-179 | 1.09E-179 |
| Notechis scutatus MV Z77599 | All landmarks | 1 | - | - | - | - | 0.247 | -3467.836 | 1108.534 | 1.93E-241 | 1.93E-241 |
| Notechis scutatus MV Z77599 | Anterior landmarks | 5 | 5 | 33 | 139 | 163 | 0.037 | -4286.072 | 0 | 1 | 1 |
| Notechis scutatus MV Z77599 | Anterior landmarks | 4 | 5 | 33 | 159 | - | 0.044 | -4221.74 | 64.332 | 1.07E-14 | 1.07E-14 |
| Notechis scutatus MV Z77599 | Anterior landmarks | 3 | 29 | 159 | - | - | 0.062 | -4078.198 | 207.874 | 7.26E-46 | 7.26E-46 |
| Notechis scutatus MV Z77599 | Anterior landmarks | 2 | 30 | - | - | - | 0.107 | -3838.322 | 447.75 | 5.92E-98 | 5.92E-98 |
| Notechis scutatus MV Z77599 | Anterior landmarks | 1 | - | - | - | - | 0.348 | -3310.49 | 975.582 | 1.43E-212 | 1.43E-212 |
| Notechis scutatus MV Z77599 | Neural Spine landmarks | 5 | 5 | 29 | 139 | 143 | 0.181 | -3550.906 | 0 | 1 | 1 |
| Notechis scutatus MV Z77599 | Neural Spine landmarks | 4 | 18 | 139 | 143 | - | 0.223 | -3470.048 | 80.859 | 2.77E-18 | 2.77E-18 |
| Notechis scutatus MV Z77599 | Neural Spine landmarks | 3 | 29 | 165 | - | - | 0.281 | -3379.712 | 171.194 | 6.69E-38 | 6.69E-38 |
| Notechis scutatus MV Z77599 | Neural Spine landmarks | 2 | 29 | - | - | - | 0.406 | -3224.692 | 326.214 | 1.46E-71 | 1.46E-71 |
| Notechis scutatus MV Z77599 | Neural Spine landmarks | 1 | - | - | - | - | 0.772 | -2941.904 | 609.003 | 5.71E-133 | 5.71E-133 |
| Notechis scutatus MV Z77599 | Hypapophysis landmarks | 5 | 37 | 72 | 169 | 172 | 0.07 | -2532.515 | 0 | 1 | 0.999811869 |
| Notechis scutatus MV Z77599 | Hypapophysis landmarks | 4 | 37 | 169 | 172 | - | 0.077 | -2515.358 | 17.156 | 0.000188166 | 0.000188131 |
| Notechis scutatus MV Z77599 | Hypapophysis landmarks | 3 | 37 | 171 | - | - | 0.089 | -2479.937 | 52.578 | 3.83E-12 | 3.83E-12 |
| Notechis scutatus MV Z77599 | Hypapophysis landmarks | 2 | 37 | - | - | - | 0.213 | -2222.766 | 309.748 | 5.48E-68 | 5.48E-68 |
| Notechis scutatus MV Z77599 | Hypapophysis landmarks | 1 | - | - | - | - | 0.36 | -2071.166 | 461.349 | 6.60E-101 | 6.60E-101 |
| Notechis scutatus MV Z77600 | All landmarks | 5 | 13 | 33 | 75 | 161 | 0.014 | -3145.846 | 0 | 1 | 1 |
| Notechis scutatus MV Z77600 | All landmarks | 4 | 39 | 73 | 161 | - | 0.018 | -3068.749 | 77.097 | 1.81E-17 | 1.81E-17 |
| Notechis scutatus MV Z77600 | All landmarks | 3 | 39 | 161 | - | - | 0.023 | -3001.706 | 144.14 | 5.02E-32 | 5.02E-32 |
| Notechis scutatus MV Z77600 | All landmarks | 2 | 46 | - | - | - | 0.098 | -2553.639 | 592.207 | 2.53E-129 | 2.53E-129 |
| Notechis scutatus MV Z77600 | All landmarks | 1 | - | - | - | - | 0.295 | -2212.011 | 933.835 | 1.66E-203 | 1.66E-203 |
| Notechis scutatus MV Z77600 | Anterior landmarks | 5 | 13 | 56 | 63 | 159 | 0.034 | -4474.673 | 0 | 1 | 1 |
| Notechis scutatus MV Z77600 | Anterior landmarks | 4 | 13 | 55 | 159 | - | 0.044 | -4373.253 | 101.42 | 9.48E-23 | 9.48E-23 |
| Notechis scutatus MV Z77600 | Anterior landmarks | 3 | 24 | 161 | - | - | 0.065 | -4201.425 | 273.248 | 4.62E-60 | 4.62E-60 |
| Notechis scutatus MV Z77600 | Anterior landmarks | 2 | 24 | - | - | - | 0.098 | -4024.283 | 450.391 | 1.58E-98 | 1.58E-98 |
| Notechis scutatus MV Z77600 | Anterior landmarks | 1 | - | - | - | - | 0.263 | -3567.255 | 907.418 | 9.05E-198 | 9.05E-198 |
| Notechis scutatus MV Z77600 | Neural Spine landmarks | 5 | 8 | 41 | 153 | 158 | 0.106 | -2493.424 | 0 | 1 | 0.999999687 |
| Notechis scutatus MV Z77600 | Neural Spine landmarks | 4 | 8 | 41 | 153 | - | 0.121 | -2463.471 | 29.953 | 3.13E-07 | 3.13E-07 |
| Notechis scutatus MV Z77600 | Neural Spine landmarks | 3 | 8 | 55 | - | - | 0.155 | -2396.168 | 97.256 | 7.61E-22 | 7.61E-22 |
| Notechis scutatus MV Z77600 | Neural Spine landmarks | 2 | 15 | - | - | - | 0.215 | -2301.911 | 191.513 | 2.59E-42 | 2.59E-42 |
| Notechis scutatus MV Z77600 | Neural Spine landmarks | 1 | - | - | - | - | 0.666 | -1953.666 | 539.758 | 6.21E-118 | 6.21E-118 |
| Notechis scutatus MV Z77600 | Hypapophysis landmarks | 5 | 33 | 77 | 154 | 174 | 0.075 | -2605.258 | 0 | 1 | 1 |
| Notechis scutatus MV Z77600 | Hypapophysis landmarks | 4 | 39 | 154 | 174 | - | 0.092 | -2550.151 | 55.107 | 1.08E-12 | 1.08E-12 |
| Notechis scutatus MV Z77600 | Hypapophysis landmarks | 3 | 39 | 171 | - | - | 0.126 | -2461.139 | 144.119 | 5.07E-32 | 5.07E-32 |
| Notechis scutatus MV Z77600 | Hypapophysis landmarks | 2 | 39 | - | - | - | 0.157 | -2402.143 | 203.116 | 7.83E-45 | 7.83E-45 |
| Notechis scutatus MV Z77600 | Hypapophysis landmarks | 1 | - | - | - | - | 0.378 | -2133.189 | 472.069 | 3.10E-103 | 3.10E-103 |
| Pseudonaja textilis EEM 522506 | All landmarks | 5 | 8 | 41 | 101 | 199 | 0.022 | -5774.631 | 0 | 1 | 1 |
| Pseudonaja textilis EEM 522506 | All landmarks | 4 | 8 | 56 | 197 | - | 0.033 | -5573.564 | 201.067 | 2.18E-44 | 2.18E-44 |
| Pseudonaja textilis EEM 522506 | All landmarks | 3 | 32 | 199 | - | - | 0.067 | -5175.166 | 599.465 | 6.73E-131 | 6.73E-131 |
| Pseudonaja textilis EEM 522506 | All landmarks | 2 | 32 | - | - | - | 0.119 | -4858.315 | 916.316 | 1.06E-199 | 1.06E-199 |
| Pseudonaja textilis EEM 522506 | All landmarks | 1 | - | - | - | - | 0.279 | -4384.255 | 1390.376 | 1.21E-302 | 1.21E-302 |
| Pseudonaja textilis EEM 522506 | Anterior landmarks | 5 | 10 | 34 | 40 | 155 | 0.042 | -5413.757 | 0 | 1 | 1 |
| Pseudonaja textilis EEM 522506 | Anterior landmarks | 4 | 10 | 34 | 179 | - | 0.057 | -5249.517 | 164.241 | 2.17E-36 | 2.17E-36 |
| Pseudonaja textilis EEM 522506 | Anterior landmarks | 3 | 10 | 34 | - | - | 0.076 | -5107.475 | 306.282 | 3.10E-67 | 3.10E-67 |
| Pseudonaja textilis EEM 522506 | Anterior landmarks | 2 | 34 | - | - | - | 0.095 | -4991.781 | 421.976 | 2.34E-92 | 2.34E-92 |
| Pseudonaja textilis EEM 522506 | Anterior landmarks | 1 | - | - | - | - | 0.419 | -4149.518 | 1264.239 | 2.98E-275 | 2.98E-275 |
| Pseudonaja textilis EEM 522506 | Neural Spine landmarks | 5 | 7 | 34 | 55 | 194 | 0.162 | -2932.667 | 0 | 1 | 1 |
| Pseudonaja textilis EEM 522506 | Neural Spine landmarks | 4 | 8 | 55 | 194 | - | 0.189 | -2883.996 | 48.671 | 2.70E-11 | 2.70E-11 |
| Pseudonaja textilis EEM 522506 | Neural Spine landmarks | 3 | 9 | 67 | - | - | 0.249 | -2790.527 | 142.14 | 1.36E-31 | 1.36E-31 |
| Pseudonaja textilis EEM 522506 | Neural Spine landmarks | 2 | 21 | - | - | - | 0.426 | -2594.557 | 338.11 | 3.80E-74 | 3.80E-74 |
| Pseudonaja textilis EEM 522506 | Neural Spine landmarks | 1 | - | - | - | - | 1.296 | -2177.374 | 755.293 | 9.78E-165 | 9.78E-165 |
| Pseudonaja textilis EEM 522506 | Hypapophysis landmarks | 5 | 41 | 89 | 176 | 202 | 0.131 | -4757.426 | 0 | 1 | 0.999999997 |
| Pseudonaja textilis EEM 522506 | Hypapophysis landmarks | 4 | 41 | 89 | 189 | - | 0.145 | -4718.387 | 39.039 | 3.33E-09 | 3.33E-09 |
| Pseudonaja textilis EEM 522506 | Hypapophysis landmarks | 3 | 41 | 196 | - | - | 0.164 | -4660.93 | 96.495 | 1.11E-21 | 1.11E-21 |
| Pseudonaja textilis EEM 522506 | Hypapophysis landmarks | 2 | 41 | - | - | - | 0.203 | -4552.697 | 204.728 | 3.50E-45 | 3.50E-45 |
| Pseudonaja textilis EEM 522506 | Hypapophysis landmarks | 1 | - | - | - | - | 0.488 | -4062.367 | 695.058 | 1.18E-151 | 1.18E-151 |
| Pseudonaja textilis MV D76367 | All landmarks | 5 | 9 | 39 | 85 | 194 | 0.018 | -5730.924 | 0 | 1 | 1 |
| Pseudonaja textilis MV D76367 | All landmarks | 4 | 9 | 52 | 189 | - | 0.031 | -5447.748 | 283.176 | 3.23E-62 | 3.23E-62 |
| Pseudonaja textilis MV D76367 | All landmarks | 3 | 21 | 189 | - | - | 0.066 | -5034.64 | 696.284 | 6.37E-152 | 6.37E-152 |
| Pseudonaja textilis MV D76367 | All landmarks | 2 | 39 | - | - | - | 0.129 | -4673.38 | 1057.544 | 2.28E-230 | 2.28E-230 |
| Pseudonaja textilis MV D76367 | All landmarks | 1 | - | - | - | - | 0.261 | -4291.655 | 1439.269 | 2.9291e-313 | 2.9291e-313 |
| Pseudonaja textilis MV D76367 | Anterior landmarks | 5 | 8 | 48 | 73 | 187 | 0.026 | -5530.289 | 0 | 1 | 1 |
| Pseudonaja textilis MV D76367 | Anterior landmarks | 4 | 8 | 52 | 187 | - | 0.03 | -5465.778 | 64.512 | 9.81E-15 | 9.81E-15 |
| Pseudonaja textilis MV D76367 | Anterior landmarks | 3 | 8 | 54 | - | - | 0.056 | -5122.174 | 408.115 | 2.39E-89 | 2.39E-89 |
| Pseudonaja textilis MV D76367 | Anterior landmarks | 2 | 48 | - | - | - | 0.091 | -4871.98 | 658.31 | 1.12E-143 | 1.12E-143 |
| Pseudonaja textilis MV D76367 | Anterior landmarks | 1 | - | - | - | - | 0.369 | -4097.93 | 1432.359 | 9.2705e-312 | 9.2705e-312 |
| Pseudonaja textilis MV D76367 | Neural Spine landmarks | 5 | 7 | 52 | 178 | 200 | 0.135 | -4600.23 | 0 | 1 | 0.999999999 |
| Pseudonaja textilis MV D76367 | Neural Spine landmarks | 4 | 7 | 52 | 178 | - | 0.15 | -4558.592 | 41.638 | 9.09E-10 | 9.09E-10 |
| Pseudonaja textilis MV D76367 | Neural Spine landmarks | 3 | 10 | 178 | - | - | 0.192 | -4436.096 | 164.134 | 2.28E-36 | 2.28E-36 |
| Pseudonaja textilis MV D76367 | Neural Spine landmarks | 2 | 15 | - | - | - | 0.32 | -4163.397 | 436.833 | 1.39E-95 | 1.39E-95 |
| Pseudonaja textilis MV D76367 | Neural Spine landmarks | 1 | - | - | - | - | 0.73 | -3715.537 | 884.693 | 7.79E-193 | 7.79E-193 |
| Pseudonaja textilis MV D76367 | Hypapophysis landmarks | 5 | 42 | 96 | 183 | 200 | 0.1 | -4772.741 | 0 | 1 | 1 |
| Pseudonaja textilis MV D76367 | Hypapophysis landmarks | 4 | 48 | 183 | 200 | - | 0.115 | -4705.645 | 67.096 | 2.69E-15 | 2.69E-15 |
| Pseudonaja textilis MV D76367 | Hypapophysis landmarks | 3 | 51 | 189 | - | - | 0.139 | -4618.27 | 154.47 | 2.87E-34 | 2.87E-34 |
| Pseudonaja textilis MV D76367 | Hypapophysis landmarks | 2 | 51 | - | - | - | 0.278 | -4243.302 | 529.439 | 1.08E-115 | 1.08E-115 |
| Pseudonaja textilis MV D76367 | Hypapophysis landmarks | 1 | - | - | - | - | 0.429 | -4013.047 | 759.694 | 1.08E-165 | 1.08E-165 |
| Pseudonaja textilis MV D76368 | All landmarks | 5 | 9 | 41 | 97 | 196 | 0.016 | -5445.789 | 0 | 1 | 1 |
| Pseudonaja textilis MV D76368 | All landmarks | 4 | 38 | 97 | 196 | - | 0.022 | -5312.748 | 133.041 | 1.29E-29 | 1.29E-29 |
| Pseudonaja textilis MV D76368 | All landmarks | 3 | 39 | 196 | - | - | 0.031 | -5134.977 | 310.812 | 3.22E-68 | 3.22E-68 |
| Pseudonaja textilis MV D76368 | All landmarks | 2 | 41 | - | - | - | 0.09 | -4583.044 | 862.745 | 4.54E-188 | 4.54E-188 |
| Pseudonaja textilis MV D76368 | All landmarks | 1 | - | - | - | - | 0.208 | -4154.323 | 1291.467 | 3.64E-281 | 3.64E-281 |
| Pseudonaja textilis MV D76368 | Anterior landmarks | 5 | 12 | 46 | 100 | 188 | 0.027 | -5176.891 | 0 | 1 | 1 |
| Pseudonaja textilis MV D76368 | Anterior landmarks | 4 | 17 | 80 | 188 | - | 0.032 | -5104.658 | 72.233 | 2.06E-16 | 2.06E-16 |
| Pseudonaja textilis MV D76368 | Anterior landmarks | 3 | 17 | 80 | - | - | 0.043 | -4966.848 | 210.042 | 2.45E-46 | 2.45E-46 |
| Pseudonaja textilis MV D76368 | Anterior landmarks | 2 | 46 | - | - | - | 0.074 | -4689.401 | 487.49 | 1.39E-106 | 1.39E-106 |
| Pseudonaja textilis MV D76368 | Anterior landmarks | 1 | - | - | - | - | 0.249 | -4058.089 | 1118.802 | 1.14E-243 | 1.14E-243 |
| Pseudonaja textilis MV D76368 | Neural Spine landmarks | 5 | 10 | 27 | 143 | 193 | 0.117 | -4396.482 | 0 | 1 | 0.996098238 |
| Pseudonaja textilis MV D76368 | Neural Spine landmarks | 4 | 10 | 27 | 193 | - | 0.124 | -4385.397 | 11.085 | 0.003917045 | 0.003901762 |
| Pseudonaja textilis MV D76368 | Neural Spine landmarks | 3 | 21 | 193 | - | - | 0.155 | -4281.44 | 115.042 | 1.04E-25 | 1.04E-25 |
| Pseudonaja textilis MV D76368 | Neural Spine landmarks | 2 | 31 | - | - | - | 0.194 | -4176.622 | 219.86 | 1.81E-48 | 1.80E-48 |
| Pseudonaja textilis MV D76368 | Neural Spine landmarks | 1 | - | - | - | - | 0.392 | -3817.481 | 579.001 | 1.87E-126 | 1.86E-126 |
| Pseudonaja textilis MV D76368 | Hypapophysis landmarks | 5 | 41 | 95 | 187 | 201 | 0.109 | -4435.202 | 0 | 1 | 1 |
| Pseudonaja textilis MV D76368 | Hypapophysis landmarks | 4 | 41 | 96 | 201 | - | 0.139 | -4322.188 | 113.015 | 2.88E-25 | 2.88E-25 |
| Pseudonaja textilis MV D76368 | Hypapophysis landmarks | 3 | 56 | 201 | - | - | 0.182 | -4195.511 | 239.692 | 8.95E-53 | 8.95E-53 |
| Pseudonaja textilis MV D76368 | Hypapophysis landmarks | 2 | 41 | - | - | - | 0.274 | -3993.355 | 441.848 | 1.13E-96 | 1.13E-96 |
| Pseudonaja textilis MV D76368 | Hypapophysis landmarks | 1 | - | - | - | - | 0.504 | -3683.611 | 751.591 | 6.22E-164 | 6.22E-164 |
| Pseudonaja textilis MV D76369 | All landmarks | 5 | 10 | 52 | 106 | 203 | 0.021 | -6194.703 | 0 | 1 | 1 |
| Pseudonaja textilis MV D76369 | All landmarks | 4 | 10 | 52 | 203 | - | 0.029 | -6000.341 | 194.362 | 6.24E-43 | 6.24E-43 |
| Pseudonaja textilis MV D76369 | All landmarks | 3 | 48 | 203 | - | - | 0.055 | -5632.493 | 562.21 | 8.27E-123 | 8.27E-123 |
| Pseudonaja textilis MV D76369 | All landmarks | 2 | 48 | - | - | - | 0.215 | -4814.293 | 1380.41 | 1.77E-300 | 1.77E-300 |
| Pseudonaja textilis MV D76369 | All landmarks | 1 | - | - | - | - | 0.389 | -4468.37 | 1726.333 | 0 | 0 |
| Pseudonaja textilis MV D76369 | Anterior landmarks | 5 | 8 | 42 | 105 | 198 | 0.033 | -5905.039 | 0 | 1 | 1 |
| Pseudonaja textilis MV D76369 | Anterior landmarks | 4 | 9 | 71 | 195 | - | 0.048 | -5697.256 | 207.783 | 7.59E-46 | 7.59E-46 |
| Pseudonaja textilis MV D76369 | Anterior landmarks | 3 | 53 | 195 | - | - | 0.092 | -5316.769 | 588.27 | 1.81E-128 | 1.81E-128 |
| Pseudonaja textilis MV D76369 | Anterior landmarks | 2 | 62 | - | - | - | 0.162 | -4986.55 | 918.489 | 3.57E-200 | 3.57E-200 |
| Pseudonaja textilis MV D76369 | Anterior landmarks | 1 | - | - | - | - | 0.574 | -4230.211 | 1674.828 | 0 | 0 |
| Pseudonaja textilis MV D76369 | Neural Spine landmarks | 5 | 7 | 29 | 183 | 208 | 0.187 | -4853.783 | 0 | 1 | 1 |
| Pseudonaja textilis MV D76369 | Neural Spine landmarks | 4 | 7 | 29 | 204 | - | 0.211 | -4795.068 | 58.715 | 1.78E-13 | 1.78E-13 |
| Pseudonaja textilis MV D76369 | Neural Spine landmarks | 3 | 14 | 195 | - | - | 0.263 | -4676.856 | 176.927 | 3.81E-39 | 3.81E-39 |
| Pseudonaja textilis MV D76369 | Neural Spine landmarks | 2 | 16 | - | - | - | 0.367 | -4489.228 | 364.555 | 6.88E-80 | 6.88E-80 |
| Pseudonaja textilis MV D76369 | Neural Spine landmarks | 1 | - | - | - | - | 1.082 | -3844.693 | 1009.09 | 7.57E-220 | 7.57E-220 |
| Pseudonaja textilis MV D76369 | Hypapophysis landmarks | 5 | 10 | 60 | 135 | 204 | 0.108 | -5185.89 | 0 | 1 | 1 |
| Pseudonaja textilis MV D76369 | Hypapophysis landmarks | 4 | 43 | 164 | 205 | - | 0.128 | -5100.523 | 85.367 | 2.90E-19 | 2.90E-19 |
| Pseudonaja textilis MV D76369 | Hypapophysis landmarks | 3 | 43 | 203 | - | - | 0.153 | -5006.316 | 179.574 | 1.01E-39 | 1.01E-39 |
| Pseudonaja textilis MV D76369 | Hypapophysis landmarks | 2 | 43 | - | - | - | 0.315 | -4581.495 | 604.395 | 5.72E-132 | 5.72E-132 |
| Pseudonaja textilis MV D76369 | Hypapophysis landmarks | 1 | - | - | - | - | 0.511 | -4301.721 | 884.169 | 1.01E-192 | 1.01E-192 |

**Supplementary Table 12:** Table of PC Scores for the PCA of each region.

| **Region PCA** |  | **Importance of Components:** | | | | | | | | | |
| --- | --- | --- | --- | --- | --- | --- | --- | --- | --- | --- | --- |
|  |  | **Comp1** | **Comp2** | **Comp3** | **Comp4** | **Comp5** | **Comp6** | **Comp7** | **Comp8** | **Comp9** | **Comp10** |
| Cervical | Eigenvalues | 0.003205 | 0.001327 | 0.000552 | 0.000449 | 0.000318 | 0.000204 | 0.000161 | 0.000121 | 0.000107 | 0.000089 |
| Cervical | Proportion of Variance | 0.441656 | 0.182862 | 0.076044 | 0.061870 | 0.043874 | 0.028074 | 0.022206 | 0.016637 | 0.014712 | 0.012290 |
| Cervical | Cumulative Proportion | 0.441656 | 0.624518 | 0.700562 | 0.762432 | 0.806306 | 0.834380 | 0.856586 | 0.873223 | 0.887935 | 0.900225 |
| Anterior Thoracic | Eigenvalues | 0.002225 | 0.001205 | 0.000497 | 0.000314 | 0.000249 | 0.000189 | 0.000116 | 0.000071 | 0.000065 | 0.000048 |
| Anterior Thoracic | Proportion of Variance | 0.406580 | 0.220230 | 0.090882 | 0.057361 | 0.045530 | 0.034554 | 0.021121 | 0.012928 | 0.011930 | 0.008754 |
| Anterior Thoracic | Cumulative Proportion | 0.406580 | 0.626810 | 0.717692 | 0.775053 | 0.820583 | 0.855137 | 0.876258 | 0.889187 | 0.901116 | 0.909871 |
| Middle Thoracic | Eigenvalues | 0.001566 | 0.000795 | 0.000484 | 0.000277 | 0.000175 | 0.000149 | 0.000114 | 0.000059 | 0.000057 | 0.000045 |
| Middle Thoracic | Proportion of Variance | 0.377777 | 0.191695 | 0.116766 | 0.066876 | 0.042286 | 0.035953 | 0.027595 | 0.014339 | 0.013635 | 0.010861 |
| Middle Thoracic | Cumulative Proportion | 0.377777 | 0.569472 | 0.686238 | 0.753114 | 0.795400 | 0.831354 | 0.858948 | 0.873287 | 0.886922 | 0.897783 |
| Posterior Thoracic | Eigenvalues | 0.002188 | 0.000712 | 0.000313 | 0.000284 | 0.000226 | 0.000155 | 0.000127 | 0.000090 | 0.000075 | 0.000058 |
| Posterior Thoracic | Proportion of Variance | 0.458928 | 0.149283 | 0.065591 | 0.059652 | 0.047373 | 0.032432 | 0.026691 | 0.018880 | 0.015690 | 0.012067 |
| Posterior Thoracic | Cumulative Proportion | 0.458928 | 0.608210 | 0.673802 | 0.733453 | 0.780827 | 0.813258 | 0.839950 | 0.858830 | 0.874519 | 0.886587 |
| Lumbar | Eigenvalues | 0.005811 | 0.001088 | 0.000934 | 0.000512 | 0.000394 | 0.000329 | 0.000270 | 0.000200 | 0.000166 | 0.000153 |
| Lumbar | Proportion of Variance | 0.532374 | 0.099689 | 0.085569 | 0.046921 | 0.036138 | 0.030161 | 0.024766 | 0.018315 | 0.015180 | 0.014039 |
| Lumbar | Cumulative Proportion | 0.532374 | 0.632063 | 0.717632 | 0.764553 | 0.800691 | 0.830852 | 0.855618 | 0.873932 | 0.889112 | 0.903152 |

**Supplementary Table 13**: The results of the output from *MorphoRegions::modelsupport* tested with a maximum of 8 regions, tested on every other vertebrae along the precaudal column. This table showcases their breakpoints, sum of residual scores, AICc, deltaAICc model likelihood and Akaike weight values.

| **Snake** | **regions** | **breakpoint1** | **breakpoint2** | **breakpoint3** | **breakpoint4** | **breakpoint5** | **breakpoint6** | **breakpoint7** | **sumRSS** | **AICc** | **deltaAIC** | **model_lik** | **Ak_weight** |
| --- | --- | --- | --- | --- | --- | --- | --- | --- | --- | --- | --- | --- | --- |
| Austrelaps_superbus_MZRC_10088 | 8 | 6 | 12 | 37 | 60 | 82 | 145 | 151 | 0.04262 | -1819.19 | 0.00 | 1 | 0.995939479 |
| Austrelaps_superbus_MZRC_10088 | 7 | 8 | 37 | 60 | 82 | 145 | 151 | - | 0.04706 | -1808.18 | 11.00 | 0.004076989 | 0.004060435 |
| Austrelaps_superbus_MZRC_10088 | 6 | 8 | 37 | 60 | 82 | 149 | - | - | 0.05437 | -1786.66 | 32.52 | 8.66E-08 | 8.62E-08 |
| Austrelaps_superbus_MZRC_10088 | 5 | 8 | 37 | 58 | 149 | - | - | - | 0.06180 | -1768.33 | 50.85 | 9.07E-12 | 9.03E-12 |
| Austrelaps_superbus_MZRC_10088 | 4 | 8 | 45 | 149 | - | - | - | - | 0.08562 | -1705.66 | 113.53 | 2.23E-25 | 2.22E-25 |
| Austrelaps_superbus_MZRC_10088 | 3 | 39 | 149 | - | - | - | - | - | 0.15282 | -1586.40 | 232.79 | 2.82E-51 | 2.81E-51 |
| Austrelaps_superbus_MZRC_10088 | 2 | 39 | - | - | - | - | - | - | 0.25463 | -1482.03 | 337.15 | 6.13E-74 | 6.11E-74 |
| Austrelaps_superbus_MZRC_10088 | 1 | - | - | - | - | - | - | - | 0.57931 | -1308.20 | 510.99 | 1.10E-111 | 1.09E-111 |
| Austrelaps_superbus_MV_Z77598 | 8 | 7 | 30 | 34 | 66 | 86 | 148 | 152 | 0.04465 | -1895.21 | 0.00 | 1 | 0.992748387 |
| Austrelaps_superbus_MV_Z77598 | 7 | 7 | 36 | 66 | 88 | 148 | 152 | - | 0.04884 | -1885.35 | 9.86 | 0.007221227 | 0.007168861 |
| Austrelaps_superbus_MV_Z77598 | 6 | 7 | 36 | 66 | 86 | 148 | - | - | 0.05310 | -1876.43 | 18.78 | 8.34E-05 | 8.28E-05 |
| Austrelaps_superbus_MV_Z77598 | 5 | 7 | 36 | 66 | 148 | - | - | - | 0.07275 | -1813.72 | 81.49 | 2.01E-18 | 2.00E-18 |
| Austrelaps_superbus_MV_Z77598 | 4 | 7 | 40 | 150 | - | - | - | - | 0.08679 | -1782.60 | 112.61 | 3.52E-25 | 3.50E-25 |
| Austrelaps_superbus_MV_Z77598 | 3 | 7 | 38 | - | - | - | - | - | 0.17651 | -1627.90 | 267.31 | 8.98E-59 | 8.92E-59 |
| Austrelaps_superbus_MV_Z77598 | 2 | 34 | - | - | - | - | - | - | 0.27313 | -1535.99 | 359.22 | 9.89E-79 | 9.82E-79 |
| Austrelaps_superbus_MV_Z77598 | 1 | - | - | - | - | - | - | - | 0.60277 | -1361.76 | 533.45 | 1.45E-116 | 1.44E-116 |
| Notechis_scutatus_MZRC_10089 | 8 | 6 | 14 | 39 | 53 | 100 | 158 | 168 | 0.07506 | -2725.67 | 0.00 | 1 | 0.999999719 |
| Notechis_scutatus_MZRC_10089 | 7 | 6 | 12 | 39 | 154 | 158 | 168 | - | 0.08534 | -2695.49 | 30.17 | 2.81E-07 | 2.81E-07 |
| Notechis_scutatus_MZRC_10089 | 6 | 6 | 12 | 39 | 158 | 166 | - | - | 0.09340 | -2677.75 | 47.92 | 3.93E-11 | 3.93E-11 |
| Notechis_scutatus_MZRC_10089 | 5 | 8 | 37 | 158 | 166 | - | - | - | 0.10302 | -2656.94 | 68.73 | 1.19E-15 | 1.19E-15 |
| Notechis_scutatus_MZRC_10089 | 4 | 8 | 37 | 160 | - | - | - | - | 0.13206 | -2585.25 | 140.41 | 3.23E-31 | 3.23E-31 |
| Notechis_scutatus_MZRC_10089 | 3 | 33 | 160 | - | - | - | - | - | 0.20543 | -2448.16 | 277.51 | 5.50E-61 | 5.50E-61 |
| Notechis_scutatus_MZRC_10089 | 2 | 158 | - | - | - | - | - | - | 0.46473 | -2184.89 | 540.77 | 3.74E-118 | 3.74E-118 |
| Notechis_scutatus_MZRC_10089 | 1 | - | - | - | - | - | - | - | 0.81955 | -2004.97 | 720.69 | 3.19E-157 | 3.19E-157 |
| Notechis_scutatus_MZRC_10091 | 8 | 10 | 30 | 42 | 52 | 141 | 159 | 173 | 0.07554 | -2837.06 | 0.00 | 1 | 0.98685042 |
| Notechis_scutatus_MZRC_10091 | 7 | 10 | 42 | 62 | 141 | 159 | 173 | - | 0.08035 | -2828.42 | 8.64 | 0.013296312 | 0.013121472 |
| Notechis_scutatus_MZRC_10091 | 6 | 10 | 42 | 62 | 145 | 173 | - | - | 0.08627 | -2816.13 | 20.93 | 2.85E-05 | 2.81E-05 |
| Notechis_scutatus_MZRC_10091 | 5 | 10 | 40 | 145 | 173 | - | - | - | 0.09782 | -2784.46 | 52.60 | 3.79E-12 | 3.74E-12 |
| Notechis_scutatus_MZRC_10091 | 4 | 10 | 42 | 169 | - | - | - | - | 0.12931 | -2699.00 | 138.05 | 1.05E-30 | 1.04E-30 |
| Notechis_scutatus_MZRC_10091 | 3 | 20 | 169 | - | - | - | - | - | 0.21433 | -2534.49 | 302.57 | 1.98E-66 | 1.96E-66 |
| Notechis_scutatus_MZRC_10091 | 2 | 20 | - | - | - | - | - | - | 0.37798 | -2348.05 | 489.01 | 6.51E-107 | 6.43E-107 |
| Notechis_scutatus_MZRC_10091 | 1 | - | - | - | - | - | - | - | 0.63943 | -2175.76 | 661.30 | 2.52E-144 | 2.48E-144 |
| Notechis_scutatus_MV_D76366 | 8 | 7 | 17 | 35 | 67 | 95 | 155 | 172 | 0.02723 | -2283.69 | 0.00 | 1 | 0.999999995 |
| Notechis_scutatus_MV_D76366 | 7 | 13 | 35 | 67 | 95 | 155 | 172 | - | 0.03289 | -2245.45 | 38.24 | 4.96E-09 | 4.96E-09 |
| Notechis_scutatus_MV_D76366 | 6 | 13 | 35 | 100 | 155 | 172 | - | - | 0.03920 | -2210.25 | 73.44 | 1.13E-16 | 1.13E-16 |
| Notechis_scutatus_MV_D76366 | 5 | 13 | 39 | 143 | 170 | - | - | - | 0.04582 | -2179.76 | 103.93 | 2.71E-23 | 2.71E-23 |
| Notechis_scutatus_MV_D76366 | 4 | 17 | 100 | 168 | - | - | - | - | 0.06144 | -2113.56 | 170.13 | 1.14E-37 | 1.14E-37 |
| Notechis_scutatus_MV_D76366 | 3 | 21 | 164 | - | - | - | - | - | 0.08813 | -2029.60 | 254.09 | 6.68E-56 | 6.68E-56 |
| Notechis_scutatus_MV_D76366 | 2 | 25 | - | - | - | - | - | - | 0.36024 | -1675.18 | 608.51 | 7.32E-133 | 7.32E-133 |
| Notechis_scutatus_MV_D76366 | 1 | - | - | - | - | - | - | - | 0.71224 | -1507.88 | 775.81 | 3.42E-169 | 3.42E-169 |
| Notechis_scutatus_MV_Z77599 | 8 | 5 | 12 | 34 | 60 | 88 | 163 | 169 | 0.06830 | -2491.17 | 0.00 | 1 | 0.999108214 |
| Notechis_scutatus_MV_Z77599 | 7 | 7 | 32 | 60 | 88 | 163 | 169 | - | 0.07464 | -2477.12 | 14.04 | 0.000892571 | 0.000891775 |
| Notechis_scutatus_MV_Z77599 | 6 | 7 | 27 | 67 | 163 | 169 | - | - | 0.08374 | -2454.52 | 36.65 | 1.10E-08 | 1.10E-08 |
| Notechis_scutatus_MV_Z77599 | 5 | 7 | 38 | 163 | 169 | - | - | - | 0.09432 | -2430.26 | 60.91 | 5.95E-14 | 5.94E-14 |
| Notechis_scutatus_MV_Z77599 | 4 | 7 | 40 | 165 | - | - | - | - | 0.10646 | -2404.91 | 86.26 | 1.86E-19 | 1.85E-19 |
| Notechis_scutatus_MV_Z77599 | 3 | 27 | 165 | - | - | - | - | - | 0.19376 | -2231.97 | 259.19 | 5.21E-57 | 5.20E-57 |
| Notechis_scutatus_MV_Z77599 | 2 | 165 | - | - | - | - | - | - | 0.40294 | -2017.60 | 473.57 | 1.46E-103 | 1.46E-103 |
| Notechis_scutatus_MV_Z77599 | 1 | - | - | - | - | - | - | - | 0.68888 | -1863.18 | 627.99 | 4.30E-137 | 4.29E-137 |
| Notechis_scutatus_MV_Z77600 | 8 | 7 | 13 | 41 | 68 | 92 | 102 | 161 | 0.03515 | -2009.67 | 0.00 | 1 | 0.999981726 |
| Notechis_scutatus_MV_Z77600 | 7 | 7 | 13 | 41 | 68 | 86 | 161 | - | 0.04033 | -1987.85 | 21.82 | 1.83E-05 | 1.83E-05 |
| Notechis_scutatus_MV_Z77600 | 6 | 7 | 13 | 43 | 156 | 165 | - | - | 0.04583 | -1967.88 | 41.79 | 8.44E-10 | 8.44E-10 |
| Notechis_scutatus_MV_Z77600 | 5 | 7 | 13 | 43 | 161 | - | - | - | 0.04944 | -1959.88 | 49.79 | 1.55E-11 | 1.55E-11 |
| Notechis_scutatus_MV_Z77600 | 4 | 11 | 43 | 161 | - | - | - | - | 0.05907 | -1927.28 | 82.38 | 1.29E-18 | 1.29E-18 |
| Notechis_scutatus_MV_Z77600 | 3 | 13 | 161 | - | - | - | - | - | 0.16552 | -1692.32 | 317.35 | 1.23E-69 | 1.23E-69 |
| Notechis_scutatus_MV_Z77600 | 2 | 43 | - | - | - | - | - | - | 0.43221 | -1473.76 | 535.90 | 4.27E-117 | 4.27E-117 |
| Notechis_scutatus_MV_Z77600 | 1 | - | - | - | - | - | - | - | 0.84355 | -1323.89 | 685.78 | 1.22E-149 | 1.22E-149 |
| Pseudonaja_textilis_MZRC_10093 | 8 | 9 | 31 | 45 | 97 | 103 | 189 | 201 | 0.04910 | -2421.53 | 0.00 | 1 | 0.999991492 |
| Pseudonaja_textilis_MZRC_10093 | 7 | 9 | 45 | 95 | 105 | 189 | 201 | - | 0.05516 | -2398.18 | 23.35 | 8.51E-06 | 8.51E-06 |
| Pseudonaja_textilis_MZRC_10093 | 6 | 9 | 45 | 81 | 187 | 201 | - | - | 0.06361 | -2367.00 | 54.53 | 1.44E-12 | 1.44E-12 |
| Pseudonaja_textilis_MZRC_10093 | 5 | 9 | 45 | 77 | 199 | - | - | - | 0.07689 | -2321.93 | 99.60 | 2.35E-22 | 2.35E-22 |
| Pseudonaja_textilis_MZRC_10093 | 4 | 9 | 55 | 199 | - | - | - | - | 0.09583 | -2267.81 | 153.72 | 4.16E-34 | 4.16E-34 |
| Pseudonaja_textilis_MZRC_10093 | 3 | 33 | 199 | - | - | - | - | - | 0.17298 | -2106.72 | 314.81 | 4.36E-69 | 4.36E-69 |
| Pseudonaja_textilis_MZRC_10093 | 2 | 33 | - | - | - | - | - | - | 0.29650 | -1960.26 | 461.27 | 6.88E-101 | 6.88E-101 |
| Pseudonaja_textilis_MZRC_10093 | 1 | - | - | - | - | - | - | - | 0.66716 | -1735.19 | 686.34 | 9.21E-150 | 9.21E-150 |
| Pseudonaja_textilis_MV_D76365 | 8 | 9 | 18 | 51 | 106 | 110 | 157 | 167 | 0.06017 | -2645.56 | 0.00 | 1 | 0.99460076 |
| Pseudonaja_textilis_MV_D76365 | 7 | 9 | 18 | 49 | 100 | 157 | 167 | - | 0.06477 | -2635.12 | 10.43 | 0.005428549 | 0.005399239 |
| Pseudonaja_textilis_MV_D76365 | 6 | 11 | 47 | 100 | 157 | 167 | - | - | 0.07413 | -2604.61 | 40.94 | 1.29E-09 | 1.28E-09 |
| Pseudonaja_textilis_MV_D76365 | 5 | 11 | 45 | 154 | 167 | - | - | - | 0.08163 | -2586.07 | 59.49 | 1.21E-13 | 1.20E-13 |
| Pseudonaja_textilis_MV_D76365 | 4 | 11 | 45 | 159 | - | - | - | - | 0.09517 | -2548.81 | 96.74 | 9.83E-22 | 9.78E-22 |
| Pseudonaja_textilis_MV_D76365 | 3 | 13 | 159 | - | - | - | - | - | 0.20589 | -2313.32 | 332.24 | 7.16E-73 | 7.12E-73 |
| Pseudonaja_textilis_MV_D76365 | 2 | 16 | - | - | - | - | - | - | 0.36505 | -2141.14 | 504.41 | 2.94E-110 | 2.92E-110 |
| Pseudonaja_textilis_MV_D76365 | 1 | - | - | - | - | - | - | - | 0.65314 | -1965.71 | 679.85 | 2.36E-148 | 2.35E-148 |
| Pseudonaja_textilis_MV_D76367 | 8 | 7 | 37 | 44 | 83 | 89 | 177 | 197 | 0.06318 | -3170.56 | 0.00 | 1 | 1 |
| Pseudonaja_textilis_MV_D76367 | 7 | 7 | 44 | 83 | 89 | 177 | 197 | - | 0.07331 | -3127.22 | 43.34 | 3.88E-10 | 3.88E-10 |
| Pseudonaja_textilis_MV_D76367 | 6 | 7 | 17 | 53 | 173 | 197 | - | - | 0.07860 | -3113.27 | 57.29 | 3.62E-13 | 3.62E-13 |
| Pseudonaja_textilis_MV_D76367 | 5 | 9 | 48 | 173 | 197 | - | - | - | 0.08911 | -3077.99 | 92.57 | 7.91E-21 | 7.91E-21 |
| Pseudonaja_textilis_MV_D76367 | 4 | 9 | 48 | 195 | - | - | - | - | 0.10387 | -3031.90 | 138.67 | 7.74E-31 | 7.74E-31 |
| Pseudonaja_textilis_MV_D76367 | 3 | 35 | 195 | - | - | - | - | - | 0.21379 | -2771.69 | 398.88 | 2.43E-87 | 2.43E-87 |
| Pseudonaja_textilis_MV_D76367 | 2 | 35 | - | - | - | - | - | - | 0.37821 | -2568.11 | 602.45 | 1.51E-131 | 1.51E-131 |
| Pseudonaja_textilis_MV_D76367 | 1 | - | - | - | - | - | - | - | 0.65894 | -2369.97 | 800.59 | 1.43E-174 | 1.43E-174 |
| Pseudonaja_textilis_MV_D76368 | 8 | 9 | 36 | 43 | 80 | 104 | 191 | 199 | 0.03747 | -2290.45 | 0.00 | 1 | 0.999992885 |
| Pseudonaja_textilis_MV_D76368 | 7 | 9 | 34 | 89 | 102 | 191 | 199 | - | 0.04257 | -2266.74 | 23.71 | 7.11E-06 | 7.11E-06 |
| Pseudonaja_textilis_MV_D76368 | 6 | 9 | 34 | 89 | 104 | 197 | - | - | 0.04951 | -2236.45 | 54.00 | 1.88E-12 | 1.88E-12 |
| Pseudonaja_textilis_MV_D76368 | 5 | 27 | 89 | 104 | 197 | - | - | - | 0.06288 | -2182.29 | 108.16 | 3.27E-24 | 3.27E-24 |
| Pseudonaja_textilis_MV_D76368 | 4 | 34 | 104 | 195 | - | - | - | - | 0.07853 | -2132.35 | 158.10 | 4.67E-35 | 4.67E-35 |
| Pseudonaja_textilis_MV_D76368 | 3 | 38 | 195 | - | - | - | - | - | 0.09651 | -2086.39 | 204.06 | 4.89E-45 | 4.89E-45 |
| Pseudonaja_textilis_MV_D76368 | 2 | 36 | - | - | - | - | - | - | 0.31309 | -1780.99 | 509.46 | 2.36E-111 | 2.36E-111 |
| Pseudonaja_textilis_MV_D76368 | 1 | - | - | - | - | - | - | - | 0.69617 | -1576.17 | 714.28 | 7.87E-156 | 7.87E-156 |
| Pseudonaja_textilis_MV_D76369 | 8 | 7 | 24 | 50 | 84 | 118 | 194 | 204 | 0.04774 | -3597.29 | 0.00 | 1 | 0.999999922 |
| Pseudonaja_textilis_MV_D76369 | 7 | 9 | 48 | 82 | 118 | 194 | 204 | - | 0.05333 | -3564.55 | 32.75 | 7.75E-08 | 7.75E-08 |
| Pseudonaja_textilis_MV_D76369 | 6 | 9 | 52 | 126 | 194 | 204 | - | - | 0.06255 | -3511.52 | 85.77 | 2.37E-19 | 2.37E-19 |
| Pseudonaja_textilis_MV_D76369 | 5 | 9 | 52 | 126 | 202 | - | - | - | 0.07345 | -3457.68 | 139.61 | 4.82E-31 | 4.82E-31 |
| Pseudonaja_textilis_MV_D76369 | 4 | 9 | 60 | 202 | - | - | - | - | 0.09691 | -3355.98 | 241.32 | 3.97E-53 | 3.97E-53 |
| Pseudonaja_textilis_MV_D76369 | 3 | 48 | 202 | - | - | - | - | - | 0.24243 | -2993.00 | 604.29 | 6.02E-132 | 6.02E-132 |
| Pseudonaja_textilis_MV_D76369 | 2 | 202 | - | - | - | - | - | - | 0.55477 | -2666.07 | 931.22 | 6.13E-203 | 6.13E-203 |
| Pseudonaja_textilis_MV_D76369 | 1 | - | - | - | - | - | - | - | 0.93872 | -2462.04 | 1135.26 | 3.04E-247 | 3.04E-247 |

**Supplementary Table 14:** Table showing a Procrustes analysis of variance (ANOVA) test on landmarking 50 vertebrae from the same snake twice.

| Procrustes ANOVA | | | | | | | |
| --- | --- | --- | --- | --- | --- | --- | --- |
| Permutation procedure | | | Randomization of null model residuals | | | | |
| Number of permutations | | | 5000 | | | | |
| Estimation method | | | Ordinary Least Squares | | | | |
| Sums of Squares (SS) and Cross-products: | | | Type 1 | | | | |
| Effect sizes (Z) based on F distributions | | | | | | | |
|  | Df | SS | MS | R^2^ | F | Z | Pr (>F) |
| specimen | 49 | 0.076991 | 0.0016 | 0.816 | 5.6013 | 18.26 | 2.00E-04 |
| replicate | 1 | 0.03592 | 0.0036 | 0.038 | 12.8055 | 4.728 | 2.00E-04 |
| Residuals | 49 | 0.013745 | 0.0003 | 0.146 |  |  |  |
| Total | 99 | 0.094329 |  |  |  |  |  |
